# *Arabidopsis* ABI5 plays a role in regulating ROS homeostasis by activating *CATALASE 1* transcription in seed germination

**DOI:** 10.1007/s11103-017-0603-y

**Published:** 2017-04-08

**Authors:** Chao Bi, Yu Ma, Zhen Wu, Yong-Tao Yu, Shan Liang, Kai Lu, Xiao-Fang Wang

**Affiliations:** Center for Plant Biology, School of Life Sciences, Tsinghua University, Beijing, 100084 China

**Keywords:** *Arabidopsis thaliana*, ABI5, Catalase, ROS homeostasis, Seed germination

## Abstract

It has been known that ABA INSENSITIVE 5 (ABI5) plays a vital role in regulating seed germination. In the present study, we showed that inhibition of the catalase activity with 3-amino-1,2,4-triazole (3-AT) inhibits seed germination of Col-0, *abi5* mutants and *ABI5*-overexpression transgenic lines. Compared with Col-0, the seeds of *abi5* mutants showed more sensitive to 3-AT during seed germination, while the seeds of *ABI5*-overexpression transgenic lines showed more insensitive. H_2_O_2_ showed the same effect on seed germination of Col-0, *abi5* mutants and *ABI5*-overexpression transgenic lines as 3-AT. These results suggest that ROS is involved in the seed germination mediated by ABI5. Further, we observed that T-DNA insertion mutants of the three catalase members in *Arabidopsis* displayed 3-AT-insensitive or -hypersensitive phenotypes during seed germination, suggesting that these catalase members regulate ROS homeostasis in a highly complex way. ABI5 affects reactive oxygen species (ROS) homeostasis by affecting *CATALASE* expression and catalase activity. Furthermore, we showed that ABI5 directly binds to the *CAT1* promoter and activates *CAT1* expression. Genetic evidence supports the idea that CAT1 functions downstream of ABI5 in ROS signaling during seed germination. RNA-sequencing analysis indicates that the transcription of the genes involved in ROS metabolic process or genes responsive to ROS stress is impaired in *abi5-1* seeds. Additionally, expression changes in some genes correlative to seed germination were showed due to the change in *ABI5* expression under 3-AT treatment. Together, all the findings suggest that ABI5 regulates seed germination at least partly by affecting ROS homeostasis.

**Electronic supplementary material:**

The online version of this article (doi:10.1007/s11103-017-0603-y) contains supplementary material, which is available to authorized users.

## Introduction

Seeds are important for sustainable agriculture production and landscape biodiversity. As the first phase transition in the life cycle of higher plants, seed germination is a complex process affected by many factors. However, molecular mechanisms of seed germination are largely unknown.

Reactive oxygen species (ROS), including superoxide (^•^O_2_^−^), hydrogen peroxide (H_2_O_2_), hydroxyl radical (^•^HO), and singlet oxygen (^1^O_2_), has been believed to be connected with many biotic and abiotic stress, such as oxidative stress, drought stress, salt stress, and pathogen stress (Dat et al. 2000; Apel and Hirt 2004; Laloi et al. 2004; Torres and Dangl 2005; Miller et al. 2008). New evidence suggests that ROS is not only a cytotoxic molecule, but also emerges as a key regulator in seed physiology where ROS mediates seed germination (Bailly 2004; El-Maarouf-Bouteau et al. 2007; Bailly et al. 2008; Ye et al. 2012). An optimal range of ROS level is required for successful germination. Disruption of the ROS homeostasis will reduce the ability of seed germination (Bailly et al. 2008). The balance between ROS production and scavenging should be under elaborate and strict control for seed germination.

H_2_O_2_, a major ROS, is more stable than other ROS and is capable of long-distance diffusion across membranes, which is also admitted as the most likely ROS messenger (Möller and Sweetlove 2010). H_2_O_2_ promotes seed germination (Fontaine et al. 1994; Wang et al. 1998; Ogawa and Iwabuchi 2001) and affects the aleurone programmed cell death (PCD) of cereal grains during germination and seedling establishment (Fath et al. 2001). *Arabidopsis* catalase (CAT) forms a highly conserved enzyme family consisting of three members, *CAT1* (At1g20630), *CAT2* (At4g35090) and *CAT3* (At1g20620), which are involved in catalyzing the H_2_O_2_ decomposition to water and oxygen (Chevalier et al. 1992; Frugoli et al. 1996). CAT provides the cell with an extremely efficient mechanism of removing H_2_O_2_ because they decompose H_2_O_2_ without consuming the cellular reducing equivalents (Smykowski et al. 2010). These *CATALASE* genes exhibit different spatial and temporal expression patterns throughout the plant life cycle. Whereas CAT1 is primarily expressed in the reproductive tissues and seeds, CAT2 is strongly expressed in the photosynthetic tissue and CAT3 is ubiquitously expressed, especially in roots and young leaves (Zimmermann et al. 2006; Du et al. 2008; Mhamdi et al. 2010). It has been shown that CAT controls the concentration of ROS in cells (Foyer and Noctor 2000; Mhamdi et al. 2010). CAT activity is also influenced by other factors, such as salicylic acid and nitric oxide (Chen et al. 1993; Durner and Klessing 1996; Clark et al. 2000). Previous studies focused mainly on CAT function in plant leaves, especially in regulating the onset of leaf senescence (Smykowski et al. 2010), but whether CAT regulates seed germination is largely unknown in *Arabidopsis* .

Abscisic acid is a kind of important stress hormones, which functions in many physiological processes, including seed maturation, seed dormancy, growth and developmental regulation, and response to environmental stresses (Zeevaart and Creelman 1988; Hoffmann-Benning and Kende 1992; Wang et al. 2013). In *Arabidopsis*, ABSCISIC ACID-INSENSITIVE MUTANT 5 (ABI5), a bZIP transcription factor, plays a vital role in mediating ABA signaling during seed maturation (Finkelstein and Lynch 2000). ABI5 interacts with ABA responsive *cis*-regulatory elements of some genes, such as *EM1* and *EM6* (a class I Late Embryogenesis Abundant protein), to regulate seed maturing and seed germination (Carles et al. 2002).

It has been reported that the maize gene *CAT1* promoter contains a G-box or ABA responsive element (ABRE) and antioxidant responsive element (ARE), which indicates the important protective role of CAT in response to oxidative stresses (Polidoros and Scandalios 1999; Guan et al. 2000). GBF1, a G-box binding factor, is involved in the regulation of *CAT2* expression and intracellular H_2_O_2_ content, which directly binds to the *CAT2* promoter both in vitro and in vivo (Smykowski et al. 2010). However, the mechanism by which the *CAT1* gene transcription is regulated remains unknown so far. Could ABI5, a G-box binding protein, bind to the G-box element of *CAT1* promoter and regulate *CAT1* expression? Does ABI5 mediate the seed germination partially depending on the ROS signaling? These questions concerning the possible interrelationships between ABI5 and CAT1-mediated ROS signal are of importance but currently remain open.

In the present experiments, we showed that the catalases play a critical role in the ABI5-mediated seed germination regulation, in which ABI5 regulates *CAT1* expression directly. Furthermore, ABI5 regulates seed germination at least partly by affecting ROS homeostasis. These findings help to understand regulatory mechanisms of seed germination mediated by ABI5.

## Results

### Compared with Col-0, the seeds of *abi5* mutants showed more sensitive to 3-AT during seed germination, while the seeds of *ABI5*-overexpression transgenic lines showed more insensitive

**Fig. 1:**
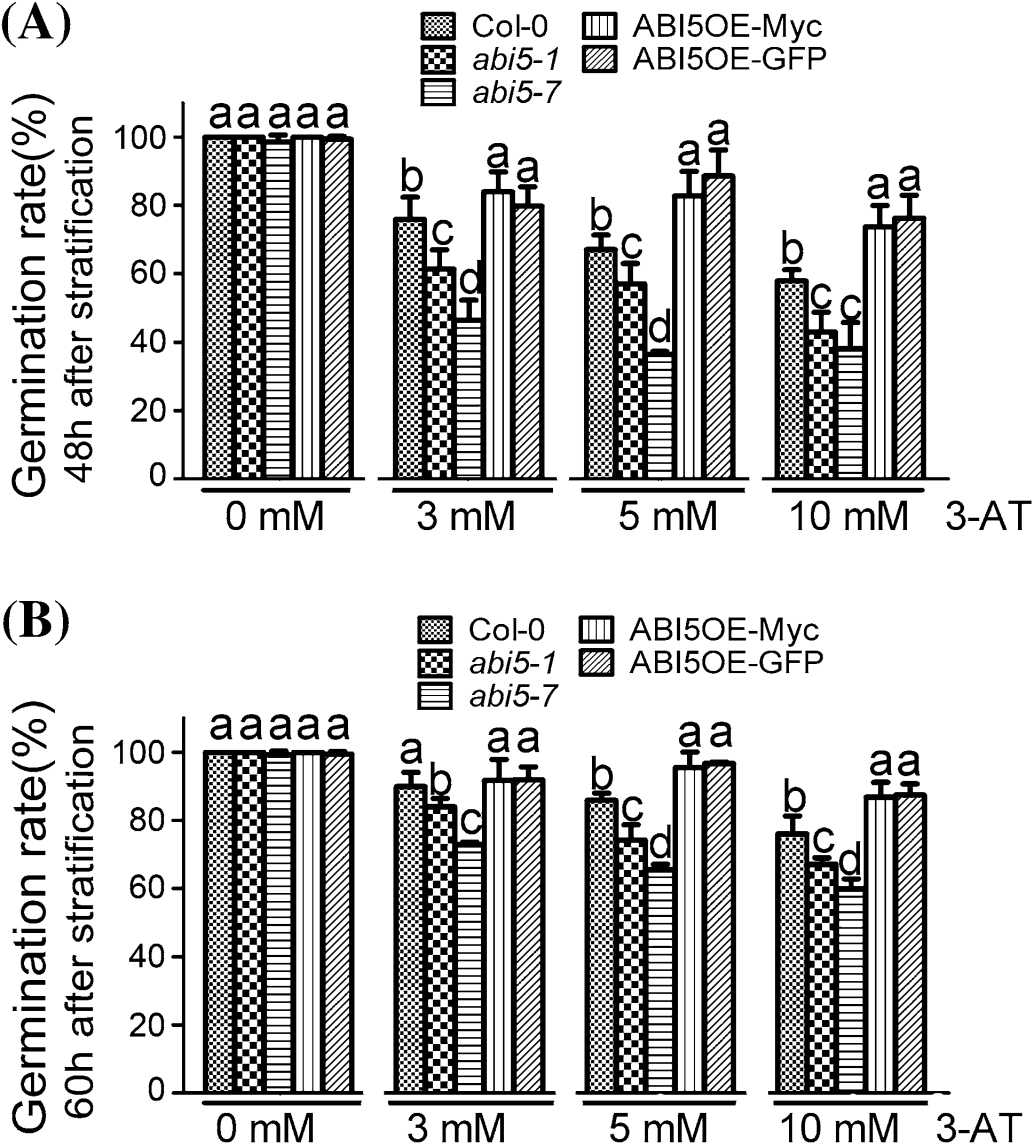
3-AT affects seed germination mediated by ABI5. Seeds of the wild-type (Col-0), overexpression transgenic lines, including ABI5OE-Myc and ABI5OE-GFP, and *ABI5* loss-of-function mutants, including *abi5-1* and *abi5-7* are grown under light conditions (16-h *light*/8-h *dark*) after stratification on MS medium supplemented with 0, 3, 5, 10 mM 3-AT. Germination rates are recorded for 48 h (**a**) and 60 h (**b**). Each value is the mean ± SE of at least three independent experiments. *Different letters* indicate significant differences at P < 0.05 (Duncan’s multiple range test) when comparing values within the same 3-AT concentration

We assessed the possible role of H_2_O_2_, a major kind of ROS, in ABI5-mediated seed germination in *Arabidopsis*. 3-amino-1,2,4-triazole (3-AT), a CAT activity inhibitor (Margoliash et al. 1960), affects ROS homeostasis by promoting H_2_O_2_ accumulation. The mutants *abi5-1* and *abi5-7*, loss-of-function mutants of *ABI5*, were used (Finkelstein 1994a, b; Nambara et al. 2002; Tamura et al. 2006; Albertos et al. 2015). When treated by different concentrations of 3-AT, including 3, 5 and 10 mM, seed germination rate of the wild-type seeds decreased. Furthermore, with the increasing of the concentration of 3-AT, the inhibition of 3-AT to seed germination were more and more significant. This result showed that 3-AT is a seed germination inhibitor (Fig. 1). In the absence of 3-AT, Col-0, *abi5* mutants and ABI5-overexpression lines (ABI5OE), including ABI5OE-GFP and ABI5OE-Myc, had the same germination rates. In the presence of 3-AT, we found that compared with Col-0, the seed germination of *abi5-1* or *abi5-7* was much slower, revealing that *abi5-1* and *abi5-7* were hypersensitive to 3-AT, but the seed germination of ABI5-overexpression lines, was much faster, revealing that ABI5OE was insensitive to 3-AT (Fig. 1). Because 3-AT is connected with catalase, we suggested that catalase probably participated in the seed germination mediated by ABI5.

### H_2_O_2_ regulates seed germination mediated by ABI5

**Fig. 2:**
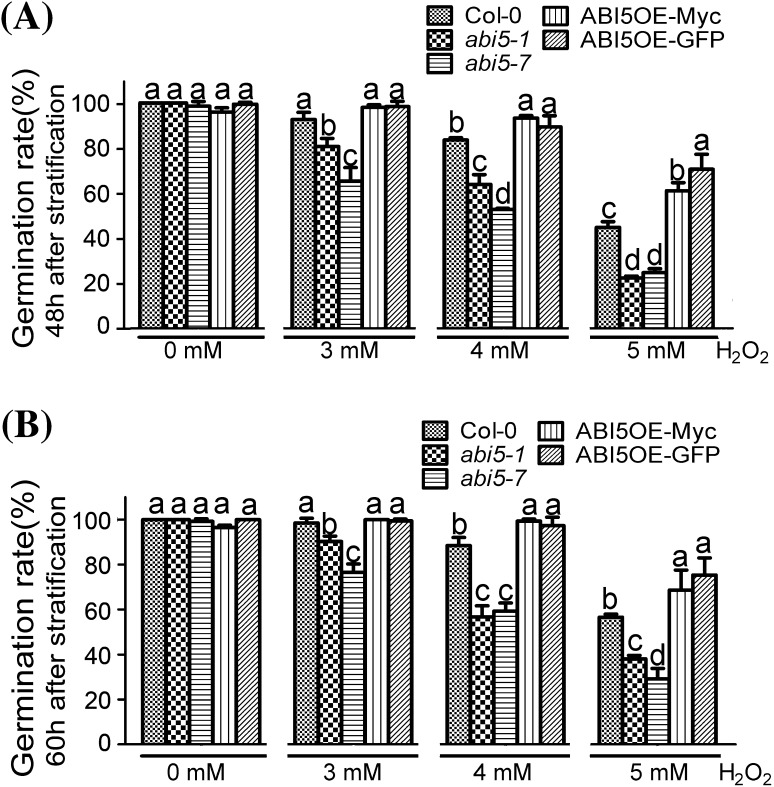
H_2_O_2_ is involved in seed germination mediated by ABI5. Seeds of the wild-type (Col-0), overexpression transgenic lines, including ABI5OE-Myc and ABI5OE-GFP, and *ABI5* loss-of-function mutants, including *abi5-1* and *abi5-7* were grown under light conditions (16-h *light*/8-h *dark*) after stratification on MS medium supplemented with 0, 3, 4, 5 mM H_2_O_2_. Germination rates are recorded for 48 h (**a**) and 60 h (**b**). Each value is the mean ± SE of at least three independent experiments. *Different letters* indicate significant differences at P < 0.05 (Duncan’s multiple range test) when comparing values within the same H_2_O_2_ concentration

It is well known that catalase is a vital kind of enzymes removing H_2_O_2_. Inhibition of catalase activity by using 3-AT will result in the change in H_2_O_2_ content. So we explored the effect of H_2_O_2_ on seed germination of Col-0, *abi5* mutants and ABI5-overexpression lines. When treated by different concentrations of H_2_O_2_, including 3, 4 and 5 mM, the wild-type seeds showed delayed seed germination, revealing that H_2_O_2_ could inhibit seed germination (Fig. 2). As same as the results of 3-AT treatment, with the increasing of the concentration of H_2_O_2_, the inhibition of H_2_O_2_ to seed germination was more and more significant. In the absence of H_2_O_2_, Col-0, *abi5* mutants and ABI5-overexpression lines, had the same germination rates. In the presence of H_2_O_2_, we found that compared with Col-0, the seed germination of *abi5-1* or *abi5-7* was much slower, revealing that *abi5-1* and *abi5-7* were hypersensitive to H_2_O_2_. As for ABI5-overexpression lines, we found that seed germination were insensitive to different concentrations of H_2_O_2_ (Fig. 2). These results showed that H_2_O_2_ was involved in the seed germination mediated by ABI5.

### CAT mutants show either insensitive or hypersensitive to the catalase activity inhibitor 3-AT

**Fig. 3:**
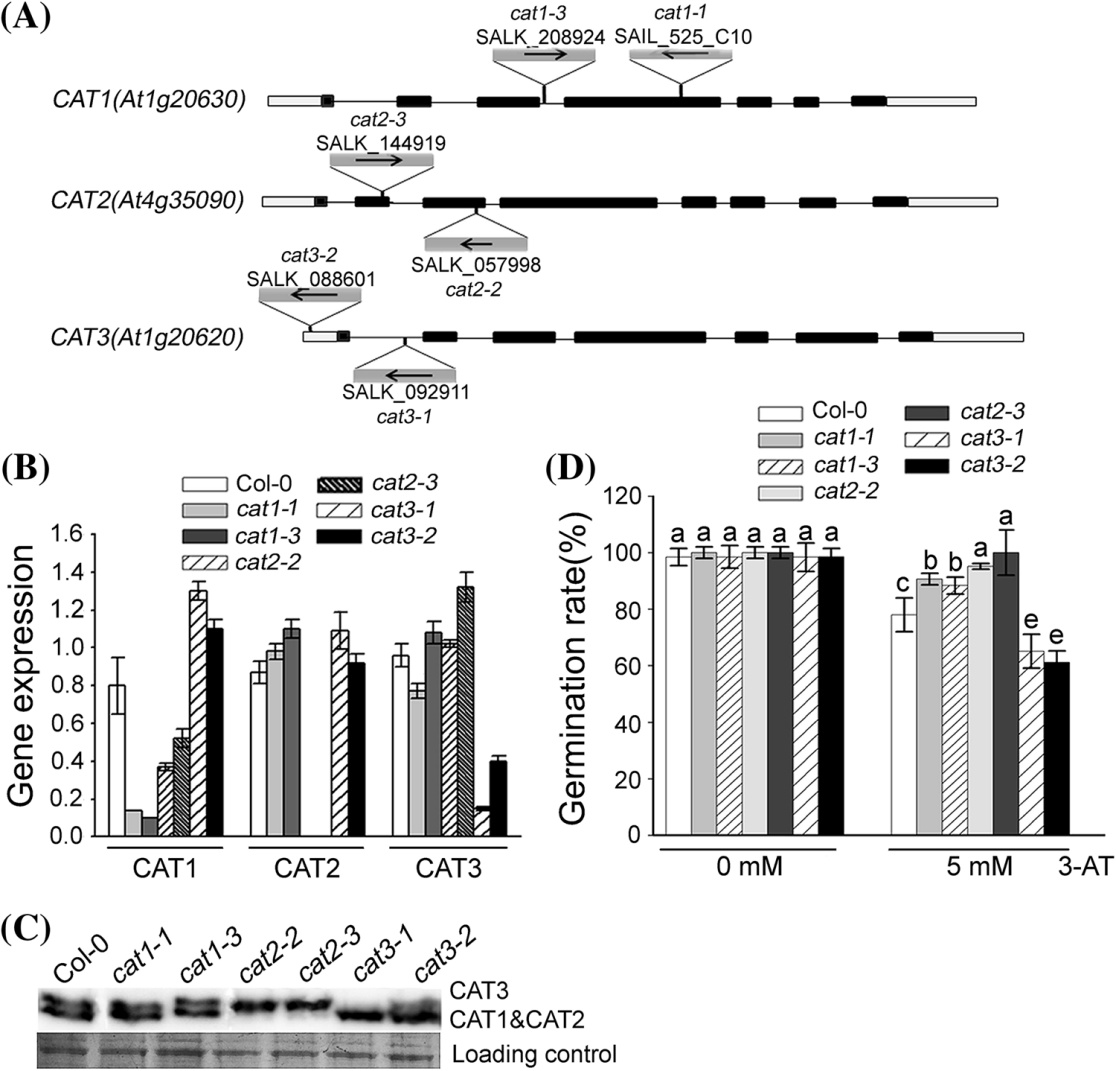
Molecular and biochemical characterizations of the *catalase* mutants. **a** Structures of the *CAT1, CAT2* and *CAT3* genes are shown with the T-DNA insertion sites in *cat1-1* (SAIL_525_C10), *cat1-3* (SALK_208924), *cat2-2* (SALK_057998), *cat2-3* (SALK_144919), *cat3-1* (SALK_092911) and *cat3-2* (SALK_088601). Exons are indicated by *black blocks*, introns by *solid lines* and untranslated regions by *grey broken lines*. The *black arrowhead* indicates the orientation of the T-DNA insertion. **b** Quantitative RT-PCR analysis of *CAT* gene expressions in wild-type and catalase mutants is shown. Each value for real-time PCR is the mean ± SE of three independent experiments. **c** Protein gel blot analysis was performed using anti-catalase serum and total proteins were extracted from seeds grown under 16-h light/8-h dark conditions for 24 h after stratification. Protein extracts were stained with Ponceau S as a loading control. The protein gel blot assay was repeated independently three times. **d** The effect of 3-AT on seed germination in *catalase* mutants. Germination rates were recorded for the wild-type, *CAT* homozygous mutants grown under light conditions (16 h *light*/8 h *dark*) for 60 h after stratification on MS medium supplemented with 0, 5mM 3-AT. Each value is the mean ± SE of three independent biological experiments, and *different letters* indicate significant differences at P < 0.05 (Duncan’s multiple range test) when comparing values within the same 3-AT concentration

To furtherly explore the function of catalase in seed germination mediated by ABI5, we examined the phenotypes of catalase members with 3-AT treatment. Considering the composition of CAT family, we identified *CAT1, CAT2* and *CAT3* mutants from the ABRC stock, including *cat1-1, cat1-3, cat2-2, cat2-3, cat3-1* and *cat3-2* (Fig. 3a). To characterize these mutants, we analyzed the CAT expression using quantitative RT-PCR and protein gel blot analysis. CAT antibodies were used to recognize three CAT members synchronously. Because CAT1 and CAT2, with the high homology and similar molecular weight, appeared in the same band in the protein gel (Hu et al. 2010; Supplementary Fig. S1), we could not distinguish CAT1 and CAT2. RT-PCR results showed that *cat1-1, cat1-3, cat3-1* and *cat3-2* were knockdown mutants, while *cat2-2* and *cat2-3* were knockout mutants (Fig. 3b). The protein gel blot analysis showed that there was no obvious change in CAT protein level in *cat1-1* and *cat1-3* mutant seeds, although *CAT1* showed remarkably decrease at RNA level (Fig. 3c). We did not detect the CAT1 and CAT2 proteins in *cat2-2* and *cat2-3* mutants, but we observed dominant increase of CAT3 expression. In *cat3-1* and *cat3-2* mutants, the increase of CAT1 and CAT2 expression was remarkable. These indicated that different CAT members had the feedback effect on other member expression.

Although CAT members had different changes in these mutants, we found that they all showed the same germination rate under normal condition. But under 5 mM 3-AT treatment, these mutants showed different sensitivities to 3-AT. The seed germination rates of *cat1-1, cat1-3, cat2-2* and *cat2-3* were much higher than that of wild-type seeds, but *cat3-1* and *cat3-2* seeds had lower germination rates compared with wild-type seeds, revealing that the seed germination of *cat1-1, cat1-3, cat2-2* and *cat2-3* was insensitive to 3-AT, while the seed germination of *cat3-1* and *cat3-2* was hypersensitive to 3-AT (Fig. 3d). These different phenotypes of seed germination suggested that CAT members may play different roles in ROS signaling during seed germination.

### Change in *ABI5* expression negatively affects CAT protein level, while 3-AT does not affect CAT protein level in the different genotypes

**Fig. 4:**
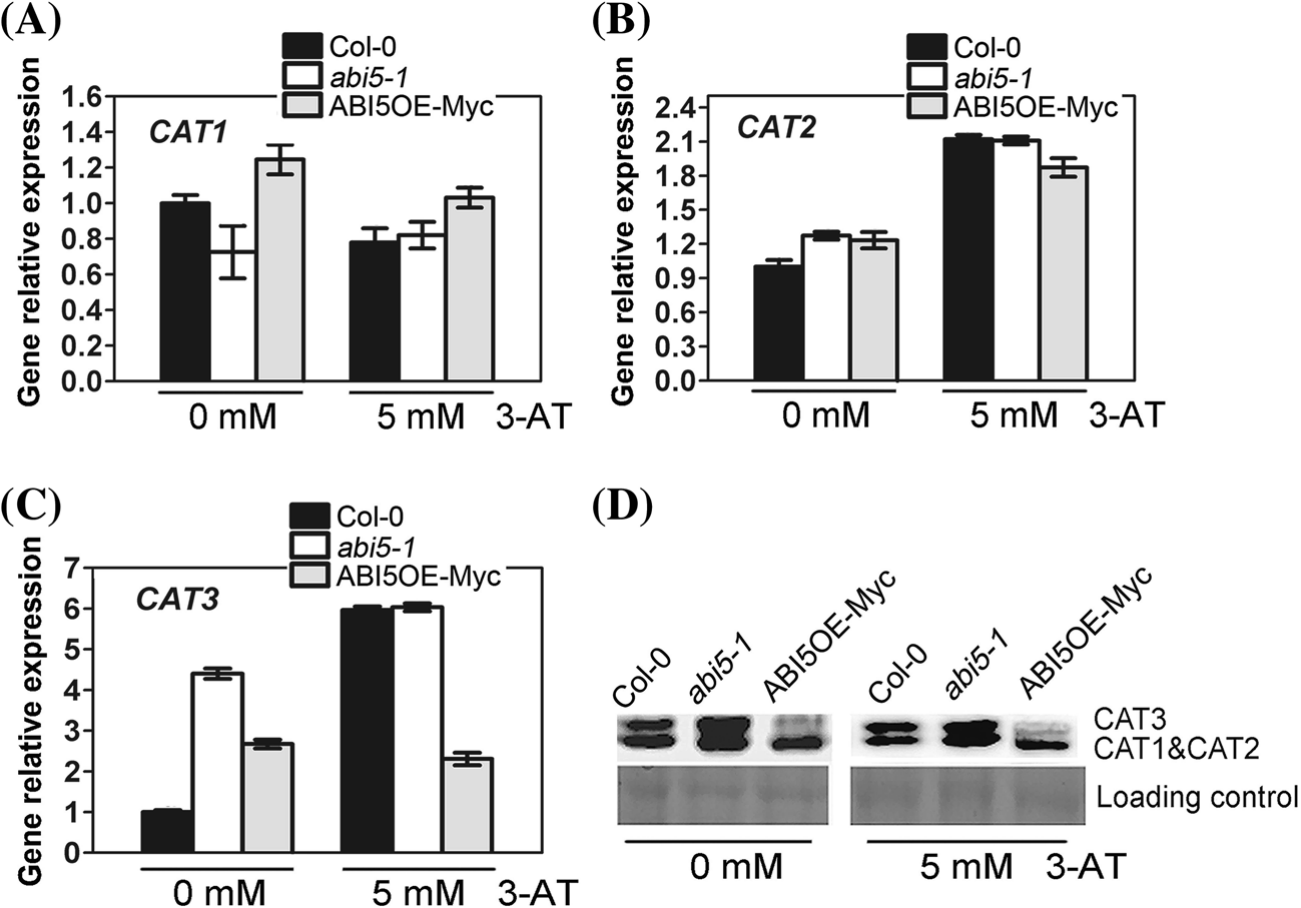
Expression analysis of CAT members in the seeds of Col-0, ABI5OE-Myc and *abi5-1*. The seeds of Col-0, ABI5OE-Myc and *abi5-1* were grown under 16-h light/8-h dark conditions for 24 h after stratification on MS medium supplemented with 0 or 5mM 3-AT. **a–c** are quantitative RT-PCR analysis of *CAT1* (A), *CAT2* (B) and *CAT3* (C), respectively. Each value is the mean ± SE of three independent experiments. **d** Protein gel blot analysis of the expression of CAT in the seeds of Col-0, ABI5OE-Myc and *abi5-1*. Protein extracts were stained with the Ponceau S as a loading control. The assay was independently repeated three times

Because the capacity of catalase decomposing H_2_O_2_ is decided by catalase activities and CAT protein levels, H_2_O_2_ content should be affected by the two factors. Firstly, we checked the expressions of CAT at RNA and protein levels in seeds of wild-type Col-0, *abi5-1* and ABI5OE-Myc, respectively. We found that the expression of *CAT1* and *CAT3* changed significantly at RNA level in *abi5-1* seeds without 3-AT treatment (Fig. 4a–c). *CAT1* expression was down-regulated, while *CAT3* expression was up-regulated. Interestingly, expression of *CAT3* also showed up-regulated in ABI5OE-Myc seeds. As for *CAT2*, there was no obvious changes in *abi5-1* and ABI5OE-Myc compared with Col-0. 3-AT treatment had the different effect on expression of *CAT* members in Col-0, *abi5-1* and ABI5OE-Myc. It inhibited *CAT1* expression in Col-0 and ABI5OE-Myc, while induced *CAT2* expression in Col-0, *abi5-1* and ABI5OE-Myc, and *CAT3* expression in Col-0 and *abi5-1*. It seemed that the change in *ABI5* expression could regulate the expression of CAT members with complicate mechanism.

We also detected CAT proteins in seeds of Col-0, *abi5-1* and ABI5OE-Myc. Compared with Col-0, CAT protein level increased in the seeds of *abi5-1*, while decreased in the seeds of ABI5OE-Myc without 3-AT treatment (Fig. 4d), indicating that *ABI5* mutation affects the CAT protein level. On the other hand, we examined the protein levels of the CAT members under 5mM 3-AT treatment in the seeds of Col-0, *abi5-1* and ABI5OE-Myc, and found that 3-AT did not affect the CAT protein levels (Fig. 4d). Here we found that expression changes in CAT members were not synchronous completely between RNA and protein level. For example, compared with Col-0, *CAT1* showed down-regulated and *CAT2* showed no changes in abi5-1 at RNA level; whereas the sum quantity of CAT1 and CAT2 showed a significant increase at protein level. There should be a complicated mechanism on maintaining the balance of CAT members at post-transcription and translation level.

Although 3-AT had no obvious effects on the expression of CAT members at protein level, the difference of their expressions among Col-0, *abi5-1* and ABI5OE-Myc should be responsible for the corresponding phenotypes with 3-AT treatment.

### 3-AT inhibits catalase activities dependently of ABI5 expression

**Fig. 5:**
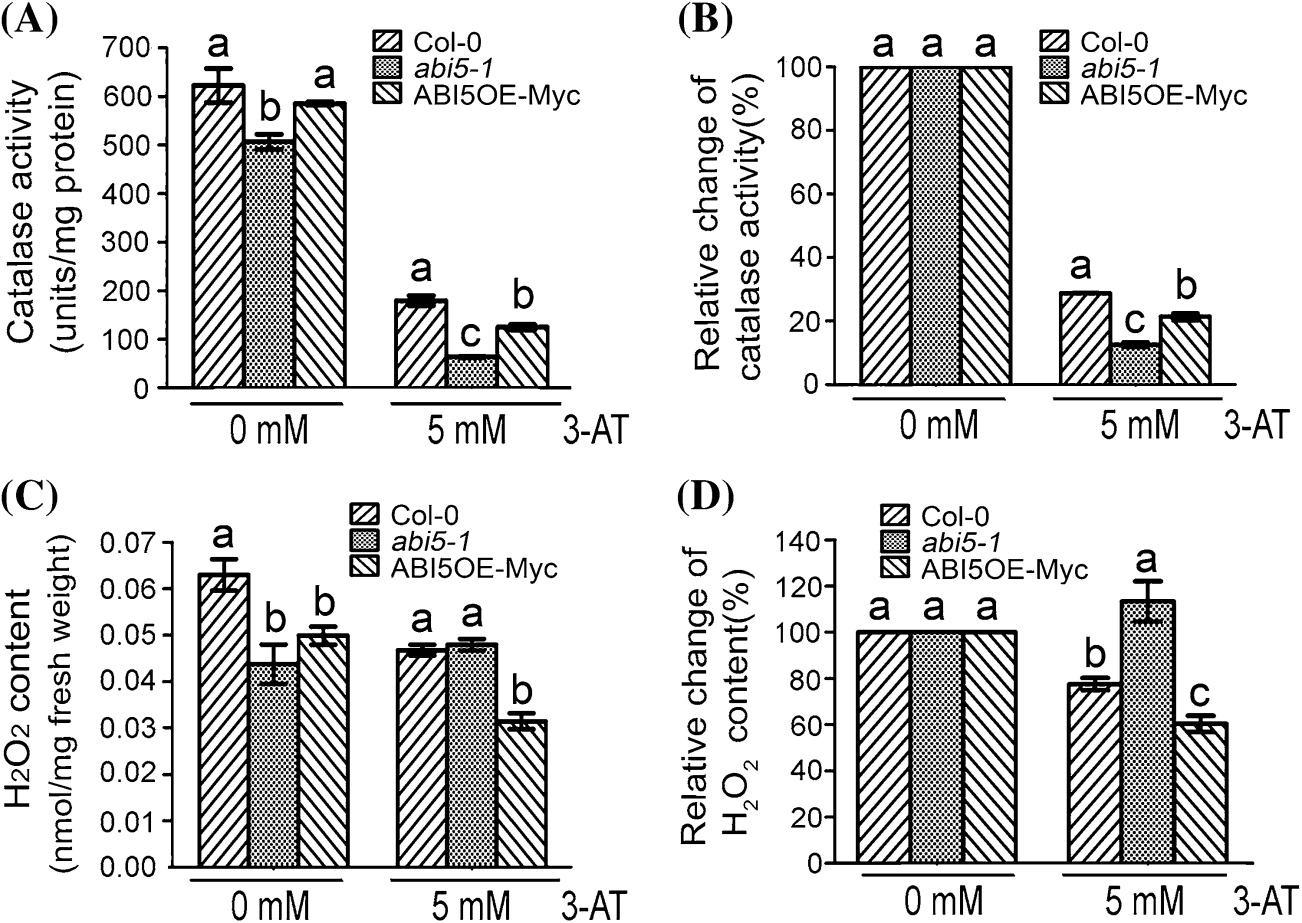
Changes in catalase activity and H_2_O_2_ content in seeds of Col-0, ABI5OE-Myc and *abi5-1*. **a** Measurement of the absolute catalase activity in seeds of Col-0, ABI5OE-Myc and *abi5-1* with or without 5 mM 3-AT treatment grown under 16-h light/8-h dark conditions for 24 h after stratification. Each value is the mean ± SE of three independent biological experiments, and *different letters* indicate significant differences at P < 0.05 (Duncan’s multiple range test) when comparing values within the same 3-AT concentration. **b** Analysis of the relative change in catalase activity in seeds of Col-0, ABI5OE-Myc and *abi5-1*. The* vertical axis* represents the relative change in the absolute value of catalase activity in Col-0, ABI5OE-Myc and *abi5-1* seeds in the presence of 5 mM 3-AT treatment in relative to that in the absence of 3-AT, respectively. The absolute values of catalase activity in the seeds of Col-0, ABI5OE-Myc and *abi5-1* without 3-AT treatment are taken as a control (100%), respectively. Each value is the mean ± SE of three independent biological experiments, and *different letters* indicate significant differences at P < 0.05 (Duncan’s multiple range test) when comparing values within the same 3-AT concentration. **c** Measurement of the absolute H_2_O_2_ content in seeds of Col-0, ABI5OE-Myc and *abi5-1* with or without 5 mM 3-AT treatment grown under 16-h light/8-h dark conditions for 24 h after stratification. Each value is the mean ± SE of three independent biological experiments, and *different letters* indicate significant differences at P < 0.05 (Duncan’s multiple range test) when comparing values within the same 3-AT concentration. **d** Analysis of the relative change in the H_2_O_2_ content in seeds of Col-0, ABI5OE-Myc and *abi5-1*. The *vertical axis* represents the relative change in H_2_O_2_ content of the absolute value of H_2_O_2_ content of Col-0, ABI5OE-Myc and *abi5-1* with 5 mM 3-AT treatment in relative to that without 5 mM 3-AT treatment, respectively. The absolute values of H_2_O_2_ content in the seeds of Col-0, ABI5OE-Myc and *abi5-1* without 3-AT treatment are taken as a control (100%), respectively. Each value is the mean ± SE of three independent biological experiments, and *different letters* indicate significant differences at P < 0.05 (Duncan’s multiple range test) when comparing values within the same 3-AT concentration

It has been reported that 3-AT is a catalase inhibitor, so we assayed catalase activities of different genotypes in the presence or absence of 3-AT. In the absence of 3-AT, catalase activity of *abi5-1* seeds was lower than that of Col-0 and ABI5OE-Myc (Fig. 5a, b), but there was no difference of the phenotype in seed germination among them (Figs. 1, 2). This suggested that although catalase protein and activity were different in Col-0, *abi5-1* and ABI5OE-Myc seeds without 3-AT treatment, these differences were not enough to affect ROS homeostasis which can influence seed germination. Under 3-AT treatment, the catalase activity in Col-0, *abi5-1* and ABI5OE-Myc seeds were all obviously inhibited, showing that 3-AT could function as the catalase activity inhibitor (Fig. 5a, b). Furthermore, we found that the inhibition of catalase activity in *abi5-1* seeds was the most serious (Fig. 5a, b). We suggested that the most serious inhibition of catalase activity was connected with the postponed germination of *abi5-1* seeds.

### 3-AT affects H_2_O_2_ content in seeds of wild-type, *abi5-1* and ABI5OE-Myc plants in a different way

The capacity of catalase scavenging H_2_O_2_ is correlated with the catalase protein and catalase activity, which codetermine the H_2_O_2_ content. The lower catalase activity can induce H_2_O_2_ accumulation, and higher CAT protein accumulation can promote H_2_O_2_ decomposition. The final H_2_O_2_ content should be decided by which effect was dominant. We measured H_2_O_2_ content in seeds of Col-0, *abi5-1* and ABI5OE-Myc. Without 3-AT treatment, H_2_O_2_ content in both *abi5-1* and ABI5OE-Myc seeds were lower than that in Col-0 seeds (Fig. 5c, d). Although the fundamental H_2_O_2_ content was different in Col-0, *abi5-1* and ABI5OE-Myc seeds in the absence of 3-AT, the difference did not influence seed germination (Figs. 1, 2). When using 3-AT treatment, the CAT protein level was not influenced, but the catalase activity was inhibited obviously. We conjectured that there would be H_2_O_2_ accumulation in the seeds of Col-0, *abi5-1* and ABI5OE-Myc. Actually the result showed that except for *abi5-1*, which showed H_2_O_2_ accumulation, both Col-0 and ABI5OE-Myc showed H_2_O_2_ decrease in the presence of 3-AT (Fig. 5c, d). Furthermore, in comparison to the control panels without 3-AT treatment respectively, we found that H_2_O_2_ content in the seeds of *abi5-1* accumulated obviously with 3-AT treatment, while H_2_O_2_ content decreased in the seeds of Col-0 and ABI5OE-Myc. The relative decrease of H_2_O_2_ content in ABI5OE-Myc seeds was more remarkable than that in Col-0 seeds (Fig. 5c, d). So we suggested that the relative changes in H_2_O_2_ content with 3-AT treatment were connected with the different phenotypes of seed germination in Col-0, *abi5-1* and ABI5OE-Myc.

### ABI5 binds to the promoter of *CAT1*

**Fig. 6:**
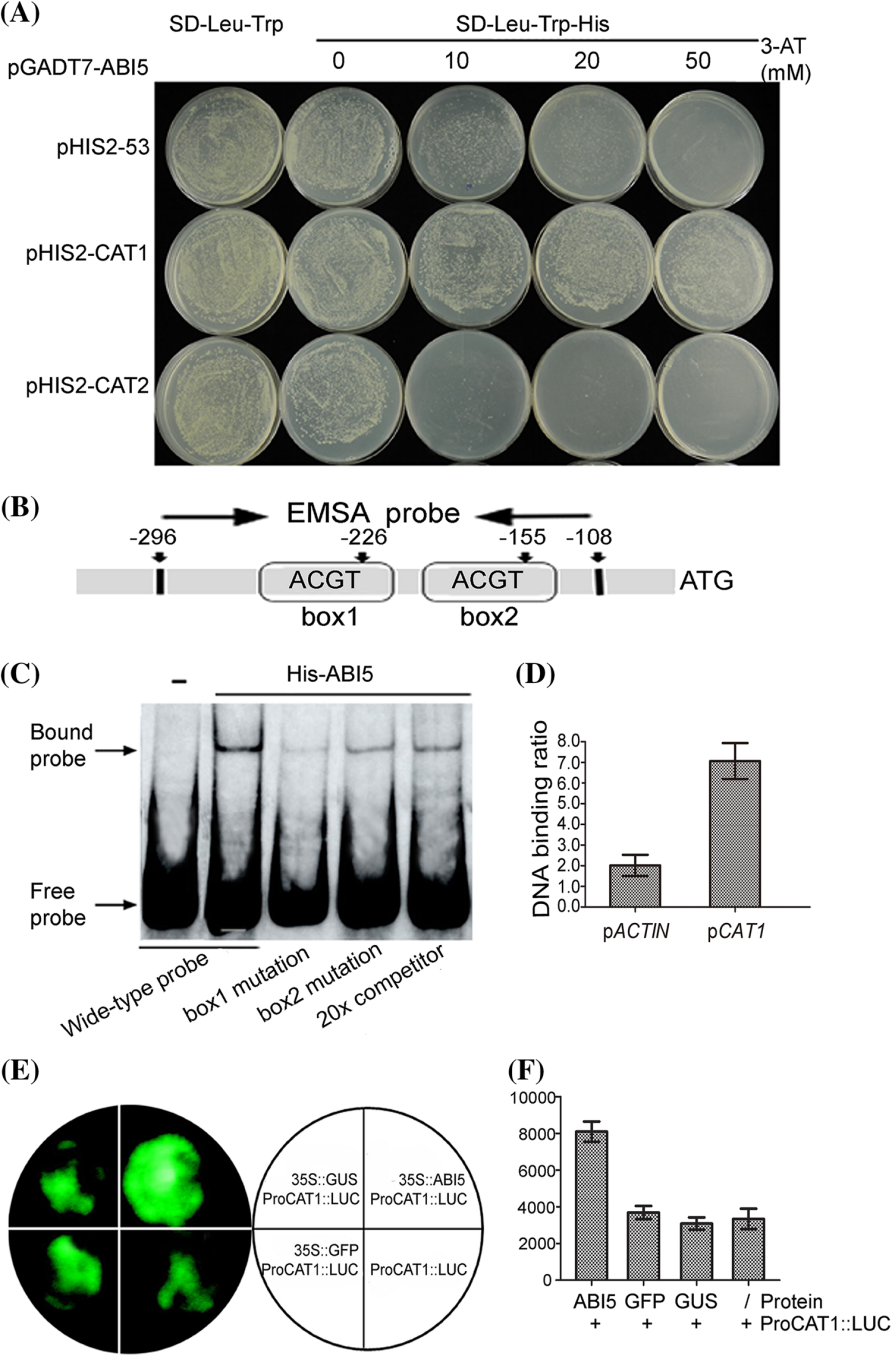
ABI5 can directly bind to the promoter of *CAT1* in vitro and activate *CAT1* expression in vivo. **a** Yeast one-hybrid assay. The prey vector harboring ABI5 (indicated by pGADT7-ABI5) and the bait vector pHIS2 harboring *CAT1* promoter (indicated by pHIS2-CAT1) were used to co-transform yeast cells. The transformation with vectors pHIS2-53 and pHIS2-CAT2 was taken as a negative control. The experiments were repeated three times with the same results. **b** The promoter structure of *CAT1* gene. The *box1* and *box2* denote two G-box from left to right with sequence sites relative to the star code. The fragment from −296 to −108 indicates the sequence used in electrophoretic mobility shift assay described in the *panels*. **c** Electrophoretic mobility shift assay shows that ABI5 can bind the *box1* site and *box2* site. The 6His tag peptide (6His), which was fused to ABI5 protein for ABI5 protein production. “-” represents that 6His was served as a negative control. Box1 mutation is ACGT→TTTT, and box2 mutation is ACGT→TTTT (see Methods section). Wild type probe, not mutation labeled probe; box1 mutation, box1 mutation labeled probe; box2 mutation, box2 mutation labeled probe; 20×, 20-fold unlabeled probes as competitors. *Black arrow* means shift band. These experiments were repeated six times substantially with the same results. **d** ChIP-qPCR assays were performed using the specific primer corresponding to the *CAT1* promoter region. The ACTIN promoter was used as a negative control. Primers used in the ChIP-qPCR assays are indicated by *arrows* and are presented in Fig. 6b. Each data *bar* represents mean ± SE (n > 3). Similar results were obtained from four independent experiments. **e** Test of the interactions of ABI5 with the *CAT1* promoter in vivo in tobacco leaves. The tobacco leaves were transformed with the following constructs all together with ProCAT1::LUC. The control constructs harbor GFP and GUS encoding open reading frame, called “35S::GFP” and “35S::GUS” respectively. Co-expressed the constructs of “35S::ABI5” with ProCAT1::LUC can active the activity of ProCAT1::LUC, but controls cannot. **f** Quantitative analyses of luminescence intensity are shown in **e**. The panels show the corresponding quantitative data corresponding to fluorescent images. Each value is the mean ± SE with five independent determinations. The experiments were performed three biological repeats and obtained the similar trend

How does ABI5 affect ROS homeostasis? Previous studies have demonstrated that ABI5 is a versatile transcription factor, binding to the G-box (ACGT) motif to promote the expression of *EM1* and *EM6* (Carles et al. 2002). Using PlantCARE (http://bioinformatics.psb.ugent.be/webtools/plantcare/html/) to analyze the sequence of *AtCAT1* promoter, we found two G-box elements within the 442 bp sequence upstream of the *AtCAT1* start codon. Combined with previous evidence on that *CAT1* expression was down-regulated in *abi5-1* at RNA level (Fig. 4a), we were interested in whether ABI5 could affect ROS homeostasis by regulating *CAT1* transcription. We detected interaction of the ABI5 with the promoter of *CAT1* in a yeast one-hybrid system. Yeast cells which were co-transformed with both a ABI5-encoding cDNA and *CAT1* promoter could grow in a selection SD medium (lacking Trp, Leu and His nutrients, and containing 3-AT), indicating a potential interaction between the ABI5 and *CAT1* promoter (Fig. 6a). As a negative control, yeast cell which were co-transformed with both control vector (p53) and *CAT2* promoter could not grow in this medium (Fig. 6a). These data suggested that ABI5 could potentially bind to the promoter of *CAT1* (Fig. 6a).

To investigate whether ABI5 could directly regulate *CAT1* expression, we used electrophoretic gel mobility shift assay (EMSA) with the purified recombinant ABI5 protein. The full length ABI5 protein was overexpressed with a C-terminal 6 residue histidine fusion in *E. coli*. Most of the recombinant proteins were expressed in the soluble fraction and purified by nickel ion chelate chromatography. We analyzed the *CAT1* promoter described in Fig. 6b and found two G-box in its promoter region. The probe fragment of p*CAT1*, which is 189 bp long (−296/−108) and contains two G-box, named box1 and box2, was chosen (Fig. 6b). The mutant probe fragments included box1 mutation and box2 mutation, respectively. The EMSA results indicated that ABI5 binds specially to the pCAT1 probe. Both box1 mutation and box2 mutation could weaken the binding between ABI5 protein and *CAT1* promoter (Fig. 6c).

Furtherly, we assayed the interaction of ABI5 with the same *CAT1* promoter fragment as EMSA assay by ChIP analysis combined with quantitative real-time PCR. We observed that ABI5 can bind the *CAT1* promoter (Fig. 6d). This result was consistent with the result of the above-described EMSA assay (Fig. 6c), and revealed that ABI5 can interact with the *CAT1* promoter in vivo.

### ABI5 activates *CAT1* transcription in vivo

The evidence from yeast one-hybrid, EMSA and ChIP analysis indicated that ABI5 could bind to the *CAT1* promoter. Thus in vivo luciferase complementation imaging assay (LCI) was employed to investigate whether ABI5 could active *CAT1* expression in vivo. By analyzing the promoter sequences, the reporter plasmid Pro-CAT1-LUC and the effector plasmid pCAMBIA-ABI5-Myc were constructed separately. When Pro-CAT1-LUC was transfected into the *N. benthamiana* tobacco leaves together with pCAMBIA-ABI5-Myc, strongly LUC activity was detected (Fig. 6e, f). However, when the pCAMBIA-ABI5-Myc was substituted by the equal amount of pCAMBIA-GFP or pCAMBIA-GUS control vector, the LUC activity decreased obviously (Fig. 6e, f). These results revealed that ABI5 can active *CAT1* transcription in vivo.

### The *CAT1* mutation suppresses 3-AT-hypersensitive phenotype of the loss-of-function mutant *abi5-1* in seed germination

**Fig. 7:**
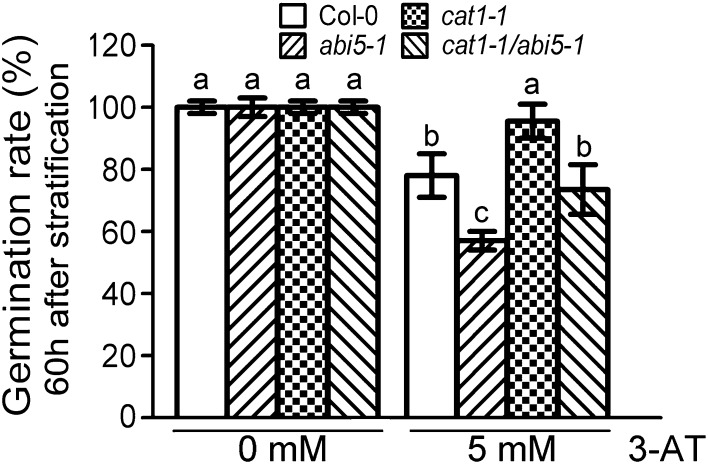
Genetic evidence supports that CAT1 plays a role directly of ABI5 downstream. The *cat1* mutation can suppress 3-AT hypersensitive phenotype of *abi5-1* in seed germination. Seed germination rate of the wild-type plants (Col-0) and mutants, including *cat1-1, abi5-1*, and *cat1-1 abi5-1* was recorded in 3-AT free medium and 3-AT containing medium (5 mM) at 60 h after stratification. Each value is the mean ± SE of five independent biological determinations, and *different letters* indicate significant differences at P < 0.05 (Duncan’s multiple range test) when comparing values within the same 3-AT concentration

Above evidence had showed that ABI5 can bind to the promoter of *CAT1* and active its transcription (Fig. 6). Subsequently, we explored the genetic function of CAT1 in ROS signaling mediated by ABI5. As shown in Figs. 1, 3d, *abi5* mutants showed hypersensitive to 3-AT during seed germination, while *cat1* mutants showed insensitive to 3-AT. So we suggested that ABI5 and CAT1 played opposite roles in seed germination, respectively. Based on above reason, we generated double mutants of *cat1-1abi5-1* by crossing. When *cat1* mutation was introduced into *abi5-1* mutant, the hypersensitive phenotype in seed germination of *abi5-1* was recovered (Fig. 7). These data provided the genetic evidence that CAT1, as a direct target of ABI5, functioned downstream of ABI5 in ROS signaling. Besides, we also noticed that *cat1-1 abi5-1* did not show the same phenotype as *cat1-1* did under 3-AT treatment, which meant that *CAT1* mutation could not completely block the pathway mediated by ABI5, we suggested that CAT1 was one of the targets of ABI5 during seed germination regulated by ROS.

### Disruption of *ABI5* alters the expression of genes related to ROS metabolism and ROS signaling in seed germination

**Fig. 8:**
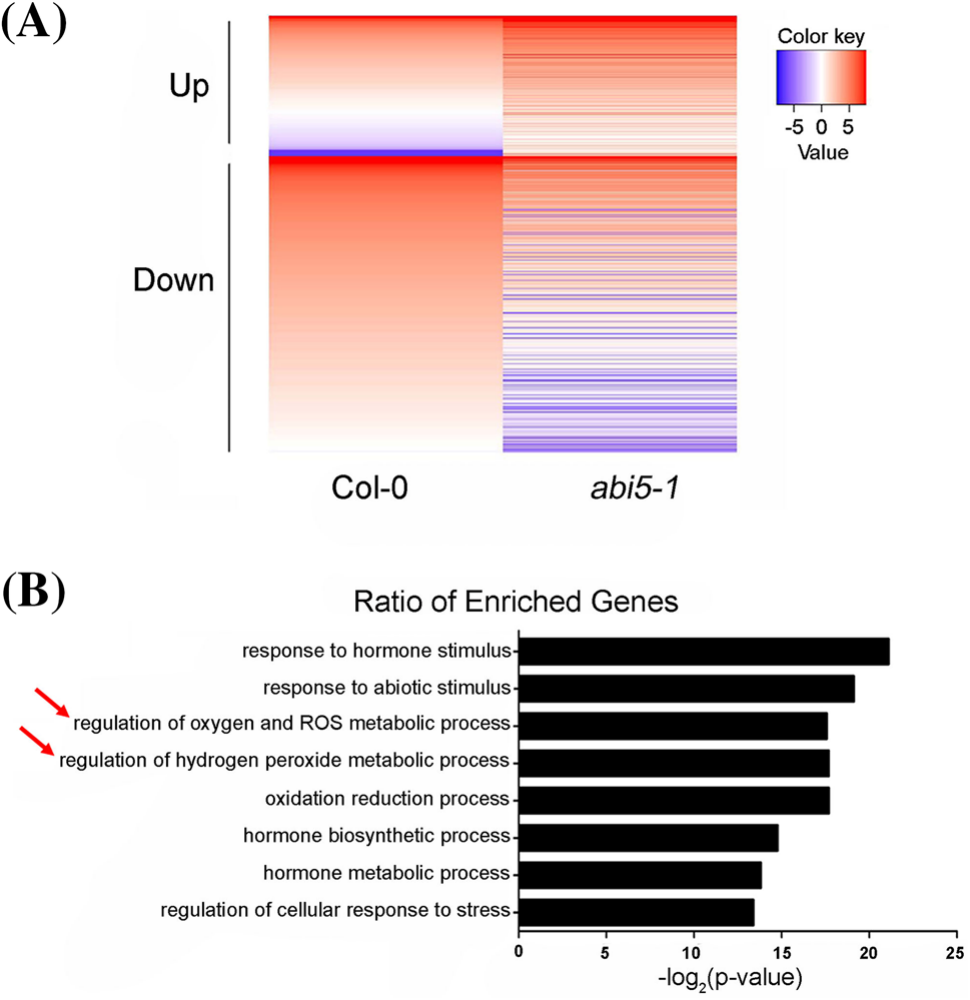
RNA sequencing analysis of differentially expressed transcripts in the seeds of wild-type (Col-0) and knockout mutant *abi5-1*. **a** *Diagrams* show the number of the differentially expressed genes up- or down-regulated in Col-0 and *abi5-1* mutant. The heatmap shows the differentially expression of 616 up-regulated genes and 1303 down-regulated genes with a cut-off value = twofold or 0.5-fold (FDR ≤ 0.05). All comparisons were made relative to the gene expression level of the wild type (Col-0). **b** GO analysis of 1303 down-regulated genes. *Diagrams* show the functional distribution of the ratio of enrichment genes among 1303 down-regulated genes. *Red arrows* show ROS-related genes

To further confirm that ABI5 functioned as a ROS regulator during seed germination, we analyzed the effect of the *abi5* mutation on the expression of ROS-responsive genes in seed germination. Total RNAs were extracted from the seeds of wild-type and *abi5-1*. We performed RNA-sequencing (RNA-seq) analyses on wild-type and *abi5-1*. The differently expressed genes in Col-0 and *abi5-1* were analyzed. A total of 16,561 genes were found in Col-0 seeds, and of 16,116 genes in *abi5-1* seeds (Supplemental Fig. S2A). Using the twofold change and the false discovery rate (FDR), 0.05 as the p-value cutoff for selecting the differentially expressed transcripts, lots of differentially expressed genes (DEGs) were found (Fig. 8a; Supplemental Fig. S2b). Among of these DEGs, there were 616 genes which were induced and 1303 genes which were repressed in *abi5-1* seeds (Supplemental Tables S9, S10).

In this study, we focused on the genes that are responsible for ROS metabolism or in response to ROS. The Gene Ontology (GO) analysis indicated that among these down-regulation DEGs, there were many genes which participate in oxygen and ROS metabolic process and regulation of H_2_O_2_ metabolic process (Fig. 8b). About 10% of the DEGs involved in oxygen and ROS metabolic process showed obviously down-regulation, including AT WRKY40 (AT1G80840), an peroxisomal NAD-malate dehydrogenase (AT5G09660), and so on (Table 1). Furthermore, among 34 DEGs related to oxygen and reactive oxygen species metabolic process, there were 25 genes involved in H_2_O_2_ metabolic process. Some genes in response to oxidative stress were up-regulated in *abi5-1*, which might be the feedback due to *abi5* mutation impairing ROS signaling by inactivating ROS regulators (Table 1). Besides, due to the disruption of *ABI5*, expressions of some genes, participating in GA metabolism, GA signaling, ABA metabolism and ABA signaling, changed significantly, showing that they were involved in the process of ABI5 regulating seed germination (Table 2).

**Table 1:** Go analysis of differentially expressed genes in Col-0 and *abi5-1*

Gene set name(no. genes)	Description	Gene number in overlap	Fold change (*abi5-1*/Col-0)	Gene names				
OXYGEN_AND_REACTIVE_OXYGEN_SPECIES_METABOLIC_PROCESS(347)	GO:0006800 oxygen and reactive oxygen species metabolic process, GOslim:biological_process	34	Down-regulation	AT5G02790	AT3G55800	AT1G80840	AT4G13510	AT1G19670
				AT4G16190	AT5G09660	AT1G33590	AT4G33560	AT2G20570
				AT5G24530	AT3G09940	AT2G36470	AT2G26400	AT1G32060
				AT1G59870	AT1G77120	AT1G74710	AT4G33070	AT1G02220
				AT1G20020	AT5G20480	AT2G28200	AT5G66570	AT5G21090
				AT3G28930	AT3G14150	AT5G60360	AT2G44490	AT1G68520
				AT1G54410	AT1G66880	AT1G18570	AT1G66200	
REGULATION_OF_HYDROGEN_PEROXIDE_METABOLIC_PROCESS(187)	GO:0010310 regulation of hydrogen peroxide metabolic process, GOslim:biological_process	25	Down-regulation	AT1G80840	AT1G59870	AT3G28930	AT1G32060	AT2G44490
				AT5G24530	AT1G20020	AT4G33560	AT5G20480	AT1G18570
				AT5G09660	AT5G66570	AT2G20570	AT4G33070	AT1G66880
				AT1G33590	AT1G74710	AT1G19670	AT2G36470	AT1G54410
				AT4G13510	AT1G77120	AT3G55800	AT2G26400	AT1G68520
RESPONSE_TO_OXIDATIVE_STRESS (582)	GO:0006979 response to oxidative stress, GOslim:biological_process	22	Up-regulation	AT1G60970	AT2G31570	AT5G64510	AT5G16960	AT2G39800
				AT2G40880	AT1G71000	AT2G22080	AT5G39610	AT1G14200
				AT4G33940	AT4G08770	AT1G13340	AT1G69270	AT3G60980
				AT1G35720	AT5G40390	AT5G16990	AT4G34890	AT1G50290
				AT2G47180	AT1G09080			

P-value < 0.05; Differentially expressed genes twofold-down-expression change or twofold-up-expression change

**Table 2:** Differentially expressed genes related to seed germination and ROS metabolism in Col-0 and *abi5-1*

Gene_ID	Gene names	Fold change (*abi5-1*/Col-0)	Up/Down	Gene description	Pathway
AT1G15550	GA3OX1	0.158792	Down	Gibberellin 3-oxidase 1	GA biosynthesis
AT1G66350	RGL1	0.394985	Down	RGA-like 1	GA signaling
AT5G45830	DOG1	8.766422	Up	Delay of germination 1	Seed development
AT3G51810	EM1	0.169394	Down	Stress induced protein	
AT2G40170	EM6	0.477625	Down	Stress induced protein	
AT1G78390	NCED9	2.70285	Up	Nine-cis-epoxycarotenoid dioxygenase 9	ABA metabolism
AT2G40330	PYL6	0.450936	Down	PYR1-like 6	ABA signaling
AT2G26040	PYL2	0.493138	Down	PYR1-like 2	
AT2G38310	PYL4	0.410952	Down	PYR1-like 4	
AT4G01026	PYL7	5.927423	Up	PYR1-like 7	
AT3G19290	ABF4	3.243769	Up	ABRE binding factor 4	
AT5G57050	ABI2	2.09312	Up	Protein phosphatase 2C family protein	
AT2G31570	GPX2	3.094566	Up	Glutathione peroxidase 2	ROS metabolism
AT4G11600	GPX6	0.483308	Down	Glutathione peroxidase 6	
AT1G77490	APXT	2.813915	Up	Thylakoidal ascorbate peroxidase	
AT2G35690	ACX1.2	4.115625	Up	Acyl-CoA oxidase 5	

P-value < 0.05; Differentially expressed genes twofold-down-expression change or twofold-up-expression change

Surprisingly, we did not find *CAT1* transcript in the DEGs. It seemed to contradict with the result of RT-PCR (Fig. 4a). It has been well known that, because of the technical biases in RNA-seq library generation and sequencing, false positive and false negative results will be produced in RNA-Seq data (Langmead et al. 2009; Zheng and Moriyama 2013). On the other hand, absence of *CAT1* transcript in DEGs could be connected with possible differences in sampling between the RT-PCR and RNA-SEq. To ensure the reliability of our RNA-Seq data, we analyzed the expressions of ABI5 target genes in RNA-Seq data, including *EM1* and *EM6* (Carles et al. 2002). Both *EM1* and *EM6* showed down-regulation in *abi5-1*, which suggested that our RNA-Seq results were reliable (Supplemental Tables S11).

**Fig. 9:**
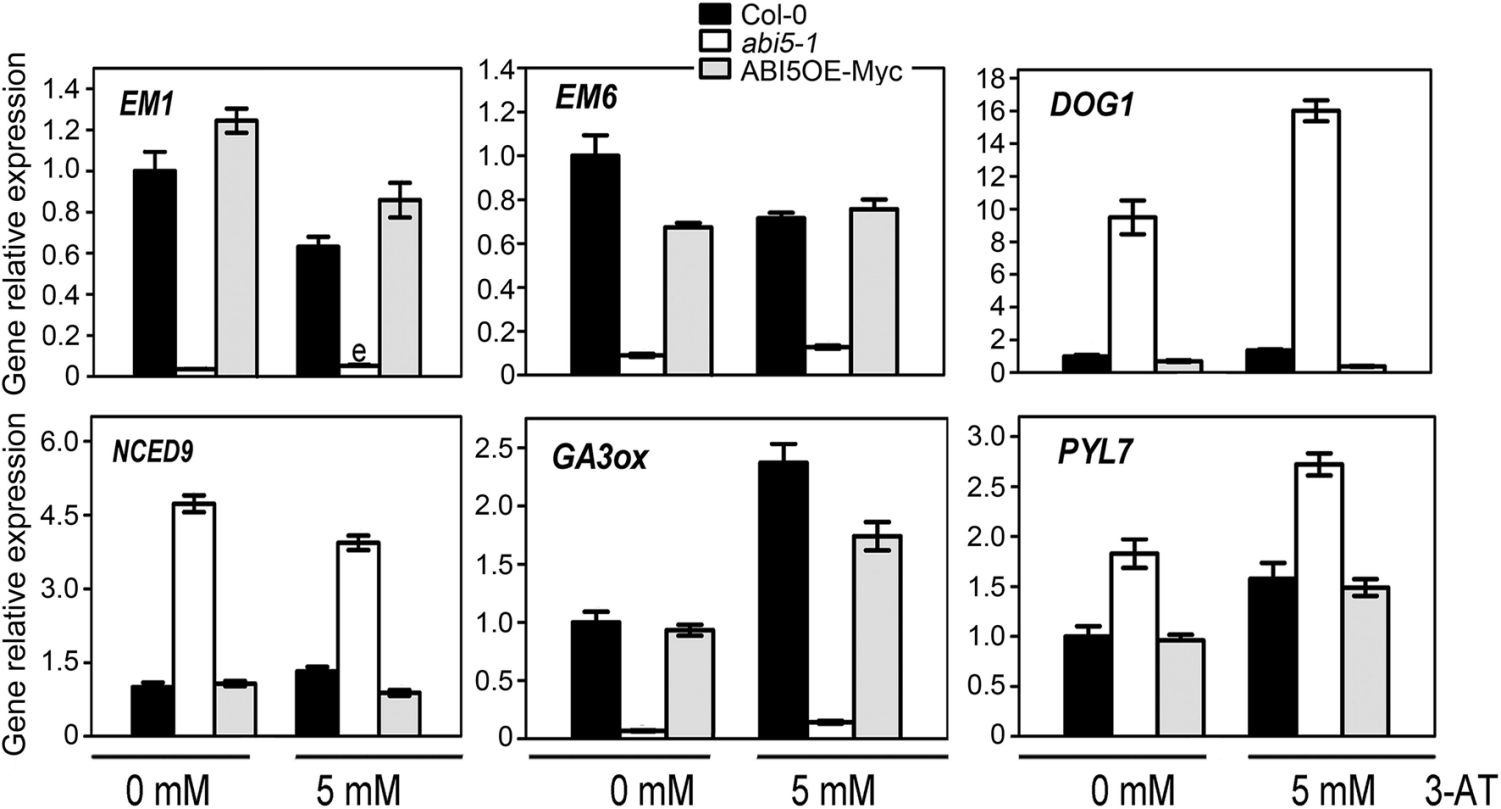
3-AT alters the expressions of genes related to seed germination due to *ABI5* disruption and overexpression. Seeds of Col-0, ABI5OE-Myc and *abi5-1*, were treated with or without 5 mM 3-AT and grown under 16-h light/8-h dark conditions for 24 h after stratification. The transcript levels of *EM1, EM6, DOG1, NCED9, GA3ox* and *PYL7* were examined using quantitative RT-PCR analysis. Each value is the mean ± SE of three independent experiments

Also, in the RT-PCR analyses of the expression of *EM1* and *EM6* in *abi5-1*, we showed that their expression were down-regulated due to the disruption of *ABI5* (Fig. 9).

### 3-AT treatment alters expressions of genes correlative to seed germination in Col-0, ABI5OE-Myc and *abi5-1* seeds

By analyzing RNA-Seq data, we found that many vital genes involved in seed germination expressed differentially between Col-0 and *abi5-1* mutant (Table 2). To exploring the effects of 3-AT on gene expressions, we analyzed the transcript levels of *EM1, EM6, DOG1, GA3ox, NCED9* and *PYL7* using quantitative RT-PCR analysis in the seeds of Col-0, ABI5OE-Myc and *abi5-1* which were treated with or without 5 mM 3-AT. The results of RT-PCR showed the same change trend of these genes as that shown in RNA-Seq data without 3-AT treatment in Col-0 and *abi5-1* (Fig. 9). This was also the important evidence of reliability about the RNA-Seq data.

*EM1* and *EM6* are ABA-responsive genes, which function in seed development (Gaubier et al. 1993; Carles et al. 2002). DOG1 (DELAY OF GERMINATION 1) was identified as a major regulator of dormancy in various species (Bentsink et al. 2006; Graeber et al. 2010). Here, we found that the change in *ABI5* expression had effects on these gene expressions, especially in *abi5-1* seeds. When treated by 5 mM 3-AT, *EM1* and *EM6*, which functions in promoting seed germination, were dominantly down-regulated in Col-0, which could partly explain the inhibition of 3-AT on seed germination of Col-0. Contrarily, *DOG1*, which functions in delaying seed germination, was up-regulated in Col-0 and *abi5-1*, while down-regulated in ABI5OE-Myc under 3-AT treatment (Fig. 9). The different effects of 3-AT on gene expressions in Col-0, ABI5OE-Myc and *abi5-1* could be responsible for their different phenotypes during seed germination.

In addition, we found that 3-AT can affect the expression of genes involved ABA and GA metabolism differently in Col-0, ABI5OE-Myc and *abi5-1*. Genetic and physiological evidence supports the importance of two hormones, abscisic acid (ABA) and gibberellins (GAs), in seed dormancy and germination (Koornneef et al. 2009; Finkelstein et al. 2000). NCED9 regulates a key step of ABA biosynthesis (Lefebvre et al. 2006), while GA3ox is an vital enzyme in GA biosynthesis (Yamaguchi 2008). Without 3-AT treatment, *NCED9* and *GA3ox* showed up-regulated and down-regulated in *abi5-1*, respectively. Combined with the expression of *EM1, EM6* and *DOG1*, their expression changes tended to inhibit seed germination of *abi5-1* in the presence of 3-AT treatment. However, there was no difference on the seed germination of Col-0, ABI5OE-Myc and *abi5-1* without 3-AT treatment. It seemed that there was delicate and complicated balance among genes correlative to seed germination. Due to 3-AT treatment, the balance was disrupted, which resulted in different 3-AT phenotypes of Col-0, ABI5OE-Myc and *abi5-1*.

PYL7 is a member of ABA receptors (Park et al. 2009).The change in *ABI5* expression resulted in increasing of *PYL7* expression in Col-0, ABI5OE-Myc and *abi5-1* under 3-AT treatment (Fig. 9). This suggested that 3-AT can affect seed germination mediated by ABI5 partly depending on ABA signaling.

## Discussion

It is well known that ABI5 is a positive regulator in ABA signaling (Finkelstein 1994a, b). Lots of evidence shows that ABI5 regulates seed germination by regulating ABA signaling or the expression of genes involved in seed development. For instance, it binds to *EMs* promoter and affects their expression (Carles et al. 2002). Here we found that 3-AT, a kind of catalase activity inhibitor, could affect seed germination mediated by ABI5. In the absence of 3-AT, seed germination of *abi5* mutants and ABI5OE lines showed the same germination rates as that of Col-0. In the presence of 3-AT, seed germination of *abi5-1* and *abi5-7* showed strong 3-AT-hypersensitive phenotypes compared with wild-type seeds. Meanwhile, the seeds of ABI5OE lines, including ABI5OE-Myc and ABI5OE-GFP, showed a significantly 3-AT-insensitive phenotype (Fig. 1). Whether is ROS involved in seed germination mediated by ABI5? We observed the phenotypes of Col-0, *abi5* mutants and ABI5OE lines on the MS medium with different concentrations of H_2_O_2_ during seed germination. The results showed that *abi5* mutants were hypersensitive to H_2_O_2,_ while ABI5OE lines were insensitive, compared with Col-0 (Fig. 2), which was the direct evidence on that ROS was involved in seed germination mediated by ABI5. 3-AT, which is a CAT activity inhibitor, can affect ROS homeostasis by promoting H_2_O_2_ accumulation in the cell. The seed germination phenotypes of *abi5* mutants and ABI5OE lines in the medium containing different concentrations of exogenous H_2_O_2_ was similar to that in different concentrations of 3-AT medium, which further confirmed that ABI5 regulated seed germination partly depending on the ROS signaling pathways.

CAT members are the vital enzymes scavenging H_2_O_2_, but their functions in seed germination have not been clearly identified in *Arabidopsis*. Previous results suggested that the *CAT1* gene is mainly expressed in pollen and seeds, *CAT2* in photosynthetic tissues but also in roots and seeds, while *CAT3* is associated with vascular tissues but also leaves (Du et al. 2008). This means that they will function in different tissues and development process. The further evidence showed that different CAT members may play different roles during the same process. Previous studies indicated that ABA has an opposite effect on the transcription of *CAT1* and *CAT2* in barley aleurone, which suggests that they play the opposing roles (Fath et al. 2001). In our study, *CAT* mutants differed in their 3-AT phenotypes with regard to seed germination. Phenotypes of *CAT1* and *CAT2* mutants were completely opposite to those of *CAT3* mutants (Fig. 3d). By examining the RNA and protein expression of three CAT members, we found that there was a difference at RNA and protein level in different *CAT* mutants without 3-AT treatment. At the RNA level, the expression changes in any members can induce the expression changes in other members more or less (Fig. 3b). But at the protein level, in addition to the *CAT1* mutation, both *CAT2* and *CAT3* mutations could affect the expression of CAT family obviously (Fig. 3c). There may be an unknown mechanism on the difference of CAT expression at RNA and protein level. Moreover, there is a delicate balance among the CAT members during seed germination in response to 3-AT treatment. The final status of the balance between the three *CAT* genes determines the germination phenotype under 3-AT treatment.

We examined the CAT protein and catalase activity in seeds of Col-0, *abi5-1* and ABI5OE-Myc with or without 3-AT treatment. The *abi5* mutation or ABI5 overexpression could result in the changes in CAT protein and catalase activity (Figs. 4, 5). Furthermore, 3-AT treatment could significantly affect the catalase activity differently in Col-0, *abi5-1* and ABI5OE-Myc seeds (Fig. 5c, d), but 3-AT treatment did not obviously change the CAT protein levels (Fig. 4d). We thought that the difference of CAT protein levels in Col-0, *abi5-1* and ABI5OE-Myc was a vital reason resulting in the difference of catalase activity in response to 3-AT treatment. For example, because of CAT protein accumulation in *abi5-1*, 3-AT had a more obvious inhibition on catalase activity in *abi5-1*. The balance of CAT protein levels and catalase activity decided the final H_2_O_2_ content, which should be connected with the seed germination phenotypes in Col-0, *abi5* mutants and ABI5OE lines under the 3-AT treatment. But under normal condition, the fundamental difference of H_2_O_2_ content was not enough to affect the ROS homeostasis, so there was no difference in seed germination of Col-0, *abi5* mutants and ABI5OE lines. But 3-AT treatment could disrupt ROS homeostasis, which resulted in the difference of seed germination rate in Col-0, *abi5* mutants and ABI5OE lines. So we suggested that the direct reason for different germination rate is the change in H_2_O_2_ content due to breaking the balance of CAT protein and catalase activity under the 3-AT treatment. Based on above results, it was shown that ROS played a vital role in the seed germination mediated by ABI5.

Furtherly, RNA-Seq data supported that disruption of ABI5 could alter expressions of genes participating in ROS metabolism, especially H_2_O_2_ metabolism, and ROS signaling (Table 1). Combining with RNA-Seq data, we used RT-PCR to examine the effects of 3-AT treatment on the DEGs involved in seed germination in Col-0, *abi5-1* and ABI5OE-Myc. The results showed that due to different expression of *ABI5*, 3-AT had different effects on expressions of these vital genes, which were involved in seed development, ROS metabolism, GA metabolism, GA signaling, ABA metabolism, ABA signaling and so on (Fig. 9; Table 2).

In addition, biochemical and physiological evidence supported that ABI5 could affect the balance of three CAT members by regulating the *CAT1* expression directly (Fig. 6). Previous evidence has shown that plants have evolved the complex defense systems to regulate the homeostasis of the intracellular H_2_O_2_ level, which plays a vital role during the plant evolution process (Mhamdi et al. 2010). H_2_O_2_ is a major ROS, and the balance of the CAT protein and catalase activity, which conjointly determines the H_2_O_2_ content, should be the key factor in charge of ROS homeostasis. Previous research on ABI5 regulating seed germination is focused on ABA signaling. Here we proposed that ABI5 affects seed germination partly dependent on ROS. Much evidence has shown the crosstalk of ROS signaling, abscisic acid and ethylene signaling (Bueso et al. 2007; El-Maarouf-Bouteau et al. 2015). Our data illustrated a new crosstalk among different signaling during the seed germination mediated by ABI5. Taken together, a new mechanism of ABI5 regulating the process of seed germination was demonstrated, in which CAT acted as a link between the ABI5 and ROS homeostasis. Moreover, the different roles of CAT members in mediating ROS signaling in seeds were demonstrated.

## Materials and methods

### Plant materials and growth conditions

The mutant *Arabidopsis* lines *cat1-1* (SAIL_525_C10), *cat1-3* (SALK_208924), *cat2-2* (SALK_057998), *cat2-3* (SALK_144919), *cat3-1* (SALK_092911) and *cat3-2* (SALK_088601) were obtained from the *Arabidopsis* Biological Resource Center (Columbus, OH, USA). The homozygous mutants were identified by PCR using gene-specific primers (Supplemental Table S1). The mutated *ABI5* gene in the *abi5-1* mutant (ABRC Stock Number CS8105; named *abi5-1* in this report) was transferred from its background Wassilewskija (Ws) ecotype into Col-0 ecotype by backcrossing (Liu et al. 2012). For the generation of the transgenic lines of ABI5OE-GFP, the open reading frame (ORF) sequences of *ABI5* was amplified by PCR and cloned into the binary vector pCAMBIA-1300-221 (http://www.cambia.org) with a green fluorescent protein (GFP) tag which contains the cauliflower mosaic virus 35S promoter. The specific primers used for generating the ABI5OE-GFP are listed in Supplemental Table S7. Some of plant materials used in this study were previously described: *abi5-7* and ABI5OE-Myc (Bu et al. 2009). We examined ABI5 expression in *abi5-1, abi5-7*, ABI5OE-Myc and ABI5OE-GFP by RT-PCR (Supplemental Fig. S3).

*Arabidopsis* seeds were disinfected and plated on MS medium (Phyto Technology Laboratories, USA, product No. M519) supplemented with 3% sucrose and 0.8% agar (pH 5.9), chilled for 3 days at 4 °C and transferred to a growth chamber at 80 μmol photons m^−2^ s^−1^ or to compost soil at 120 μmol photons m^−2^ s^−1^ using cool white fluorescent lamps under a 16 h-light/8 h-dark photoperiod and 60% relative humidity.

### Phenotypic analysis

Phenotypic analysis was performed as described previously (Wu et al. 2009; Shang et al. 2010). For germination assays, ~100 seeds were sterilized and plated in triplicate on MS medium (Phyto Technology Laboratories, USA, product No. M519). The medium contained 3% sucrose and 0.8% agar (pH 5.9) supplemented with or without different concentrations of 3-AT (3-amino-1,2,4-triazole; Sigma, USA, product No. A8056) or H_2_O_2_. The seeds were chilled at 4 °C for 3 days before being placed at 22 °C under light conditions (16-h light/8-h dark), and germination (emergence of radicals) was scored at the indicated times.

### Quantitative RT-PCR analysis of mRNA expression

For quantitative RT-PCR (qRT-PCR) analysis, total RNA was isolated from imbibing seeds, which were chilled for 3 days at 4 °C to stimulate germination and then placed at 22 °C under a 16-h light/8-h dark photoperiod for 24 h. Total RNA was isolated using a seed RNA Extraction Kit (BioTeke, China, product No. RP1201), treated with RNase-free DNase I (NEB, USA, M0303) at 37 °C for 30 min to degrade genomic DNA, and purified by using an RNA Purification Kit (BioTeke, China, Product No. RP1801).

RNA (2 µg) was subjected to first-strand cDNA synthesis using a kit according to the manufacturer’s instructions (Roche Applied Science, USA, product No. AS095014379012001). The primers used for qPCR are listed in Table S1 in the Supporting Information. Analysis was performed using the Bio-Rad Real-Time System CFX96TM C1000 Thermal Cycler (Bio-Rad, USA). All experiments were repeated at least three times.

The qRT-PCR analysis was performed using the SYBR Green kit (Takara, Japan). PCR conditions were as follows: 10 min of denaturation at 95 °C, followed by 45 cycles of 10 s of denaturation at 95 °C, 5 s of annealing at 55 °C and a 10 s extension at 72 °C. The mRNA levels were measured relative to the constitutive ACTIN 2/8 mRNA (Wallström et al. 2012). All the gene-specific primers for real-time PCR analysis were shown in Supplemental Table S8.

### Protein gel blot analysis

Protein extraction of *Arabidopsis* seeds was performed essentially according to previously described procedures (Wu et al. 2009; Shang et al. 2010). The seeds were frozen in liquid nitrogen, ground in a pre-chilled mortar with a pestle to a fine powder and transferred to a 1.5 mL tube. The extraction buffer consisted of 50 mM Tris–HCl (pH 7.5), 150 mM NaCl, 1 mM EDTA, 0.1% (v/v) Triton X-100, 10% (v/v) glycerol and a protein inhibitor cocktail (Roche). The extraction buffer was added to the tube (20 mL buffer/g sample) and was placed on ice for 2 h. The mixture was centrifuged for 10 min at 12,000 rpm at 4 °C to remove insoluble material, and the supernatant was transferred to a defatted column (PEXBIO, China, product No. A020208C). After centrifugation for 2 min at 12,000 rpm at 4 °C, the sample in the collection column was ready for use.

SDS–PAGE and protein gel blot analysis were performed essentially according to previously described procedures (Wu et al. 2009; Shang et al. 2010). Specific antibody against CAT was purchased from Agrisera (Stockholm, Sweden; website: http://www.agrisera.com; product No. AS09501).

### Catalase Activity Assays

Protein was extracted from imbibing seeds, which were chilled for 3 days at 4 °C to stimulate germination and then placed at 22 °C under a 16-h light/8-h dark photoperiod for 24 h with different treatment. The supernatant was used as the crude extract and catalase activity was tested with the Catalase Activity Assay Kit (Beyotime, China, product No. S0051). Protein concentration was determined using the Bradford protein assay. Crude extract (5 µL) was mixed with catalase testing buffer, and 250 mM H_2_O_2_ was used as the substrate. Reaction time was strictly controlled and stopped with addition of stop buffer. The mixture was then added into the working color solution and incubated for at least 15 min. Absorbance at 520 nm was measured and activity was calculated. Catalase activity was indicated in units/mL or units/mg. One unit of catalase activity is defined as the quantity of enzyme catalyzing the decomposition of 1 mM H_2_O_2_ per minute.

### Determination of H_2_O_2_ content

H_2_O_2_ in seeds was quantified using the Hydrogen Peroxide Assay Kit according to the manufacturer’s instructions (Beyotime, China, product No. S0038). The imbibition seeds which were chilled for 3 days at 4 °C to stimulate germination and then placed at 22 °C under a 16-h light/8-h dark photoperiod for 24 h were ground into powders. Then the seed powder was added to the tube by 20 mL lysis buffer of Hydrogen Peroxide/g sample. The samples were centrifuged at 4 °C, 12,000×*g* for 5 min to gather the supernatants for the following tests. A 50 μL volume of each supernatant was then transferred to a 96-well microtiter plate. A 100 μL volume detection solutions of Hydrogen Peroxide was added to each well and thoroughly mixed with the supernatant. After incubation at room temperature for 30 min, the plates were read using a spectrophotometer (Synergy™ H1, Biotek, USA) at a wavelength of 560 nm. The concentration of H_2_O_2_ was calculated according to standard concentration curve originated from standard solutions upon the identical experiments. All the operations were carried out on ice.

### Yeast one-hybrid assay

Yeast one-hybrid assay was performed essentially as previously described (Shang et al. 2010) with the kit provided by Clontech (Matchmaker™ One-Hybrid Library Construction & Screening Kit CATALOG No. 630304) using the Y187 yeast strain according to the manufacturer’s instructions. The related cDNAs or promoter DNAs were amplified by PCR using the primer pairs as supplemental Table 3. The promoter fragments of *CAT1* and *CAT2* were sub-cloned into the *Sm*aI/*Ml*uI sites of pHIS2 vector. ABI5 cDNA was sub-cloned into the *Eco*RI /*Bam*HI sites of pGADT7 vector. Yeast cells were co-transformed with pHIS2 bait vector that harbored promoter of target genes and pGADT7 prey vector that contained ORF of *ABI5*. As negative controls, the yeast cells were transformed with pGADT7-ABI5 vector with pHIS2-p53 (Wei et al. 2006) or pHIS2-CAT2 harboring the corresponding promoter. Transformed yeast cells were grown in the tryptophan (Trp) and leucine (Leu)-deficient SD medium (SD–Trp–Leu) to ensure that the yeast cells were successfully co-transformed, and then the yeast cells were grown on the plates of the tryptophan (Trp), leucine (Leu) and histidine (His)-deficient SD medium (SD–Trp–Leu–His) supplemented with 3-AT (Sigma) at 0, 10, 20, and 50 mM. The plates were then incubated at 30 °C for 3 days for investigations.

### Electrophoretic mobility shift assay (EMSA)

EMSA was performed essentially as previously described (Shang et al. 2010) using the recombinant His-ABI5 protein purified from *E. coli* strain BL21 (DE3) as mentioned above. ABI5 cDNA was sub-cloned into the *Sal*I/*Eco*RI sites of pMAL-c5X vector (NEB). The biotin-labeled probes used for this EMSAs were amplified by PCR. The promoter fragments used for the EMSA were amplified by PCR using the following primer pairs as supplemental Table 4. The site-specific mutations of the G-box, named box1 mutation and box2 mutation, were introduced into the *CAT1* promoter sequences by the independent PCR.

Then the labeled probes (20 fmol) were incubated with the recombinant His–ABI5 proteins (1 µg) in a binding buffer solution (25 mM HEPES, 40 mM KCl, 5 mM MgCl_2_, 1 mM DTT, 1 mM EDTA, and 8% glycerol, pH 8.0) in the presence of 1 µg/µL of poly (deoxyinosinic–deoxycytidylic) sodium salt [poly(dI-dC)] (Sigma, USA, product No. P4929) for 30 min at room temperature. The DNA–protein reaction mixtures were separated on a 5% non-denaturing polyacrylamide gel (19:1 acrylamide:bisacrylamide) in 0.25 mM Tris-borate-EDTA at 4 °C, transferred onto a nylon membrane for 1 h at 4 °C, exposed under ultra-violet light to cross-link the samples to the membrane for 2 min. All the following procedures were performed by using the Light Shift Chemiluminescent EMSA kit (Thermo Scientific, product No. 89880) according to the manufacturer’s instructions. The mutation probes were verified by sequence analysis. Competition experiments were performed using 20-fold unlabelled fragments.

### Chromatin coimmunoprecipitation (ChIP) analysis

The experiment was performed as described previously (Shang et al. 2010), using the seedlings of the 2-week-old ABI5OE-Myc transgenic plants and the wild-type (Col-0) plants for the ChIP assay. Immunoprecipitation was performed by using Rabbit anti-Myc-tag mAb-Magnetic beads (MBL, product No. M047-11). To determine quantitatively ABI5 binding to the *CAT1* promoter, real-time PCR analysis was performed according to a procedure described previously with the Actin 2 3′-untranslated region sequence as the negative control (Shang et al. 2010). The primers used for real-time PCR analysis were listed in Supplementary Table S6. The experiment was repeated for three biological repeats with similar results.

### *Cis*-activation of CAT1promoter activity by ABI5 in tobacco leaves

This assay was performed essentially according to the previously described procedures (Shang et al. 2010). ABI5 was used for the effector construct. The promoter of the *CAT1* gene was amplified by PCR using the following primer pairs as supplemental Table S5. Then the reporter construct was composed of the *CAT1* promoter linked to *LUC*. The cDNA of the *ABI5* gene was fused to pCAMBIA1300-Myc vector downstream of the CaMV 35 S promoter and Myc tag. The cDNA of *GUS* and *GFP* were fused to pCAMBIA1300-Myc vector downstream of the CaMV 35 S promoter, which was used as an internal control. The constructs were mobilized into *A. tumefaciens strain* GV3101. Bacterial suspensions were infiltrated into young but fully expanded leaves of the 7-week old *N. benthamiana* plants using a needleless syringe. It is noteworthy that the amounts of the constructs were the same among treatments and controls for each group of assay. After infiltration, plants were grown firstly under dark for 24 h and then with 16 h light/day for 60 h at room temperature. The LUC activity was observed with a CCD imaging apparatus (Andor iXon, Andor, UK).

### RNA sequencing and bioinformatics analysis of RNA-Seq data

For RNA sequencing (RNA-Seq) analysis, total RNA was isolated from imbibed seeds of wild type Col-0 and *abi5-1* plants, which had been chilled for 3 days at 4 °C to stimulate germination and then placed at 22 °C under a 16-h light/8-h dark photoperiod for 24 h. Total RNA was isolated using a seed RNA Extraction Kit (BioTeke, China, product No. RP1201), treated with RNase-free DNase I (NEB, USA, product No. M0303) at 37 °C for 30 min to degrade genomic DNA, and purified by using an RNA Purification Kit (BioTeke, China, product No. RP1801). The construction of RNA libraries, RNA sequencing and bioinformatics analysis were performed at Bionova company (Beijing, China). For each sample, three biological replicates were sequenced. The raw sequence reads have been deposited into the GEO database under accession number GSE90004.

## Electronic supplementary material

Below is the link to the electronic supplementary material.

Supplementary material 1 (DOC 4814 KB)

## Abbreviations

ABRC: Arabidopsis Biological Resource Center
CAT: Catalase
ROS: Reactive oxygen species
3-AT: 3-aminotriazol

## References

[CR1] Albertos P, Romero-Puertas MC, Tatematsu K (2015). S-nitrosylation triggers ABI5 degradation to promote seed germination and seedling growth. Nat Commun.

[CR2] Apel K, Hirt H (2004). Reactive oxygen species: metabolism, oxidative stress, and signal transduction. Annu Rev Plant Biol.

[CR3] Bailly C (2004). Active oxygen species and antioxidants in seed biology. Seed Sci Res.

[CR4] Bailly C, El-Maarouf-Bouteau H, Corbineau F (2008). From intracellular signaling networks to cell death: the dual role of reactive oxygen species in seed physiology. C R Biol.

[CR5] Bentsink L, Jowett J, Hanhart CJ (2006). Cloning of DOG1, a quantitative trait locus controlling seed dormancy in *Arabidopsis*. Proc Natl Acad Sci.

[CR6] Bu Q, Li H, Zhao Q (2009). The Arabidopsis RING finger E3 ligase RHA2a is a novel positive regulator of abscisic acid signaling during seed germination and early seedling development. Plant Physiol.

[CR7] Bueso E, Alejandro S, Carbonell P (2007). The lithium tolerance of the *Arabidopsis cat2* mutant reveals a cross-talk between oxidative stress and ethylene. Plant J.

[CR8] Carles C, Bies-Etheve N, Aspart L (2002). Regulation of *Arabidopsis thaliana* Em genes: role of ABI5. Plant J.

[CR9] Chen ZX, Silva H, Klessig DF (1993). Active oxygen species in the induction of plant systemic acquired-resistance by salicylic acid. Science.

[CR10] Chevalier C, Yamaguchi J, McCourt P (1992). Nucleotide sequence of a carrier DNA for catalase from *Arabidopsis thaliana*. Plant Physiol.

[CR11] Clark D, Durner J, Navarre DA (2000). Nitric oxide inhibition of tobacco catalase and ascorbate peroxidase. Mol Plant Microbe Interact.

[CR12] Dat J, Vandenabeele S, Vranová E (2000). Dual action of the active oxygen species during plant stress responses. Cell Mol Life Sci.

[CR13] Du YY, Wang PC, Chen J (2008). Comprehensive functional analysis of the catalase gene family in *Arabidopsis thaliana*. J Integr Plant Biol.

[CR14] Durner J, Klessig DF (1996). Salicylic acid is a modulator of tobacco and mammalian catalases. J Biol Chem.

[CR15] El-Maarouf-Bouteau H, Job C, Job D (2007). ROS signaling in seed dormancy alleviation. Plant Signal Behav.

[CR16] El-Maarouf-Bouteau H, Sajjad Y, Bazin J (2015). Reactive oxygen species, abscisic acid and ethylene interact to regulate sunflower seed germination. Plant Cell Environ.

[CR17] Fath A, Bethke PC, Jones RL (2001). Enzymes that scavenge reactive oxygen species are down-regulation prior to gibberellic acid-induced programmed cell death in barley aleurone. Plant Physiol.

[CR18] Finkelstein RR (1994). Mutations at two new Arabidopsis ABA response loci are similar to the *abi3* mutations. Plant J.

[CR19] Finkelstein RR (1994). Maternal effects govern variable dominance of two abscisic acid response mutations in *Arabidopsis thaliana*. Plant Physiol.

[CR20] Finkelstein RR, Lynch TJ (2000). The *Arabidopsis* abscisic acid response gene ABI5 encodes a basic leucine zipper transcription factor. Plant Cell.

[CR21] Fontaine O, Huault C, Pavis N (1994). Dormancy break age of *Hordeum vulgare* seeds: effects of hydrogen peroxide and scarification on glutathione level and glutathione reductase activity. Plant Physiol Biochem.

[CR22] Foyer CH, Noctor G (2000). Oxygen processing in photosynthesis: regulation and signaling. New Phytol.

[CR23] Frugoli JA, Zhong HH, Nuccio ML (1996). Catalase is encoded by a multigene family in *Arabidopsis thaliana* (L) Heynh. Plant Physiol.

[CR24] Gaubier P, Raynal M, Hull G (1993). Two different Em-like genes are expressed in *Arabidopsis thaliana* seeds during maturation. Mol Gen Genet.

[CR25] Graeber K, Linkies A, Müller K (2010). Cross-species approaches to seed dormancy and germination: conservation and biodiversity of ABA-regulated mechanisms and the Brassicaceae DOG1 genes. Plant Mol Biol.

[CR26] Guan L, Zhao J, Scandalios JG (2000). Cis-elements and transfactors that regulate expression of the maize *Cat1* antioxidant gene in response to ABA and osmotic stress: H_2_O_2_ is the likely intermediary signaling molecule for the response. Plant J.

[CR27] Hoffmann-Benning S, Kende H (1992). On the role of abscisic acid and gibberellin in the regulation of growth in rice. Plant Physiol.

[CR28] Hu YQ, Liu S, Yuan HM (2010). Functional comparison of catalase genes in the elimination of photorespiratory H_2_O_2_ using promoter- and 3′-untranslated region exchange experiments in the *Arabidopsis cat2* photorespiratory mutant. Plant Cell Environ.

[CR29] Koornneef M, Bentsink L, Hilhorst H (2009). Seed dormancy and germination. Curr Opin Plant Biol.

[CR30] Laloi C, Apel K, Danon A (2004). Reactive oxygen signaling: the latest news. Curr Opin Plant Biol.

[CR31] Langmead B, Trapnell C, Pop M (2009). Ultrafast and memory-efficient alignment of short DNA sequences to the human genome. Genome Biol.

[CR32] Lefebvre V, North H, Frey A (2006). Functional analysis of Arabidopsis NCED6 and NCED9 genes indicates that ABA synthesized in the endosperm is involved in the induction of seed dormancy. Plant J.

[CR33] Liu ZQ, Yan L, Wu Z (2012). Cooperation of three WRKY-domain transcription factors WRKY18, WRKY40 and WRKY60 in repressing two ABA-responsive genes ABI4 and ABI5 in Arabidopsis. J Exp Bot.

[CR34] Margoliash E, Novogrodsky A, Schejter A (1960). Irreversible reaction of 3-amino-1-2-4-triazole and related inhibitors with the protein of catalase. Biochem J.

[CR35] Mhamdi A, Queval G, Chaouch S (2010). Catalase function in plants: a focus on *Arabidopsis* mutants as stress-mimic models. J Exp Bot.

[CR36] Miller G, Shulaev V, Mittler R (2008). Reactive oxygen signaling and abiotic stress. Plant Physiol.

[CR37] Möller IM, Sweetlove LJ (2015). ROS signalling–specificity is required. Trends Plant Sci.

[CR38] Nambara E, Suzuki M, Abrams S (2002). A screen for genes that function in abscisic acid signaling in *Arabidopsis thaliana*. Genetics.

[CR39] Ogawa K, Iwabuchi M (2001). A mechanism for promoting the germination of *Zinnia elegans* seeds by hydrogen peroxide. Plant Cell Physiol.

[CR41] Park SY, Fung P, Nishimura N (2009). Abscisic acid inhibits type 2 C protein phosphatases via the PYR/PYL family of START proteins. Science.

[CR42] Polidoros AN, Scandalios JG (1999). Role of hydrogen peroxide and different classes of antioxidants in the regulation of catalase and glutathione S-transferase gene expression in maize (*Zea mays* L.). Plant Physiol.

[CR43] Shang Y, Yan L, Liu ZQ (2010). The Mg-chelatase H subunit of *Arabidopsis* antagonizes a group of WRKY transcription repressors to relieve ABA-responsive genes of inhibition. Plant Cell.

[CR44] Smykowski A, Zimmermann P, Zentgraf U (2010). G-box binding factor reduces *CATALASE2* expression and regulates the onset of leaf senescence in *Arabidopsis*. Plant Physiol.

[CR45] Tamura N, Yoshida T, Tanaka A (2006). Isolation and characterization of high temperature resistant germination mutants of *Arabidopsis thaliana*. Plant Cell Physiol.

[CR46] Torres MA, Dangl JL (2005). Functions of the respiratory burst oxidase in biotic interactions, abiotic stress and development. Curr Opin Plant Biol.

[CR56] Wallström Sabá V., Aidemark Mari, Escobar Matthew A., Rasmusson Allan G. (2012). An alternatively spliced domain of the NDC1 NAD(P)H dehydrogenase gene strongly influences the expression of the ACTIN2 reference gene in Arabidopsis thaliana. Plant Science.

[CR47] Wang M, van der Meulen RM, Visser K (1998). Effects of dormancy-breaking chemicals on ABA levels in barley grain embryos. Seed Sci Res.

[CR48] Wang YP, Li L, Ye TT (2013). The inhibitory effect of ABA on floral transition is mediated by ABI5 in *Arabidopsis*. J Exp Bot.

[CR49] Wei CL, Wu Q, Vega VB (2006). A global map of p53 transcription-factor binding sites in the human genome. Cell.

[CR50] Wu FQ, Xin Q, Cao Z (2009). The magnesium-chelatase H subunit binds abscisic acid and functions in abscisic acid signaling: new evidence in *Arabidopsis*. Plant Physiol.

[CR51] Yamaguchi S (2008). Gibberellin metabolism and its regulation. Annu Rev Plant Biol.

[CR52] Ye N, Zhu G, Liu Y (2012). Ascorbic acid and reactive oxygen species are involved in the inhibition of seed germination by abscisic acid in rice seeds. J Exp Bot.

[CR53] Zeevaart JAD, Creelman RA (1988). Metabolism and physiology of abscisic acid. Annu Rev Plant Physiol Plant Mol Biol.

[CR54] Zheng XM, Moriyama EN (2013). Comparative studies of differential gene calling using RNA-Seq data. BMC Bioinform.

[CR55] Zimmermann P, Heinlein C, Orendi G (2006). Senescence specific regulation of catalases in *Arabidopsis thaliana (L.)* Heynh. Plant Cell Environ.

