# Supplementary material for: Arabidopsis ABI5 plays a role in regulating ROS homeostasis by activating CATALASE 1 transcription in seed germination
Source: Plant Mol Biol. 2017 Apr 8;94(1):197–213. doi: 10.1007/s11103-017-0603-y (PMC5437177; doi:10.1007/s11103-017-0603-y)
Supplement: Supplementary file 1 — Supplementary material 1 (DOC 4814 KB) [file 11103_2017_603_MOESM1_ESM.doc]

**Supplemental Data**

**Bi C et al. *Arabidopsis* ABI5 plays a role in regulating ROS homeostasis by activating *CATALASE 1* transcription in seed germination**


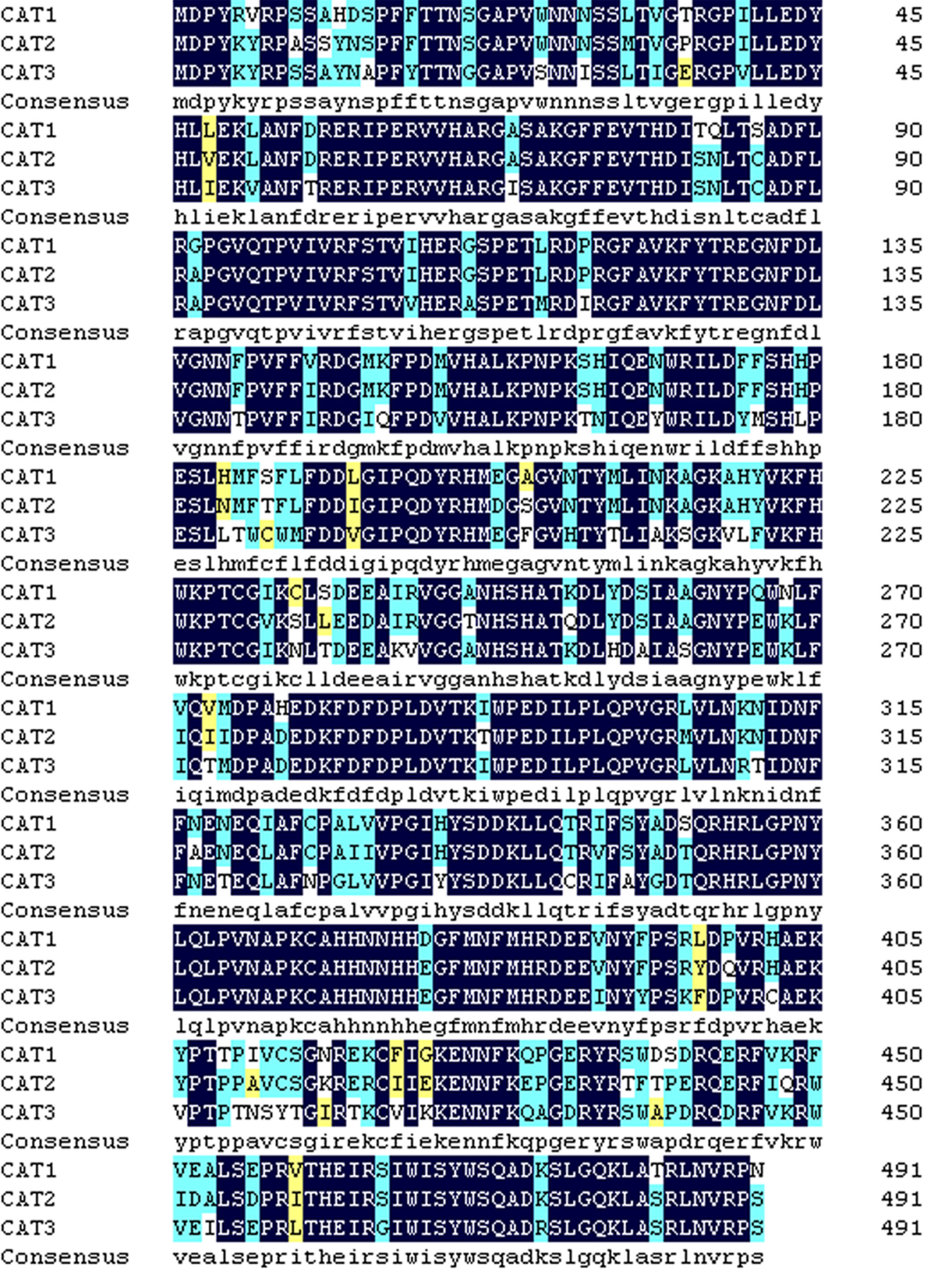


**Supplemental Fig. S1** Alignment of the amino acid sequence of CAT members in Arabidopsis.

**
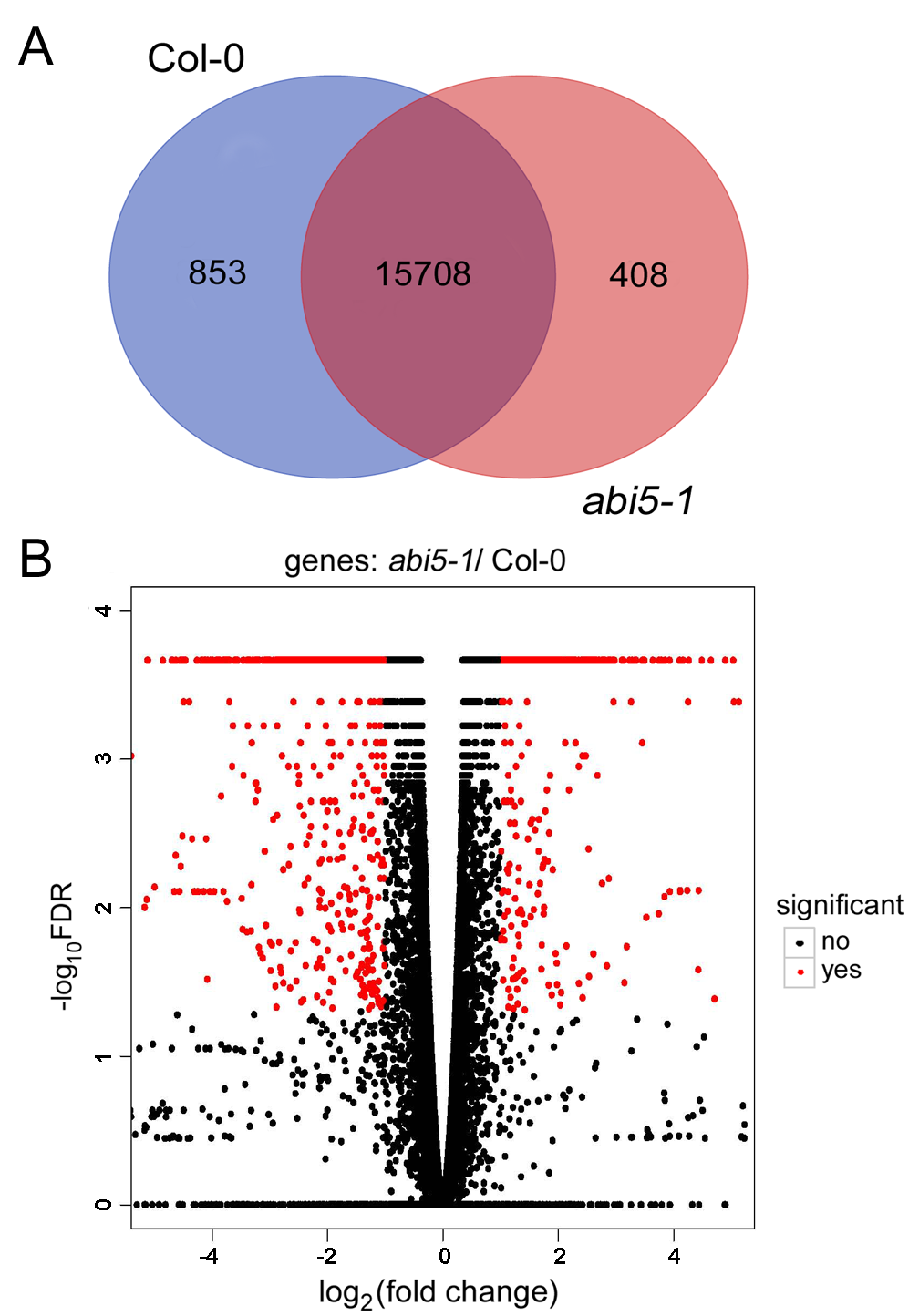
**

**Supplemental Fig. S2** RNA-seq analysis of expressed transcripts in the seeds of wild-type (Col-0) and *abi5-1*. **a** A total of 16,561 genes were found in Col-0 seeds, and of 16,116 genes in *abi5-1* seeds by RNA-seq. **b** The volcano plot map shows the number of the expressed genes up- or downregulated by Col-0 and *abi5-1* mutation. The red plot shows differentially expression genes with a cut-off value = 2-fold or 0.5-fold (FDR 0.05). The black plot shows no differentially expression genes with a cut-off value = 2-fold or 0.5-fold (FDR 0.05). When the value of log_2_ (fold change) is lower than 0, this means the gene is downregulated; on the contrary, when the value of log_2_ (fold change) is higher than 0, this means the gene is upregulated. All comparisons were made relative to the gene expression level of the wild type (Col-0).

**
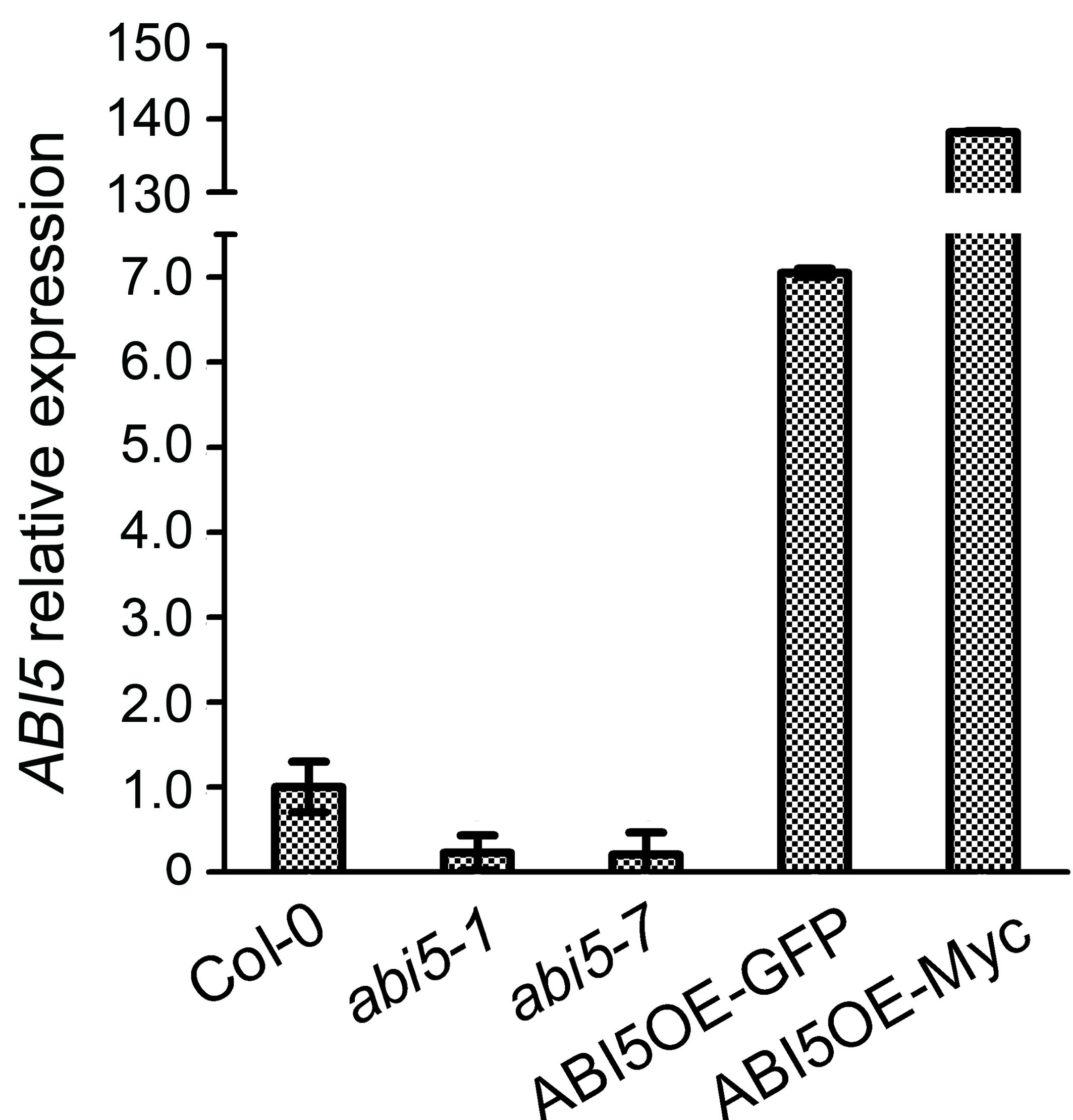
**

**Supplemental Fig. S3** RT-PCR analysis of ABI5 expression in Col-0, *abi5-1*, *abi5-7*, ABI5OE-GFP and ABI5OE-Myc. Each value is the mean ± SE of three independent experiments. The assay was independently repeated three times.


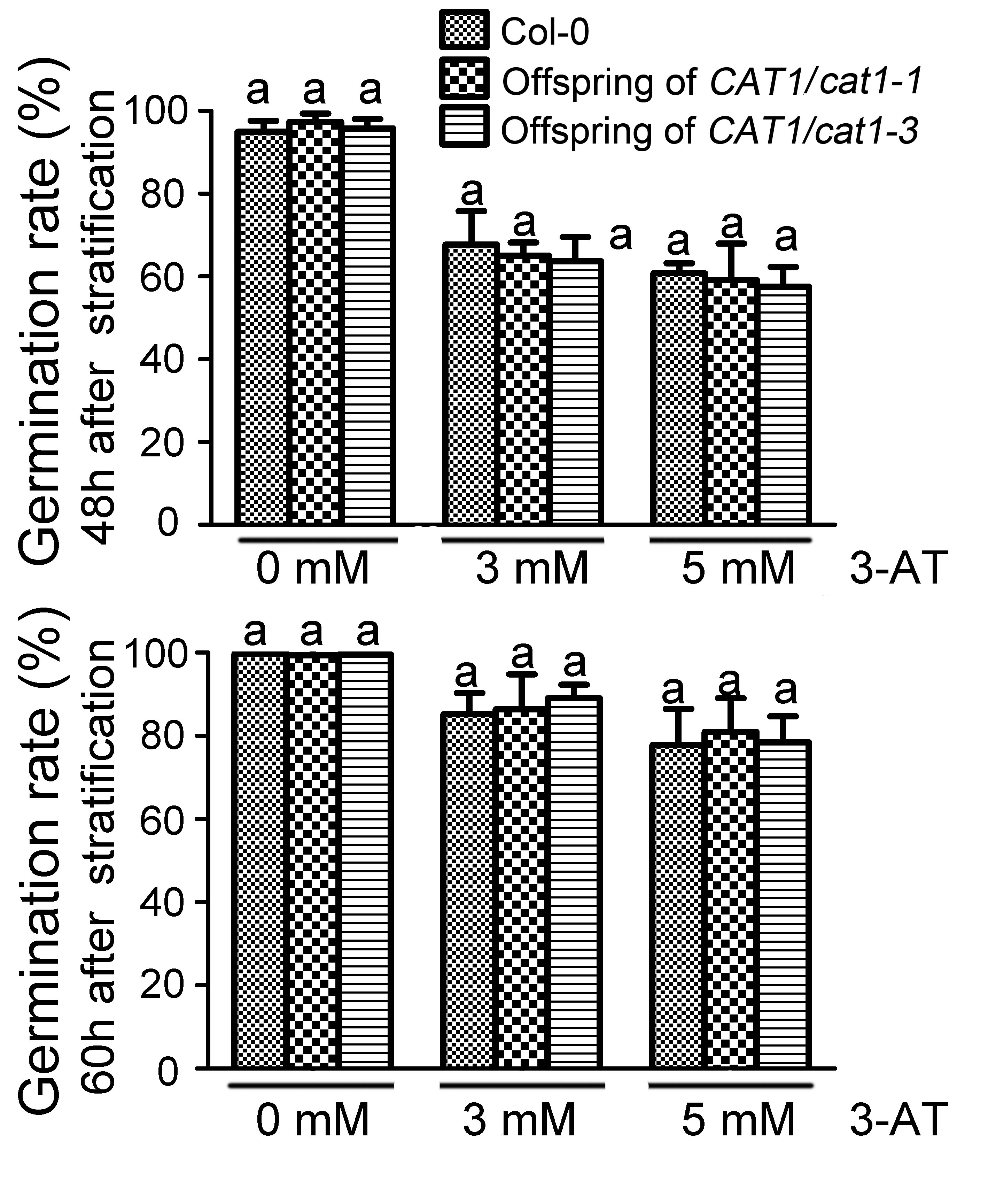


**Supplemental Fig. S4** The *cat1* phenotype on 3-AT was connected with low expression of *CAT1.*

Seeds of the wild-type (Col-0), offspring of *CAT1*/*cat1-1* and *CAT1*/*cat1-3* are grown under light conditions (16-h light/8-h dark) after stratification on MS medium supplemented with 0, 3, 5 mM 3-AT. Germination rates are recorded for 48h and 60 h. Each value is the mean ± SE of at least three independent experiments. Different letters indicate significant differences at P<0.05 (Duncan’s multiple range test) when comparing values within the same 3-AT concentration.

**Supplemental Table S1.** Primers for identification of T-DNA insertion

| *cat1-1* (SAIL_525_C10) | LP: | 5’- GTAAGAGATCCAAATGCTGCG-3’ |
| --- | --- | --- |
|  | RP: | 5’- ATTGAAACCGAATCCCAAGTC-3’ |
| *cat1-3* (SALK_208924) | LP: | 5’- ATTGAAACCGAATCCCAAGTC-3’ |
|  | RP: | 5’- CCAGAGATTTGTCTGCCTGAG-3’ |
| *cat2-2* (SALK_057998) | LP: | 5’- AGAGGCAAGATATCCTCAGGC-3’ |
|  | RP: | 5’- TCTGGTGCTCCTGTATGGAAC-3’ |
| *cat2-3* (SALK_144919) | LP: | 5’- TTGACGTTCCTGAATTTCTCG-3’ |
|  | RP: | 5’- TTCCCCTGCATACAAAGAATG-3’ |
| *cat3-1* (SALK_092911) | LP: | 5’-TAACCGGAGTTTGAACACCAG -3’ |
|  | RP: | 5’-TCGATTGTTTAGACGTCCGAC-3’ |
| *cat3-2* (SALK_088601) | LP: | 5’- AATAATGGATTCCCGAAGTGG-3’ |
|  | RP: | 5’- GACCCCAAGTTACCCTCAATC-3’ |

**Supplemental Table S2.** Primers for analysis of T-DNA flanking sequence

| LBa1: | 5’-GGTTCACGTAGTGGGCCATC-3’ |
| --- | --- |
| RBa1: | 5’-GTTTCTGACGTATGTGCTTAGC-3’ |
| LB1: | 5'-GCCTTTTCAGAAATGGATAAATAGCCTTGCTTCC-3' |
| RB1: | 5’-ATTAGGCACCCCAGGCTTTACACTTTATG-3’ |

**Supplemental Table S3. Primers for analysis of protein interaction by Yeast One-Hybrid System**

| *CAT1* promoter | Forward primer: | 5’- ATACCCGGGAGGCCAATCTCCATATAAGC -3’ |
| --- | --- | --- |
|  | Reverse primer: | 5’- CACACGCGTGATGCTTGAAGACAATTTTGAT-3’ |
| *CAT2* promoter | Forward primer: | 5’- ATACCCGGGGGCAATTCAGTACCATGTATATG -3’ |
|  | Reverse primer: | 5’- CACACGCGTGGTTTGATGAGAAGAGAGCTT-3’ |
| *ABI5* cDNA | Forward primer: | 5’- CCGGAATTCATGGTAACTAGAGAAACGAA -3’ |
|  | Reverse primer: | 5’- CGCGGATCCTTAGAGTGGACAACTCGGGT -3’ |

**Supplemental Table S4.** Primers for electrophoretic mobility shift assay (EMSA) *CAT1* promoter fragment probe amplified

| Forward primer: | 5’- TCCTGAAATGGATCAACAAT -3’ |
| --- | --- |
| Reverse primer: | 5’- TTTATAGCGAGAGCCAAAAT -3’ |

**Supplemental Table S5.** Primers for luciferase complementation imaging assay (LCI) in *vivo*

| *CAT1-*LUC | Forward primer: | 5’- CCCAAGCTT GGAAACTTTGATCTTGTTGG -3’ |
| --- | --- | --- |
|  | Reverse primer: | 5’- GACTAGTGATGCTTGAAGACAATTTTG -3’ |

**Supplemental Table S6.** Primers for Chromatin co-immunoprecipitation (ChIP) in *vivo*

| p*CAT1* | Forward primer: | 5’- TCCTGAAATGGATCAACAAT -3’ |
| --- | --- | --- |
|  | Reverse primer: | 5’- TTTATAGCGAGAGCCAAAAT -3’ |

**Supplemental Table S7.** Primers for generating the transgenic lines ABI5OE-GFP

| ABI5OE-GFP | Forward primer: | 5’- CGGGATCCATGGTAACTAGAGAAACGAA -3’ |
| --- | --- | --- |
|  | Reverse primer: | 5’- GGGGTACCGAGTGGACAACTCGGGTTCC -3’ |

**Supplemental Table S8.** Gene-specific primers for real-time PCR analysis

| *ACTIN2/8* | Forward primer: | 5’- GGTAACATTGTGCTCAGTGGTGG -3’ |
| --- | --- | --- |
|  | Reverse primer: | 5’- AACGACCTTAATCTTCATGCTGC -3’ |
| *CAT1* | Forward primer: | 5’-AGGAGCCAATCACAGCC -3’ |
|  | Reverse primer: | 5’-TCAAGACCAAGCGACCA -3’ |
| *CAT2* | Forward primer: | 5’-AACTCCGCCTGCTGTCTG -3’ |
|  | Reverse primer: | 5’-ATAGGGCATCAATCCATC -3’ |
| *CAT3* | Forward primer: | 5’-TCACAGCCACGCCACTAA -3’ |
|  | Reverse primer: | 5’-AGAACCAAGCGACCAACC -3’ |
| *EM1* | Forward primer: | 5’- CAAAGCAACTGAGCAGAGAAGAGC -3’ |
|  | Reverse primer: | 5’- CCTCCCTTGCTCCTTCCTTCA-3’ |
| *EM6* | Forward primer: | 5’- CAGCAGATGGGACGCAAAGG -3’ |
|  | Reverse primer: | 5’- TATTACATCCGTGTGGGGAAGTTTG -3’ |
| *DOG1* | Forward primer: | 5’- GCTCTAATTTGGATGGGTGGT -3’ |
|  | Reverse primer: | 5’- GTCGATGTTGCGGAGAAACT -3’ |
| *NCED9* | Forward primer: | 5’- TCCCCTGCTATGTTTCTTCC -3’ |
|  | Reverse primer: | 5’- AGACGGTGGTTTGAATGTCG -3’ |
| *GA3ox* | Forward primer: | 5’- TTGGGGTCAGCGAAGAAGA -3’ |
|  | Reverse primer: | 5’- CAGAATGGTTAGGAGGGTGGA -3’ |
| *PYL7* | Forward primer: | 5’- AACACATCCTCGGTATCAAC -3’ |
|  | Reverse primer: | 5’- TCCACAACAAAAGACTCCAT -3’ |

**Supplemental Table S9.** The genes whose expression is down-regulated in *abi5-1* seeds.

| **Gene_ID** | **Fold change (*abi5-1*/Col-0)** | **P-value** | **FDR** | **Gene description** |
| --- | --- | --- | --- | --- |
|
| AT5G51620 | 1.96472E-05 | 5.00E-05 | 0.000216 | Uncharacterised protein family (UPF0172) |
| AT4G36515 | 2.6266E-05 | 5.00E-05 | 0.000216 | NA |
| AT3G48130 | 3.04431E-05 | 5.00E-05 | 0.000216 | NA |
| AT2G15128 | 3.1378E-05 | 5.00E-05 | 0.000216 | other RNA |
| AT3G43740 | 3.24738E-05 | 5.00E-05 | 0.000216 | Leucine-rich repeat (LRR) family protein |
| AT5G56795 | 3.53777E-05 | 5.00E-05 | 0.000216 | metallothionein 1B |
| AT2G26740 | 3.60497E-05 | 5.00E-05 | 0.000216 | soluble epoxide hydrolase |
| AT1G15885 | 3.61872E-05 | 5.00E-05 | 0.000216 | NA |
| AT3G29644 | 3.62854E-05 | 5.00E-05 | 0.000216 | other RNA |
| AT3G44798 | 3.86617E-05 | 5.00E-05 | 0.000216 | other RNA |
| AT3G47680 | 4.10302E-05 | 5.00E-05 | 0.000216 | DNA binding |
| AT4G01593 | 4.29091E-05 | 5.00E-05 | 0.000216 | other RNA |
| AT2G15050 | 5.18175E-05 | 5.00E-05 | 0.000216 | lipid transfer protein |
| AT2G09795 | 5.39928E-05 | 5.00E-05 | 0.000216 | other RNA |
| AT5G56380 | 5.90598E-05 | 5.00E-05 | 0.000216 | F-box/RNI-like/FBD-like domains-containing protein |
| AT1G45165 | 6.53582E-05 | 5.00E-05 | 0.000216 | Expressed protein |
| AT4G19500 | 7.25037E-05 | 5.00E-05 | 0.000216 | nucleoside-triphosphatases;transmembrane receptors;nucleotide binding;ATP binding |
| AT1G11280 | 7.48212E-05 | 5.00E-05 | 0.000216 | S-locus lectin protein kinase family protein |
| AT1G52347 | 7.74647E-05 | 5.00E-05 | 0.000216 | other RNA |
| AT4G18620 | 8.12704E-05 | 5.00E-05 | 0.000216 | PYR1-like 13 |
| AT5G16980 | 8.9226E-05 | 5.00E-05 | 0.000216 | Zinc-binding dehydrogenase family protein |
| AT3G27990 | 9.23088E-05 | 5.00E-05 | 0.000216 | other RNA |
| AT3G43690 | 9.47751E-05 | 5.00E-05 | 0.000216 | transposable element gene |
| AT1G35320 | 9.89325E-05 | 5.00E-05 | 0.000216 | NA |
| AT4G08110 | 0.000100635 | 5.00E-05 | 0.000216 | transposable element gene |
| AT1G24880 | 0.000105193 | 5.00E-05 | 0.000216 | UDP-3-O-acyl N-acetylglycosamine deacetylase family protein |
| AT4G07507 | 0.000108005 | 5.00E-05 | 0.000216 | transposable element gene |
| AT3G18535 | 0.000115919 | 5.00E-05 | 0.000216 | tubulin-tyrosine ligases |
| AT3G63052 | 0.000116942 | 0.00135 | 0.004311 | NA |
| AT5G03310 | 0.000121651 | 5.00E-05 | 0.000216 | SAUR-like auxin-responsive protein family |
| AT3G47290 | 0.000121895 | 5.00E-05 | 0.000216 | phosphatidylinositol-speciwc phospholipase C8 |
| AT5G23410 | 0.000126793 | 5.00E-05 | 0.000216 | NA |
| AT5G45095 | 0.000129703 | 5.00E-05 | 0.000216 | NA |
| AT2G41440 | 0.000131286 | 5.00E-05 | 0.000216 | NA |
| AT2G10931 | 0.000133702 | 5.00E-05 | 0.000216 | NA |
| AT1G10000 | 0.000137283 | 5.00E-05 | 0.000216 | Ribonuclease H-like superfamily protein |
| AT3G47675 | 0.000138642 | 5.00E-05 | 0.000216 | NA |
| AT5G56370 | 0.000138941 | 5.00E-05 | 0.000216 | F-box/RNI-like/FBD-like domains-containing protein |
| AT3G27997 | 0.000149712 | 5.00E-05 | 0.000216 | NA |
| AT4G12870 | 0.000158821 | 5.00E-05 | 0.000216 | Gamma interferon responsive lysosomal thiol (GILT) reductase family protein |
| AT5G28913 | 0.000161471 | 5.00E-05 | 0.000216 | transposable element gene |
| AT1G35612 | 0.000162792 | 5.00E-05 | 0.000216 | transposable element gene |
| AT2G13547 | 0.000175505 | 0.00135 | 0.004311 | NA |
| AT3G18320 | 0.000179322 | 5.00E-05 | 0.000216 | F-box and associated interaction domains-containing protein |
| AT4G12990 | 0.000182292 | 5.00E-05 | 0.000216 | NA |
| AT4G12200 | 0.000191721 | 5.00E-05 | 0.000216 | transposable element gene |
| AT1G60525 | 0.000193884 | 5.00E-05 | 0.000216 | other RNA |
| AT5G28622 | 0.000196592 | 5.00E-05 | 0.000216 | transposable element gene |
| AT2G06950 | 0.000198759 | 5.00E-05 | 0.000216 | transposable element gene |
| AT3G60150 | 0.000199045 | 5.00E-05 | 0.000216 | Protein of unknown function (DUF498/DUF598) |
| AT5G28442 | 0.000214943 | 5.00E-05 | 0.000216 | NA |
| AT5G42730 | 0.000215887 | 5.00E-05 | 0.000216 | NA |
| AT1G58400 | 0.000215924 | 5.00E-05 | 0.000216 | Disease resistance protein (CC-NBS-LRR class) family |
| AT5G46510 | 0.000224903 | 5.00E-05 | 0.000216 | Disease resistance protein (TIR-NBS-LRR class) family |
| AT5G44569 | 0.000226876 | 5.00E-05 | 0.000216 | NA |
| AT1G02220 | 0.000241617 | 5.00E-05 | 0.000216 | NAC domain containing protein 3 |
| AT4G34881 | 0.000248291 | 0.04575 | 0.088714 | NA |
| AT1G23940 | 0.000266029 | 5.00E-05 | 0.000216 | ARM repeat superfamily protein |
| AT3G47350 | 0.000280984 | 5.00E-05 | 0.000216 | hydroxysteroid dehydrogenase 2 |
| AT2G26400 | 0.000281084 | 5.00E-05 | 0.000216 | acireductone dioxygenase 3 |
| AT4G36500 | 0.000287954 | 5.00E-05 | 0.000216 | NA |
| AT5G43403 | 0.000296144 | 5.00E-05 | 0.000216 | other RNA |
| AT5G25020 | 0.000303732 | 5.00E-05 | 0.000216 | Protein of unknown function (DUF1336) |
| AT1G65150 | 0.00030518 | 5.00E-05 | 0.000216 | TRAF-like family protein |
| AT4G39925 | 0.000308246 | 5.00E-05 | 0.000216 | AT hook motif DNA-binding family protein |
| AT5G26260 | 0.000330585 | 5.00E-05 | 0.000216 | TRAF-like family protein |
| AT5G37440 | 0.000333856 | 5.00E-05 | 0.000216 | Chaperone DnaJ-domain superfamily protein |
| AT2G11140 | 0.000334318 | 5.00E-05 | 0.000216 | transposable element gene |
| AT3G29120 | 0.000339781 | 5.00E-05 | 0.000216 | transposable element gene |
| AT5G17890 | 0.000343691 | 5.00E-05 | 0.000216 | DA1-related protein 4 |
| AT2G07777 | 0.00035165 | 5.00E-05 | 0.000216 | ATP synthase 9 mitochondrial |
| AT1G60530 | 0.000362967 | 5.00E-05 | 0.000216 | Dynamin related protein 4A |
| AT3G01345 | 0.000364724 | 5.00E-05 | 0.000216 | Expressed protein |
| AT3G44420 | 0.000366265 | 5.00E-05 | 0.000216 | transposable element gene |
| AT4G36140 | 0.000385428 | 5.00E-05 | 0.000216 | disease resistance protein (TIR-NBS-LRR class), putative |
| AT4G09610 | 0.000385872 | 5.00E-05 | 0.000216 | GAST1 protein homolog 2 |
| AT5G48775 | 0.000388276 | 5.00E-05 | 0.000216 | other RNA |
| AT1G66060 | 0.000388354 | 5.00E-05 | 0.000216 | Family of unknown function (DUF577) |
| AT3G42052 | 0.000388378 | 5.00E-05 | 0.000216 | transposable element gene |
| AT3G53650 | 0.000391497 | 5.00E-05 | 0.000216 | Histone superfamily protein |
| AT3G28220 | 0.000401681 | 5.00E-05 | 0.000216 | TRAF-like family protein |
| AT5G22608 | 0.000411338 | 5.00E-05 | 0.000216 | NA |
| AT2G05185 | 0.000426638 | 5.00E-05 | 0.000216 | NA |
| AT5G26270 | 0.000440404 | 5.00E-05 | 0.000216 | NA |
| AT2G28315 | 0.000449798 | 5.00E-05 | 0.000216 | Nucleotide/sugar transporter family protein |
| AT5G51630 | 0.000474118 | 5.00E-05 | 0.000216 | Disease resistance protein (TIR-NBS-LRR class) family |
| AT1G73690 | 0.000486507 | 5.00E-05 | 0.000216 | cyclin-dependent kinase D1;1 |
| AT4G39930 | 0.000505423 | 5.00E-05 | 0.000216 | NA |
| AT5G27250 | 0.000509562 | 5.00E-05 | 0.000216 | transposable element gene |
| AT1G67626 | 0.000524519 | 5.00E-05 | 0.000216 | transposable element gene |
| AT2G11852 | 0.000526033 | 0.0386 | 0.076853 | other RNA |
| AT3G25013 | 0.000531214 | 5.00E-05 | 0.000216 | Synaptobrevin family protein |
| AT1G58848 | 0.000544232 | 5.00E-05 | 0.000216 | Disease resistance protein (CC-NBS-LRR class) family |
| AT1G75250 | 0.000546367 | 5.00E-05 | 0.000216 | RAD-like 6 |
| AT1G58590 | 0.000549638 | 5.00E-05 | 0.000216 | other RNA |
| AT5G38700 | 0.000550794 | 5.00E-05 | 0.000216 | NA |
| AT1G23201 | 0.000555586 | 5.00E-05 | 0.000216 | NA |
| AT5G28430 | 0.000556075 | 5.00E-05 | 0.000216 | transposable element gene |
| AT1G22890 | 0.000558151 | 5.00E-05 | 0.000216 | NA |
| AT3G24929 | 0.000564149 | 5.00E-05 | 0.000216 | NA |
| AT5G36930 | 0.000565713 | 5.00E-05 | 0.000216 | Disease resistance protein (TIR-NBS-LRR class) family |
| AT1G34844 | 0.000573822 | 5.00E-05 | 0.000216 | other RNA |
| AT2G12170 | 0.00057467 | 5.00E-05 | 0.000216 | NA |
| AT1G48530 | 0.000577898 | 5.00E-05 | 0.000216 | NA |
| AT5G54490 | 0.000591555 | 5.00E-05 | 0.000216 | pinoid-binding protein 1 |
| AT5G37750 | 0.000595628 | 5.00E-05 | 0.000216 | Chaperone DnaJ-domain superfamily protein |
| AT3G52748 | 0.000604018 | 5.00E-05 | 0.000216 | other RNA |
| AT5G53815 | 0.000642508 | 0.0001 | 0.000412 | transposable element gene |
| AT5G24915 | 0.000698768 | 5.00E-05 | 0.000216 | transposable element gene |
| AT4G11070 | 0.000721152 | 5.00E-05 | 0.000216 | WRKY family transcription factor |
| AT2G04050 | 0.000722455 | 5.00E-05 | 0.000216 | MATE efflux family protein |
| AT5G24660 | 0.000735191 | 5.00E-05 | 0.000216 | response to low sulfur 2 |
| AT1G10160 | 0.000829359 | 5.00E-05 | 0.000216 | transposable element gene |
| AT1G23935 | 0.00083575 | 5.00E-05 | 0.000216 | NA |
| AT4G01740 | 0.000861586 | 5.00E-05 | 0.000216 | Cysteine/Histidine-rich C1 domain family protein |
| AT2G09800 | 0.000871164 | 5.00E-05 | 0.000216 | transposable element gene |
| AT5G03350 | 0.000933768 | 5.00E-05 | 0.000216 | Legume lectin family protein |
| AT3G09160 | 0.000939426 | 5.00E-05 | 0.000216 | RNA-binding (RRM/RBD/RNP motifs) family protein |
| AT1G63880 | 0.000964041 | 5.00E-05 | 0.000216 | Disease resistance protein (TIR-NBS-LRR class) family |
| AT3G62455 | 0.000976095 | 5.00E-05 | 0.000216 | transposable element gene |
| AT5G26220 | 0.000984407 | 5.00E-05 | 0.000216 | ChaC-like family protein |
| AT3G29776 | 0.0009911 | 5.00E-05 | 0.000216 | transposable element gene |
| AT1G27030 | 0.001008489 | 5.00E-05 | 0.000216 | NA |
| AT5G48780 | 0.001024265 | 5.00E-05 | 0.000216 | disease resistance protein (TIR-NBS class) |
| AT4G08115 | 0.00106307 | 5.00E-05 | 0.000216 | transposable element gene |
| AT5G23955 | 0.001090148 | 5.00E-05 | 0.000216 | transposable element gene |
| AT2G05380 | 0.001095399 | 5.00E-05 | 0.000216 | glycine-rich protein 3 short isoform |
| AT1G21130 | 0.001169692 | 5.00E-05 | 0.000216 | O-methyltransferase family protein |
| AT5G05060 | 0.002645963 | 5.00E-05 | 0.000216 | Cystatin/monellin superfamily protein |
| AT4G10690 | 0.00395669 | 0.04575 | 0.088714 | transposable element gene |
| AT3G06433 | 0.007846299 | 5.00E-05 | 0.000216 | NA |
| AT2G24760 | 0.008186524 | 0.0323 | 0.066285 | transposable element gene |
| AT4G14980 | 0.008335191 | 0.00265 | 0.007791 | Cysteine/Histidine-rich C1 domain family protein |
| AT1G54260 | 0.011239602 | 0.04575 | 0.088714 | winged-helix DNA-binding transcription factor family protein |
| AT3G26240 | 0.016299656 | 0.00265 | 0.007791 | Cysteine/Histidine-rich C1 domain family protein |
| AT1G29410 | 0.017034176 | 0.0005 | 0.001772 | phosphoribosylanthranilate isomerase 3 |
| AT5G08600 | 0.017078636 | 0.0057 | 0.015141 | U3 ribonucleoprotein (Utp) family protein |
| AT3G28160 | 0.017332396 | 5.00E-05 | 0.000216 | transposable element gene |
| AT5G64100 | 0.018146312 | 5.00E-05 | 0.000216 | Peroxidase superfamily protein |
| AT1G12400 | 0.019159721 | 0.04575 | 0.088714 | Nucleotide excision repair, TFIIH, subunit TTDA |
| AT3G52680 | 0.01924477 | 0.00035 | 0.001288 | F-box/RNI-like/FBD-like domains-containing protein |
| AT5G54470 | 0.020682476 | 5.00E-05 | 0.000216 | B-box type zinc finger family protein |
| AT1G23950 | 0.021187582 | 5.00E-05 | 0.000216 | Protein of unknown function (DUF626) |
| AT2G21860 | 0.02271426 | 5.00E-05 | 0.000216 | violaxanthin de-epoxidase-related |
| AT2G24755 | 0.024119798 | 0.00025 | 0.000951 | other RNA |
| AT1G53887 | 0.026182355 | 0.04575 | 0.088714 | NA |
| AT3G06125 | 0.027886349 | 0.0035 | 0.009954 | other RNA |
| AT4G01700 | 0.028606672 | 0.00305 | 0.008806 | Chitinase family protein |
| AT2G41850 | 0.02882512 | 5.00E-05 | 0.000216 | polygalacturonase abscission zone A. thaliana |
| AT1G29090 | 0.028903652 | 5.00E-05 | 0.000216 | Cysteine proteinases superfamily protein |
| AT3G62460 | 0.029061948 | 5.00E-05 | 0.000216 | Putative endonuclease or glycosyl hydrolase |
| AT5G19340 | 0.031031832 | 0.0422 | 0.082944 | NA |
| AT1G53480 | 0.031477608 | 0.00245 | 0.007275 | mto 1 responding down 1 |
| AT1G35560 | 0.034874119 | 5.00E-05 | 0.000216 | TCP family transcription factor |
| AT1G35140 | 0.038898351 | 0.04575 | 0.088714 | Phosphate-responsive 1 family protein |
| AT1G73490 | 0.039040859 | 5.00E-05 | 0.000216 | RNA-binding (RRM/RBD/RNP motifs) family protein |
| AT4G16890 | 0.039081738 | 5.00E-05 | 0.000216 | disease resistance protein (TIR-NBS-LRR class), putative |
| AT1G33102 | 0.040003556 | 0.00265 | 0.007791 | NA |
| AT2G43910 | 0.040784119 | 0.0014 | 0.004449 | HARMLESS TO OZONE LAYER 1 |
| AT3G24982 | 0.040828387 | 5.00E-05 | 0.000216 | receptor like protein 40 |
| AT4G37370 | 0.041814109 | 0.02455 | 0.052565 | cytochrome P450, family 81, subfamily D, polypeptide 8 |
| AT2G44490 | 0.042609579 | 0.00265 | 0.007791 | Glycosyl hydrolase superfamily protein |
| AT5G55896 | 0.042778071 | 5.00E-05 | 0.000216 | transposable element gene |
| AT2G02100 | 0.043222987 | 5.00E-05 | 0.000216 | low-molecular-weight cysteine-rich 69 |
| AT5G52547 | 0.043226719 | 0.0017 | 0.005272 | NA |
| AT5G17090 | 0.043580647 | 5.00E-05 | 0.000216 | Cystatin/monellin superfamily protein |
| AT5G43730 | 0.044189209 | 0.001 | 0.003297 | Disease resistance protein (CC-NBS-LRR class) family |
| AT5G59370 | 0.044309423 | 5.00E-05 | 0.000216 | actin 4 |
| AT2G41100 | 0.04465521 | 0.0001 | 0.000412 | Calcium-binding EF hand family protein |
| AT3G47360 | 0.044909107 | 5.00E-05 | 0.000216 | hydroxysteroid dehydrogenase 3 |
| AT3G25010 | 0.044920599 | 5.00E-05 | 0.000216 | receptor like protein 41 |
| AT5G40890 | 0.045557373 | 5.00E-05 | 0.000216 | chloride channel A |
| AT3G16030 | 0.045935708 | 5.00E-05 | 0.000216 | lectin protein kinase family protein |
| AT2G24600 | 0.04802528 | 0.0001 | 0.000412 | Ankyrin repeat family protein |
| AT1G30935 | 0.049678065 | 0.00105 | 0.003446 | NA |
| AT5G64120 | 0.049780832 | 0.03205 | 0.065835 | Peroxidase superfamily protein |
| AT2G36120 | 0.050902439 | 0.00265 | 0.007791 | Glycine-rich protein family |
| AT5G37400 | 0.052549607 | 5.00E-05 | 0.000216 | Family of unknown function (DUF577) |
| AT3G54150 | 0.053454482 | 0.04575 | 0.088714 | S-adenosyl-L-methionine-dependent methyltransferases superfamily protein |
| AT3G05980 | 0.05354333 | 0.00265 | 0.007791 | NA |
| AT1G65370 | 0.055149243 | 5.00E-05 | 0.000216 | TRAF-like family protein |
| AT2G45180 | 0.055506048 | 5.00E-05 | 0.000216 | Bifunctional inhibitor/lipid-transfer protein/seed storage 2S albumin superfamily protein |
| AT5G23210 | 0.056058162 | 5.00E-05 | 0.000216 | serine carboxypeptidase-like 34 |
| AT5G28350 | 0.057443741 | 5.00E-05 | 0.000216 | Quinoprotein amine dehydrogenase, beta chain-like; RIC1-like guanyl-nucleotide exchange factor |
| AT4G38280 | 0.057484663 | 5.00E-05 | 0.000216 | NA |
| AT1G24530 | 0.057512141 | 0.00265 | 0.007791 | Transducin/WD40 repeat-like superfamily protein |
| AT3G47010 | 0.057861924 | 5.00E-05 | 0.000216 | Glycosyl hydrolase family protein |
| AT5G43050 | 0.058009943 | 5.00E-05 | 0.000216 | Protein of unknown function (DUF565) |
| AT5G57565 | 0.058326698 | 5.00E-05 | 0.000216 | Protein kinase superfamily protein |
| AT1G31355 | 0.058334553 | 0.04575 | 0.088714 | NA |
| AT2G21080 | 0.058825538 | 0.00105 | 0.003446 | NA |
| AT1G55450 | 0.059539637 | 0.0128 | 0.030352 | S-adenosyl-L-methionine-dependent methyltransferases superfamily protein |
| AT1G58070 | 0.059774639 | 5.00E-05 | 0.000216 | NA |
| AT2G43000 | 0.061516134 | 0.00265 | 0.007791 | NAC domain containing protein 42 |
| AT5G04530 | 0.061687507 | 0.04575 | 0.088714 | 3-ketoacyl-CoA synthase 19 |
| AT2G34080 | 0.061696146 | 5.00E-05 | 0.000216 | Cysteine proteinases superfamily protein |
| AT2G22500 | 0.063686689 | 5.00E-05 | 0.000216 | uncoupling protein 5 |
| AT1G56680 | 0.064735618 | 0.00265 | 0.007791 | Chitinase family protein |
| AT5G48620 | 0.065926474 | 5.00E-05 | 0.000216 | Disease resistance protein (CC-NBS-LRR class) family |
| AT3G26922 | 0.067600049 | 5.00E-05 | 0.000216 | F-box/RNI-like superfamily protein |
| AT5G10400 | 0.068897032 | 5.00E-05 | 0.000216 | Histone superfamily protein |
| AT4G16807 | 0.070657587 | 0.04245 | 0.083416 | NA |
| AT1G80740 | 0.070820602 | 0.0005 | 0.001772 | chromomethylase 1 |
| AT1G26680 | 0.072089401 | 5.00E-05 | 0.000216 | transcriptional factor B3 family protein |
| AT5G22600 | 0.072907497 | 0.00265 | 0.007791 | FBD / Leucine Rich Repeat domains containing protein |
| AT1G68945 | 0.073096629 | 5.00E-05 | 0.000216 | NA |
| AT5G56747 | 0.073313408 | 5.00E-05 | 0.000216 | transposable element gene |
| AT5G47510 | 0.073415681 | 5.00E-05 | 0.000216 | Sec14p-like phosphatidylinositol transfer family protein |
| AT4G13320 | 0.073474419 | 0.04615 | 0.089296 | NA |
| AT5G26800 | 0.074599562 | 5.00E-05 | 0.000216 | NA |
| AT3G21030 | 0.075324429 | 0.00315 | 0.009067 | transposable element gene |
| AT5G17870 | 0.076208465 | 5.00E-05 | 0.000216 | plastid-specific 50S ribosomal protein 6 |
| AT3G54040 | 0.076693366 | 0.04575 | 0.088714 | PAR1 protein |
| AT1G80240 | 0.077051579 | 5.00E-05 | 0.000216 | Protein of unknown function, DUF642 |
| AT1G22250 | 0.077247259 | 0.0001 | 0.000412 | NA |
| AT4G30280 | 0.077457844 | 5.00E-05 | 0.000216 | xyloglucan endotransglucosylase/hydrolase 18 |
| AT1G57990 | 0.080031992 | 0.0003 | 0.00112 | purine permease 18 |
| AT3G26480 | 0.081086338 | 0.00015 | 0.000596 | Transducin family protein / WD-40 repeat family protein |
| AT1G02205 | 0.082726532 | 5.00E-05 | 0.000216 | Fatty acid hydroxylase superfamily |
| AT1G61180 | 0.083561931 | 5.00E-05 | 0.000216 | LRR and NB-ARC domains-containing disease resistance protein |
| AT2G25330 | 0.084375014 | 0.0465 | 0.089851 | TRAF-like family protein |
| AT2G13800 | 0.085160093 | 5.00E-05 | 0.000216 | somatic embryogenesis receptor-like kinase 5 |
| AT3G46530 | 0.085401292 | 5.00E-05 | 0.000216 | NB-ARC domain-containing disease resistance protein |
| AT1G29920 | 0.089841598 | 0.0473 | 0.091118 | chlorophyll A/B-binding protein 2 |
| AT5G18860 | 0.090559544 | 0.003 | 0.008677 | inosine-uridine preferring nucleoside hydrolase family protein |
| AT4G17490 | 0.090780829 | 0.0052 | 0.014014 | ethylene responsive element binding factor 6 |
| AT1G61260 | 0.091136709 | 5.00E-05 | 0.000216 | Protein of unknown function (DUF761) |
| AT4G25810 | 0.091749006 | 0.04885 | 0.093617 | xyloglucan endotransglycosylase 6 |
| AT3G13760 | 0.091960948 | 0.00035 | 0.001288 | Cysteine/Histidine-rich C1 domain family protein |
| AT1G57860 | 0.095661663 | 5.00E-05 | 0.000216 | Translation protein SH3-like family protein |
| AT3G26170 | 0.096035453 | 0.00545 | 0.01457 | cytochrome P450, family 71, subfamily B, polypeptide 19 |
| AT2G43535 | 0.096323604 | 5.00E-05 | 0.000216 | Scorpion toxin-like knottin superfamily protein |
| AT1G28130 | 0.096840634 | 0.00015 | 0.000596 | Auxin-responsive GH3 family protein |
| AT5G39100 | 0.097179962 | 5.00E-05 | 0.000216 | germin-like protein 6 |
| AT4G13690 | 0.098054287 | 5.00E-05 | 0.000216 | NA |
| AT5G15230 | 0.098581366 | 5.00E-05 | 0.000216 | GAST1 protein homolog 4 |
| AT2G44460 | 0.100770398 | 0.00535 | 0.014339 | beta glucosidase 28 |
| AT1G45248 | 0.101094844 | 0.0002 | 0.000777 | Nucleolar histone methyltransferase-related protein |
| AT3G09940 | 0.1013028 | 0.00235 | 0.007007 | monodehydroascorbate reductase |
| AT4G18650 | 0.101976732 | 5.00E-05 | 0.000216 | transcription factor-related |
| AT5G44460 | 0.102498629 | 5.00E-05 | 0.000216 | calmodulin like 43 |
| AT3G61490 | 0.102924293 | 5.00E-05 | 0.000216 | Pectin lyase-like superfamily protein |
| AT5G52570 | 0.10420973 | 0.02445 | 0.052384 | beta-carotene hydroxylase 2 |
| AT1G62590 | 0.105759566 | 5.00E-05 | 0.000216 | pentatricopeptide (PPR) repeat-containing protein |
| AT3G47720 | 0.105802115 | 0.00055 | 0.001925 | similar to RCD one 4 |
| AT5G27890 | 0.105979893 | 5.00E-05 | 0.000216 | NA |
| AT4G21850 | 0.106021681 | 5.00E-05 | 0.000216 | methionine sulfoxide reductase B9 |
| AT2G05100 | 0.106233958 | 0.0004 | 0.001452 | photosystem II light harvesting complex gene 2.1 |
| AT1G27140 | 0.106847738 | 0.0004 | 0.001452 | glutathione S-transferase tau 14 |
| AT5G58580 | 0.107131665 | 0.04755 | 0.091506 | TOXICOS EN LEVADURA 63 |
| AT1G76680 | 0.107996813 | 5.00E-05 | 0.000216 | 12-oxophytodienoate reductase 1 |
| AT2G18193 | 0.109066336 | 0.00045 | 0.001612 | P-loop containing nucleoside triphosphate hydrolases superfamily protein |
| AT2G43590 | 0.109553768 | 5.00E-05 | 0.000216 | Chitinase family protein |
| AT5G39580 | 0.110054871 | 5.00E-05 | 0.000216 | Peroxidase superfamily protein |
| AT1G09240 | 0.11060456 | 0.0072 | 0.018505 | nicotianamine synthase 3 |
| AT3G44630 | 0.111081533 | 5.00E-05 | 0.000216 | Disease resistance protein (TIR-NBS-LRR class) family |
| AT3G02515 | 0.112575926 | 0.008 | 0.020277 | transposable element gene |
| AT5G44562 | 0.112897242 | 0.03975 | 0.078903 | other RNA |
| AT2G43150 | 0.115746409 | 5.00E-05 | 0.000216 | Proline-rich extensin-like family protein |
| AT2G20670 | 0.115883276 | 0.00875 | 0.021888 | Protein of unknown function (DUF506) |
| AT3G14362 | 0.116574977 | 0.00015 | 0.000596 | ROTUNDIFOLIA like 10 |
| AT2G10940 | 0.118755166 | 0.0013 | 0.004171 | Bifunctional inhibitor/lipid-transfer protein/seed storage 2S albumin superfamily protein |
| AT1G31430 | 0.119033255 | 5.00E-05 | 0.000216 | Pentatricopeptide repeat (PPR-like) superfamily protein |
| AT2G15830 | 0.119706186 | 5.00E-05 | 0.000216 | NA |
| AT1G25530 | 0.120321547 | 5.00E-05 | 0.000216 | Transmembrane amino acid transporter family protein |
| AT5G37630 | 0.120329566 | 5.00E-05 | 0.000216 | ARM repeat superfamily protein |
| AT2G36750 | 0.120787198 | 0.00485 | 0.01316 | UDP-glucosyl transferase 73C1 |
| AT1G67105 | 0.121375042 | 5.00E-05 | 0.000216 | other RNA |
| AT1G52905 | 0.121794851 | 5.00E-05 | 0.000216 | NA |
| AT4G13330 | 0.123306147 | 5.00E-05 | 0.000216 | S-adenosyl-L-methionine-dependent methyltransferases superfamily protein |
| AT1G69540 | 0.123636617 | 5.00E-05 | 0.000216 | AGAMOUS-like 94 |
| AT4G36600 | 0.125810089 | 5.00E-05 | 0.000216 | Late embryogenesis abundant (LEA) protein |
| AT4G37445 | 0.126055351 | 0.00655 | 0.017064 | NA |
| AT1G30380 | 0.12703701 | 0.01095 | 0.026523 | photosystem I subunit K |
| AT3G47400 | 0.129517154 | 0.0069 | 0.017847 | Plant invertase/pectin methylesterase inhibitor superfamily |
| AT1G11850 | 0.130751264 | 0.00075 | 0.002548 | NA |
| AT1G67560 | 0.131360666 | 5.00E-05 | 0.000216 | PLAT/LH2 domain-containing lipoxygenase family protein |
| AT3G47560 | 0.131894223 | 5.00E-05 | 0.000216 | alpha/beta-Hydrolases superfamily protein |
| AT2G47560 | 0.134315859 | 0.01455 | 0.033805 | RING/U-box superfamily protein |
| AT5G62630 | 0.135165426 | 5.00E-05 | 0.000216 | hipl2 protein precursor |
| AT1G66180 | 0.136447899 | 5.00E-05 | 0.000216 | Eukaryotic aspartyl protease family protein |
| AT5G35935 | 0.136720431 | 5.00E-05 | 0.000216 | transposable element gene |
| AT4G39180 | 0.136847107 | 0.02135 | 0.046712 | Sec14p-like phosphatidylinositol transfer family protein |
| AT5G53880 | 0.137117848 | 5.00E-05 | 0.000216 | NA |
| AT1G15790 | 0.13722692 | 0.0007 | 0.002394 | NA |
| AT4G30270 | 0.137579892 | 0.00015 | 0.000596 | xyloglucan endotransglucosylase/hydrolase 24 |
| AT5G14840 | 0.138175524 | 5.00E-05 | 0.000216 | NA |
| AT5G05750 | 0.138674477 | 5.00E-05 | 0.000216 | DNAJ heat shock N-terminal domain-containing protein |
| AT3G29000 | 0.140883693 | 0.00655 | 0.017064 | Calcium-binding EF-hand family protein |
| AT1G69526 | 0.141029531 | 5.00E-05 | 0.000216 | S-adenosyl-L-methionine-dependent methyltransferases superfamily protein |
| AT2G47140 | 0.141558054 | 5.00E-05 | 0.000216 | NAD(P)-binding Rossmann-fold superfamily protein |
| AT4G20420 | 0.142433527 | 0.01035 | 0.02526 | Tapetum specific protein TAP35/TAP44 |
| AT1G74880 | 0.143907747 | 5.00E-05 | 0.000216 | NAD(P)H:plastoquinone dehydrogenase complex subunit O |
| AT4G12430 | 0.14465755 | 0.01135 | 0.027367 | Haloacid dehalogenase-like hydrolase (HAD) superfamily protein |
| AT3G58850 | 0.145109206 | 5.00E-05 | 0.000216 | phy rapidly regulated 2 |
| AT5G47330 | 0.145532683 | 0.00025 | 0.000951 | alpha/beta-Hydrolases superfamily protein |
| AT1G17170 | 0.146598216 | 5.00E-05 | 0.000216 | glutathione S-transferase TAU 24 |
| AT2G30766 | 0.146727117 | 5.00E-05 | 0.000216 | NA |
| AT3G25030 | 0.147280013 | 5.00E-05 | 0.000216 | RING/U-box superfamily protein |
| AT1G10550 | 0.147853936 | 0.0018 | 0.00555 | xyloglucan:xyloglucosyl transferase 33 |
| AT1G31240 | 0.148154507 | 0.0137 | 0.032093 | Bromodomain transcription factor |
| AT5G44500 | 0.148658032 | 5.00E-05 | 0.000216 | Small nuclear ribonucleoprotein family protein |
| AT5G07580 | 0.148917955 | 5.00E-05 | 0.000216 | Integrase-type DNA-binding superfamily protein |
| AT2G43860 | 0.149194756 | 5.00E-05 | 0.000216 | Pectin lyase-like superfamily protein |
| AT3G51350 | 0.149597 | 5.00E-05 | 0.000216 | Eukaryotic aspartyl protease family protein |
| AT2G14960 | 0.151404701 | 5.00E-05 | 0.000216 | Auxin-responsive GH3 family protein |
| AT4G23270 | 0.151763063 | 5.00E-05 | 0.000216 | cysteine-rich RLK (RECEPTOR-like protein kinase) 19 |
| AT4G09600 | 0.153096091 | 5.00E-05 | 0.000216 | GAST1 protein homolog 3 |
| AT4G19030 | 0.154743489 | 5.00E-05 | 0.000216 | NOD26-like major intrinsic protein 1 |
| AT3G27610 | 0.155047211 | 5.00E-05 | 0.000216 | Nucleotidylyl transferase superfamily protein |
| AT2G43570 | 0.157221147 | 0.0003 | 0.00112 | chitinase, putative |
| AT5G66690 | 0.158191517 | 0.00165 | 0.005138 | UDP-Glycosyltransferase superfamily protein |
| AT1G69160 | 0.158293737 | 5.00E-05 | 0.000216 | NA |
| AT3G44670 | 0.158307457 | 5.00E-05 | 0.000216 | Disease resistance protein (TIR-NBS-LRR class) family |
| AT3G44480 | 0.158567247 | 5.00E-05 | 0.000216 | Disease resistance protein (TIR-NBS-LRR class) family |
| AT1G15550 | 0.158893288 | 5.00E-05 | 0.000216 | gibberellin 3-oxidase 1 |
| AT1G53490 | 0.158920581 | 5.00E-05 | 0.000216 | RING/U-box superfamily protein |
| AT3G09790 | 0.160455431 | 5.00E-05 | 0.000216 | ubiquitin 8 |
| AT5G19110 | 0.160961475 | 0.015 | 0.03468 | Eukaryotic aspartyl protease family protein |
| AT3G27900 | 0.161123768 | 0.0012 | 0.003878 | Protein of unknown function (DUF1184) |
| AT2G13665 | 0.161778068 | 5.00E-05 | 0.000216 | other RNA |
| AT3G61198 | 0.161784414 | 0.00765 | 0.019515 | other RNA |
| AT5G59680 | 0.162427511 | 5.00E-05 | 0.000216 | Leucine-rich repeat protein kinase family protein |
| AT1G47395 | 0.162690192 | 5.00E-05 | 0.000216 | NA |
| AT3G25020 | 0.163285665 | 5.00E-05 | 0.000216 | receptor like protein 42 |
| AT5G48850 | 0.164688609 | 5.00E-05 | 0.000216 | Tetratricopeptide repeat (TPR)-like superfamily protein |
| AT3G27360 | 0.164895787 | 5.00E-05 | 0.000216 | Histone superfamily protein |
| AT1G07610 | 0.165524736 | 5.00E-05 | 0.000216 | metallothionein 1C |
| AT2G21830 | 0.166210983 | 5.00E-05 | 0.000216 | Cysteine/Histidine-rich C1 domain family protein |
| AT1G52825 | 0.166840554 | 0.0001 | 0.000412 | NA |
| AT1G12160 | 0.167610638 | 5.00E-05 | 0.000216 | Flavin-binding monooxygenase family protein |
| AT2G23600 | 0.167610717 | 5.00E-05 | 0.000216 | acetone-cyanohydrin lyase |
| AT4G22570 | 0.168337346 | 5.00E-05 | 0.000216 | adenine phosphoribosyl transferase 3 |
| AT3G51810 | 0.169401073 | 5.00E-05 | 0.000216 | Stress induced protein |
| AT1G17180 | 0.170603877 | 5.00E-05 | 0.000216 | glutathione S-transferase TAU 25 |
| AT4G38410 | 0.171315669 | 5.00E-05 | 0.000216 | Dehydrin family protein |
| AT5G57785 | 0.171389993 | 0.0066 | 0.017176 | NA |
| AT3G56060 | 0.171913778 | 5.00E-05 | 0.000216 | Glucose-methanol-choline (GMC) oxidoreductase family protein |
| AT1G66480 | 0.172202107 | 5.00E-05 | 0.000216 | plastid movement impaired 2 |
| AT5G59720 | 0.173007385 | 0.01325 | 0.031215 | heat shock protein 18.2 |
| AT1G27130 | 0.17384023 | 5.00E-05 | 0.000216 | glutathione S-transferase tau 13 |
| AT3G49940 | 0.173919607 | 0.0003 | 0.00112 | LOB domain-containing protein 38 |
| AT1G11580 | 0.174009458 | 5.00E-05 | 0.000216 | methylesterase PCR A |
| AT5G39800 | 0.174507069 | 5.00E-05 | 0.000216 | Mitochondrial ribosomal protein L27 |
| AT3G02910 | 0.175785041 | 5.00E-05 | 0.000216 | AIG2-like (avirulence induced gene) family protein |
| AT5G18270 | 0.17744639 | 5.00E-05 | 0.000216 | Arabidopsis NAC domain containing protein 87 |
| AT1G44575 | 0.17757334 | 5.00E-05 | 0.000216 | Chlorophyll A-B binding family protein |
| AT3G52561 | 0.177755015 | 0.0004 | 0.001452 | NA |
| AT5G54740 | 0.17822953 | 5.00E-05 | 0.000216 | seed storage albumin 5 |
| AT5G63020 | 0.178514045 | 0.00035 | 0.001288 | Disease resistance protein (CC-NBS-LRR class) family |
| AT1G22220 | 0.179260815 | 5.00E-05 | 0.000216 | F-box family protein |
| AT3G26200 | 0.179504862 | 0.00365 | 0.010317 | cytochrome P450, family 71, subfamily B, polypeptide 22 |
| AT1G30700 | 0.180335551 | 0.0006 | 0.002077 | FAD-binding Berberine family protein |
| AT1G31600 | 0.180770458 | 5.00E-05 | 0.000216 | RNA-binding (RRM/RBD/RNP motifs) family protein |
| AT2G33220 | 0.180918332 | 5.00E-05 | 0.000216 | GRIM-19 protein |
| AT3G26180 | 0.181011955 | 0.00385 | 0.010775 | cytochrome P450, family 71, subfamily B, polypeptide 20 |
| AT1G66160 | 0.18184749 | 0.0002 | 0.000777 | CYS, MET, PRO, and GLY protein 1 |
| AT4G22235 | 0.182718331 | 5.00E-05 | 0.000216 | Arabidopsis defensin-like protein |
| AT5G14230 | 0.183411742 | 0.0194 | 0.043082 | NA |
| AT2G39700 | 0.185417487 | 5.00E-05 | 0.000216 | expansin A4 |
| AT2G33480 | 0.185639934 | 0.04005 | 0.079396 | NAC domain containing protein 41 |
| AT1G19830 | 0.18564715 | 5.00E-05 | 0.000216 | SAUR-like auxin-responsive protein family |
| AT1G09410 | 0.186065729 | 5.00E-05 | 0.000216 | pentatricopeptide (PPR) repeat-containing protein |
| AT5G55050 | 0.18619755 | 5.00E-05 | 0.000216 | GDSL-like Lipase/Acylhydrolase superfamily protein |
| AT5G05180 | 0.188111881 | 5.00E-05 | 0.000216 | NA |
| AT3G15540 | 0.188133114 | 5.00E-05 | 0.000216 | indole-3-acetic acid inducible 19 |
| AT4G13190 | 0.188348036 | 0.0187 | 0.04175 | Protein kinase superfamily protein |
| AT2G28690 | 0.188716862 | 0.0007 | 0.002394 | Protein of unknown function (DUF1635) |
| AT4G16835 | 0.189282388 | 5.00E-05 | 0.000216 | Tetratricopeptide repeat (TPR)-like superfamily protein |
| AT2G30945 | 0.190815701 | 0.00235 | 0.007007 | NA |
| AT5G18290 | 0.19110272 | 5.00E-05 | 0.000216 | Aquaporin-like superfamily protein |
| AT5G23950 | 0.191855488 | 5.00E-05 | 0.000216 | Calcium-dependent lipid-binding (CaLB domain) family protein |
| AT5G43500 | 0.192357918 | 5.00E-05 | 0.000216 | actin-related protein 9 |
| AT3G50440 | 0.192627439 | 5.00E-05 | 0.000216 | methyl esterase 10 |
| AT5G54270 | 0.19263772 | 5.00E-05 | 0.000216 | light-harvesting chlorophyll B-binding protein 3 |
| AT5G43745 | 0.192717344 | 5.00E-05 | 0.000216 | Protein of unknown function (DUF1012) |
| AT3G13275 | 0.193374652 | 0.01 | 0.024543 | NA |
| AT3G29760 | 0.193736086 | 5.00E-05 | 0.000216 | Haloacid dehalogenase-like hydrolase (HAD) superfamily protein |
| AT2G17650 | 0.193784354 | 5.00E-05 | 0.000216 | AMP-dependent synthetase and ligase family protein |
| AT5G64880 | 0.193787495 | 5.00E-05 | 0.000216 | NA |
| AT5G43270 | 0.193802925 | 5.00E-05 | 0.000216 | squamosa promoter binding protein-like 2 |
| AT5G45280 | 0.193821127 | 5.00E-05 | 0.000216 | Pectinacetylesterase family protein |
| AT3G45443 | 0.194433481 | 5.00E-05 | 0.000216 | NA |
| AT5G23170 | 0.194996149 | 0.00115 | 0.003732 | Protein kinase superfamily protein |
| AT5G58980 | 0.196191416 | 5.00E-05 | 0.000216 | Neutral/alkaline non-lysosomal ceramidase |
| AT4G21870 | 0.19674282 | 5.00E-05 | 0.000216 | HSP20-like chaperones superfamily protein |
| AT1G66520 | 0.196920962 | 5.00E-05 | 0.000216 | formyltransferase, putative |
| AT5G23060 | 0.196974015 | 5.00E-05 | 0.000216 | calcium sensing receptor |
| AT1G07135 | 0.197030283 | 5.00E-05 | 0.000216 | glycine-rich protein |
| AT3G44610 | 0.198127026 | 0.00015 | 0.000596 | Protein kinase superfamily protein |
| AT1G02260 | 0.198356948 | 5.00E-05 | 0.000216 | Divalent ion symporter |
| AT5G23480 | 0.198802881 | 5.00E-05 | 0.000216 | SWIB/MDM2 domain;Plus-3;GYF |
| AT5G56030 | 0.198993554 | 5.00E-05 | 0.000216 | heat shock protein 81-2 |
| AT1G03420 | 0.199044211 | 5.00E-05 | 0.000216 | transposable element gene |
| AT1G32540 | 0.199362706 | 5.00E-05 | 0.000216 | lsd one like 1 |
| AT5G44060 | 0.199555408 | 0.0021 | 0.006356 | NA |
| AT4G20480 | 0.200400278 | 5.00E-05 | 0.000216 | Putative endonuclease or glycosyl hydrolase |
| AT5G55930 | 0.201810439 | 0.001 | 0.003297 | oligopeptide transporter 1 |
| AT1G62570 | 0.202138365 | 5.00E-05 | 0.000216 | flavin-monooxygenase glucosinolate S-oxygenase 4 |
| AT4G13630 | 0.203167112 | 5.00E-05 | 0.000216 | Protein of unknown function, DUF593 |
| AT2G37170 | 0.203174011 | 5.00E-05 | 0.000216 | plasma membrane intrinsic protein 2 |
| AT3G44590 | 0.203483488 | 5.00E-05 | 0.000216 | 60S acidic ribosomal protein family |
| AT5G23730 | 0.203757659 | 5.00E-05 | 0.000216 | Transducin/WD40 repeat-like superfamily protein |
| AT5G37830 | 0.203972568 | 5.00E-05 | 0.000216 | oxoprolinase 1 |
| AT4G28290 | 0.204012981 | 0.02725 | 0.057366 | NA |
| AT1G52260 | 0.205450212 | 5.00E-05 | 0.000216 | PDI-like 1-5 |
| AT5G02440 | 0.205839522 | 0.00055 | 0.001925 | NA |
| AT2G24545 | 0.205914744 | 5.00E-05 | 0.000216 | other RNA |
| AT3G01860 | 0.206143039 | 0.00085 | 0.00285 | NA |
| AT2G21370 | 0.207836536 | 5.00E-05 | 0.000216 | xylulose kinase-1 |
| AT5G20700 | 0.207853645 | 5.00E-05 | 0.000216 | Protein of unknown function (DUF581) |
| AT1G43160 | 0.208286877 | 5.00E-05 | 0.000216 | related to AP2 6 |
| AT4G24415 | 0.208503071 | 5.00E-05 | 0.000216 | MIR824a; miRNA |
| AT1G55210 | 0.208784642 | 5.00E-05 | 0.000216 | Disease resistance-responsive (dirigent-like protein) family protein |
| AT5G54510 | 0.208865614 | 5.00E-05 | 0.000216 | Auxin-responsive GH3 family protein |
| AT1G11880 | 0.208894256 | 5.00E-05 | 0.000216 | transferases, transferring hexosyl groups |
| AT2G14660 | 0.209000311 | 5.00E-05 | 0.000216 | NA |
| AT1G58410 | 0.209227733 | 5.00E-05 | 0.000216 | Disease resistance protein (CC-NBS-LRR class) family |
| AT1G12390 | 0.209565244 | 5.00E-05 | 0.000216 | Cornichon family protein |
| AT1G64405 | 0.209775397 | 5.00E-05 | 0.000216 | NA |
| AT1G56720 | 0.210268693 | 5.00E-05 | 0.000216 | Protein kinase superfamily protein |
| AT4G38400 | 0.210669813 | 0.02565 | 0.054509 | expansin-like A2 |
| AT4G39950 | 0.211536247 | 5.00E-05 | 0.000216 | cytochrome P450, family 79, subfamily B, polypeptide 2 |
| AT2G31141 | 0.211699476 | 0.0266 | 0.05622 | NA |
| AT4G04830 | 0.212609462 | 0.0003 | 0.00112 | methionine sulfoxide reductase B5 |
| AT1G30080 | 0.213087915 | 0.00625 | 0.016398 | Glycosyl hydrolase superfamily protein |
| AT4G15390 | 0.213554657 | 5.00E-05 | 0.000216 | HXXXD-type acyl-transferase family protein |
| AT3G25780 | 0.214102837 | 5.00E-05 | 0.000216 | allene oxide cyclase 3 |
| AT4G38330 | 0.214349494 | 5.00E-05 | 0.000216 | Integral membrane protein hemolysin-III homolog |
| AT5G65390 | 0.214438672 | 5.00E-05 | 0.000216 | arabinogalactan protein 7 |
| AT4G18340 | 0.215292766 | 5.00E-05 | 0.000216 | Glycosyl hydrolase superfamily protein |
| AT1G01200 | 0.216036369 | 5.00E-05 | 0.000216 | RAB GTPase homolog A3 |
| AT1G02900 | 0.216501448 | 5.00E-05 | 0.000216 | rapid alkalinization factor 1 |
| AT4G31020 | 0.217718731 | 5.00E-05 | 0.000216 | alpha/beta-Hydrolases superfamily protein |
| AT4G16710 | 0.218203529 | 5.00E-05 | 0.000216 | glycosyltransferase family protein 28 |
| AT1G19080 | 0.218977036 | 5.00E-05 | 0.000216 | GINS complex protein |
| AT2G28660 | 0.219027039 | 0.0277 | 0.058205 | Chloroplast-targeted copper chaperone protein |
| AT5G56780 | 0.219372444 | 5.00E-05 | 0.000216 | effector of transcription2 |
| AT4G15380 | 0.219828022 | 5.00E-05 | 0.000216 | cytochrome P450, family 705, subfamily A, polypeptide 4 |
| AT1G04220 | 0.220122534 | 0.0103 | 0.025156 | 3-ketoacyl-CoA synthase 2 |
| AT3G46980 | 0.220313378 | 5.00E-05 | 0.000216 | phosphate transporter 4;3 |
| AT2G14880 | 0.220518224 | 5.00E-05 | 0.000216 | SWIB/MDM2 domain superfamily protein |
| AT3G12170 | 0.2210832 | 5.00E-05 | 0.000216 | Chaperone DnaJ-domain superfamily protein |
| AT4G00080 | 0.22151742 | 0.01755 | 0.039567 | Plant invertase/pectin methylesterase inhibitor superfamily protein |
| AT2G20835 | 0.222620282 | 5.00E-05 | 0.000216 | NA |
| AT1G80840 | 0.222628172 | 0.00655 | 0.017064 | WRKY DNA-binding protein 40 |
| AT2G31751 | 0.223190745 | 5.00E-05 | 0.000216 | unknown gene |
| AT5G53660 | 0.224396595 | 5.00E-05 | 0.000216 | growth-regulating factor 7 |
| AT3G09680 | 0.225007071 | 0.00965 | 0.023817 | Ribosomal protein S12/S23 family protein |
| AT3G52770 | 0.226268748 | 5.00E-05 | 0.000216 | protein binding |
| AT1G66620 | 0.226448712 | 5.00E-05 | 0.000216 | Protein with RING/U-box and TRAF-like domains |
| AT3G45730 | 0.227602647 | 0.00255 | 0.007539 | NA |
| AT5G43790 | 0.227977747 | 5.00E-05 | 0.000216 | Pentatricopeptide repeat (PPR) superfamily protein |
| AT2G22821 | 0.228711729 | 0.00035 | 0.001288 | other RNA |
| AT1G08940 | 0.228846042 | 0.0031 | 0.00894 | Phosphoglycerate mutase family protein |
| AT1G72416 | 0.229150469 | 0.0016 | 0.005004 | Chaperone DnaJ-domain superfamily protein |
| AT3G16150 | 0.229204491 | 5.00E-05 | 0.000216 | N-terminal nucleophile aminohydrolases (Ntn hydrolases) superfamily protein |
| AT2G26530 | 0.2293356 | 0.0035 | 0.009954 | Protein of unknown function (DUF1645) |
| AT4G02330 | 0.229512439 | 5.00E-05 | 0.000216 | Plant invertase/pectin methylesterase inhibitor superfamily |
| AT1G68520 | 0.229534568 | 0.0001 | 0.000412 | B-box type zinc finger protein with CCT domain |
| AT1G03820 | 0.230180195 | 0.00885 | 0.022102 | NA |
| AT5G48660 | 0.230989809 | 5.00E-05 | 0.000216 | B-cell receptor-associated protein 31-like |
| AT5G22880 | 0.231257212 | 5.00E-05 | 0.000216 | histone B2 |
| AT4G20690 | 0.231674666 | 0.03425 | 0.069516 | NA |
| AT1G52230 | 0.231729619 | 0.0001 | 0.000412 | photosystem I subunit H2 |
| AT1G80130 | 0.231969942 | 5.00E-05 | 0.000216 | Tetratricopeptide repeat (TPR)-like superfamily protein |
| AT4G23250 | 0.231978949 | 5.00E-05 | 0.000216 | kinases;protein kinases |
| AT5G08000 | 0.231979543 | 5.00E-05 | 0.000216 | glucan endo-1,3-beta-glucosidase-like protein 3 |
| AT3G43430 | 0.232203422 | 5.00E-05 | 0.000216 | RING/U-box superfamily protein |
| AT2G40435 | 0.232625002 | 5.00E-05 | 0.000216 | NA |
| AT3G48660 | 0.233914923 | 5.00E-05 | 0.000216 | Protein of unknown function (DUF 3339) |
| AT4G03050 | 0.234172855 | 5.00E-05 | 0.000216 | 2-oxoglutarate (2OG) and Fe(II)-dependent oxygenase superfamily protein |
| AT1G04540 | 0.234417724 | 0.0027 | 0.007914 | Calcium-dependent lipid-binding (CaLB domain) family protein |
| AT5G19530 | 0.234791974 | 5.00E-05 | 0.000216 | S-adenosyl-L-methionine-dependent methyltransferases superfamily protein |
| AT4G18197 | 0.234875521 | 0.0015 | 0.004727 | purine permease 7 |
| AT2G27080 | 0.236508438 | 0.02935 | 0.061129 | Late embryogenesis abundant (LEA) hydroxyproline-rich glycoprotein family |
| AT5G46800 | 0.236707106 | 5.00E-05 | 0.000216 | Mitochondrial substrate carrier family protein |
| AT3G57640 | 0.237650272 | 0.01025 | 0.025055 | Protein kinase superfamily protein |
| AT4G25200 | 0.237693519 | 5.00E-05 | 0.000216 | mitochondrion-localized small heat shock protein 23.6 |
| AT1G02300 | 0.237882053 | 0.00055 | 0.001925 | Cysteine proteinases superfamily protein |
| AT4G28170 | 0.238499425 | 0.01955 | 0.043364 | NA |
| AT5G43070 | 0.238717046 | 5.00E-05 | 0.000216 | WPP domain protein 1 |
| AT3G24770 | 0.238809055 | 5.00E-05 | 0.000216 | CLAVATA3/ESR-RELATED 41 |
| AT2G25220 | 0.239226662 | 5.00E-05 | 0.000216 | Protein kinase superfamily protein |
| AT1G17345 | 0.240867907 | 0.00055 | 0.001925 | SAUR-like auxin-responsive protein family |
| AT2G35310 | 0.24115391 | 5.00E-05 | 0.000216 | Transcriptional factor B3 family protein |
| AT4G09460 | 0.241215544 | 5.00E-05 | 0.000216 | myb domain protein 6 |
| AT5G43170 | 0.24194962 | 0.00115 | 0.003732 | zinc-finger protein 3 |
| AT1G12620 | 0.244614371 | 5.00E-05 | 0.000216 | Pentatricopeptide repeat (PPR) superfamily protein |
| AT1G59720 | 0.24466139 | 5.00E-05 | 0.000216 | Tetratricopeptide repeat (TPR)-like superfamily protein |
| AT3G27770 | 0.245235242 | 5.00E-05 | 0.000216 | NA |
| AT3G26330 | 0.245295529 | 0.00355 | 0.010072 | cytochrome P450, family 71, subfamily B, polypeptide 37 |
| AT1G35290 | 0.245555316 | 0.00085 | 0.00285 | Thioesterase superfamily protein |
| AT3G57160 | 0.246171132 | 0.00015 | 0.000596 | NA |
| AT4G26140 | 0.247570569 | 5.00E-05 | 0.000216 | beta-galactosidase 12 |
| AT4G27440 | 0.247706875 | 5.00E-05 | 0.000216 | protochlorophyllide oxidoreductase B |
| AT1G29357 | 0.248707972 | 5.00E-05 | 0.000216 | other RNA |
| AT1G01140 | 0.248807548 | 5.00E-05 | 0.000216 | CBL-interacting protein kinase 9 |
| AT5G42330 | 0.248917423 | 0.03255 | 0.066693 | NA |
| AT5G47220 | 0.250393125 | 5.00E-05 | 0.000216 | ethylene responsive element binding factor 2 |
| AT4G30450 | 0.251078212 | 5.00E-05 | 0.000216 | glycine-rich protein |
| AT2G19800 | 0.251103741 | 5.00E-05 | 0.000216 | myo-inositol oxygenase 2 |
| AT2G31730 | 0.251155518 | 0.00065 | 0.002236 | basic helix-loop-helix (bHLH) DNA-binding superfamily protein |
| AT5G55340 | 0.252377382 | 0.0076 | 0.019399 | MBOAT (membrane bound O-acyl transferase) family protein |
| AT1G28670 | 0.253466627 | 5.00E-05 | 0.000216 | GDSL-like Lipase/Acylhydrolase superfamily protein |
| AT1G33590 | 0.253512516 | 5.00E-05 | 0.000216 | Leucine-rich repeat (LRR) family protein |
| AT2G34130 | 0.254215121 | 5.00E-05 | 0.000216 | transposable element gene |
| AT2G06050 | 0.254418851 | 5.00E-05 | 0.000216 | oxophytodienoate-reductase 3 |
| AT5G66580 | 0.254802856 | 5.00E-05 | 0.000216 | NA |
| AT1G29930 | 0.254908759 | 5.00E-05 | 0.000216 | chlorophyll A/B binding protein 1 |
| AT1G65310 | 0.254941562 | 0.0049 | 0.013287 | xyloglucan endotransglucosylase/hydrolase 17 |
| AT5G49330 | 0.255019354 | 5.00E-05 | 0.000216 | myb domain protein 111 |
| AT1G09486 | 0.255183795 | 5.00E-05 | 0.000216 | NA |
| AT3G08770 | 0.255354361 | 5.00E-05 | 0.000216 | lipid transfer protein 6 |
| AT3G16520 | 0.256423239 | 5.00E-05 | 0.000216 | UDP-glucosyl transferase 88A1 |
| AT1G02470 | 0.256618583 | 0.0163 | 0.037157 | Polyketide cyclase/dehydrase and lipid transport superfamily protein |
| AT1G52650 | 0.256842105 | 0.0002 | 0.000777 | F-box/RNI-like superfamily protein |
| AT4G39130 | 0.257437597 | 0.04175 | 0.082259 | Dehydrin family protein |
| AT4G08910 | 0.258020996 | 0.00505 | 0.013644 | NA |
| AT3G51075 | 0.258138723 | 0.0015 | 0.004727 | other RNA |
| AT1G14090 | 0.259368726 | 5.00E-05 | 0.000216 | NA |
| AT5G22860 | 0.259719182 | 0.00055 | 0.001925 | Serine carboxypeptidase S28 family protein |
| AT2G26580 | 0.261463325 | 5.00E-05 | 0.000216 | plant-specific transcription factor YABBY family protein |
| AT2G28305 | 0.261707088 | 0.0003 | 0.00112 | Putative lysine decarboxylase family protein |
| AT1G47760 | 0.262266128 | 0.00535 | 0.014339 | AGAMOUS-like 102 |
| AT4G21200 | 0.262490765 | 0.0128 | 0.030352 | gibberellin 2-oxidase 8 |
| AT1G69490 | 0.262531617 | 5.00E-05 | 0.000216 | NAC-like, activated by AP3/PI |
| AT1G73160 | 0.262989917 | 0.00325 | 0.00933 | UDP-Glycosyltransferase superfamily protein |
| AT5G38360 | 0.264041321 | 5.00E-05 | 0.000216 | alpha/beta-Hydrolases superfamily protein |
| AT5G08640 | 0.265442635 | 0.0002 | 0.000777 | flavonol synthase 1 |
| AT5G22940 | 0.266017732 | 5.00E-05 | 0.000216 | FRA8 homolog |
| AT5G19170 | 0.266032994 | 5.00E-05 | 0.000216 | Protein of Unknown Function (DUF239) |
| AT5G43780 | 0.266383588 | 5.00E-05 | 0.000216 | Pseudouridine synthase/archaeosine transglycosylase-like family protein |
| AT3G02000 | 0.266866223 | 0.0217 | 0.047338 | Thioredoxin superfamily protein |
| AT4G20860 | 0.26699646 | 5.00E-05 | 0.000216 | FAD-binding Berberine family protein |
| AT4G29690 | 0.267307888 | 5.00E-05 | 0.000216 | Alkaline-phosphatase-like family protein |
| AT1G50630 | 0.267947764 | 5.00E-05 | 0.000216 | Protein of unknown function (DUF3537) |
| AT3G45970 | 0.268037396 | 0.036 | 0.072489 | expansin-like A1 |
| AT4G31530 | 0.268205616 | 5.00E-05 | 0.000216 | NAD(P)-binding Rossmann-fold superfamily protein |
| AT4G08035 | 0.268999973 | 5.00E-05 | 0.000216 | other RNA |
| AT1G43590 | 0.269899258 | 0.0134 | 0.03152 | transposable element gene |
| AT5G48540 | 0.271054874 | 5.00E-05 | 0.000216 | receptor-like protein kinase-related family protein |
| AT5G28460 | 0.271265248 | 0.00025 | 0.000951 | Pentatricopeptide repeat (PPR) superfamily protein |
| AT4G16230 | 0.271891615 | 5.00E-05 | 0.000216 | GDSL-like Lipase/Acylhydrolase superfamily protein |
| AT5G44410 | 0.27223251 | 5.00E-05 | 0.000216 | FAD-binding Berberine family protein |
| AT2G22470 | 0.272431403 | 5.00E-05 | 0.000216 | arabinogalactan protein 2 |
| AT1G20450 | 0.272610861 | 5.00E-05 | 0.000216 | Dehydrin family protein |
| AT5G02890 | 0.272863699 | 5.00E-05 | 0.000216 | HXXXD-type acyl-transferase family protein |
| AT3G58900 | 0.273137444 | 5.00E-05 | 0.000216 | F-box/RNI-like superfamily protein |
| AT2G38870 | 0.274610405 | 5.00E-05 | 0.000216 | Serine protease inhibitor, potato inhibitor I-type family protein |
| AT5G66270 | 0.27471286 | 5.00E-05 | 0.000216 | Zinc finger C-x8-C-x5-C-x3-H type family protein |
| AT3G22540 | 0.27528259 | 5.00E-05 | 0.000216 | Protein of unknown function (DUF1677) |
| AT4G30250 | 0.275504259 | 0.00055 | 0.001925 | P-loop containing nucleoside triphosphate hydrolases superfamily protein |
| AT4G02130 | 0.276287931 | 5.00E-05 | 0.000216 | galacturonosyltransferase 6 |
| AT3G04730 | 0.277412445 | 5.00E-05 | 0.000216 | indoleacetic acid-induced protein 16 |
| AT5G66440 | 0.278731408 | 5.00E-05 | 0.000216 | NA |
| AT4G33560 | 0.280149673 | 5.00E-05 | 0.000216 | Wound-responsive family protein |
| AT3G04420 | 0.280377149 | 5.00E-05 | 0.000216 | NAC domain containing protein 48 |
| AT3G59680 | 0.281453696 | 0.00065 | 0.002236 | NA |
| AT4G10340 | 0.282830764 | 5.00E-05 | 0.000216 | light harvesting complex of photosystem II 5 |
| AT4G13495 | 0.282835653 | 5.00E-05 | 0.000216 | other RNA |
| AT2G45560 | 0.282875638 | 5.00E-05 | 0.000216 | cytochrome P450, family 76, subfamily C, polypeptide 1 |
| AT4G33145 | 0.28289702 | 5.00E-05 | 0.000216 | NA |
| AT5G15310 | 0.283046416 | 0.0015 | 0.004727 | myb domain protein 16 |
| AT2G19320 | 0.283887869 | 0.00395 | 0.011004 | NA |
| AT5G46230 | 0.285202749 | 5.00E-05 | 0.000216 | Protein of unknown function, DUF538 |
| AT1G32060 | 0.285847037 | 5.00E-05 | 0.000216 | phosphoribulokinase |
| AT2G31750 | 0.286369412 | 5.00E-05 | 0.000216 | UDP-glucosyl transferase 74D1 |
| AT5G39790 | 0.286558895 | 5.00E-05 | 0.000216 | 5'-AMP-activated protein kinase-related |
| AT3G53370 | 0.287478668 | 5.00E-05 | 0.000216 | S1FA-like DNA-binding protein |
| AT3G44620 | 0.289023686 | 5.00E-05 | 0.000216 | protein tyrosine phosphatases;protein tyrosine phosphatases |
| AT1G67792 | 0.289388005 | 5.00E-05 | 0.000216 | other RNA |
| AT5G52900 | 0.289702366 | 0.00095 | 0.003145 | NA |
| AT2G05520 | 0.289748723 | 5.00E-05 | 0.000216 | glycine-rich protein 3 |
| AT5G48890 | 0.290324482 | 0.0471 | 0.090815 | C2H2-like zinc finger protein |
| AT3G15060 | 0.291189122 | 5.00E-05 | 0.000216 | RAB GTPase homolog A1G |
| AT1G64355 | 0.29141669 | 5.00E-05 | 0.000216 | NA |
| AT3G51440 | 0.291566098 | 0.00285 | 0.008296 | Calcium-dependent phosphotriesterase superfamily protein |
| AT1G61800 | 0.291862379 | 5.00E-05 | 0.000216 | glucose-6-phosphate/phosphate translocator 2 |
| AT4G23590 | 0.292416312 | 5.00E-05 | 0.000216 | Tyrosine transaminase family protein |
| AT5G44710 | 0.292480632 | 5.00E-05 | 0.000216 | NA |
| AT2G39675 | 0.293324246 | 0.00025 | 0.000951 | TAS1C; other RNA |
| AT4G09350 | 0.293495634 | 0.0089 | 0.022217 | Chaperone DnaJ-domain superfamily protein |
| AT5G21090 | 0.293568944 | 5.00E-05 | 0.000216 | Leucine-rich repeat (LRR) family protein |
| AT5G40670 | 0.293894739 | 5.00E-05 | 0.000216 | PQ-loop repeat family protein / transmembrane family protein |
| AT1G57800 | 0.294873898 | 5.00E-05 | 0.000216 | zinc finger (C3HC4-type RING finger) family protein |
| AT3G47390 | 0.295187299 | 5.00E-05 | 0.000216 | cytidine/deoxycytidylate deaminase family protein |
| AT5G57220 | 0.295716433 | 5.00E-05 | 0.000216 | cytochrome P450, family 81, subfamily F, polypeptide 2 |
| AT4G27810 | 0.296610067 | 0.00475 | 0.012932 | NA |
| AT5G43060 | 0.296737289 | 5.00E-05 | 0.000216 | Granulin repeat cysteine protease family protein |
| AT1G29418 | 0.298033023 | 5.00E-05 | 0.000216 | NA |
| AT1G34200 | 0.298093197 | 5.00E-05 | 0.000216 | Glyceraldehyde-3-phosphate dehydrogenase-like family protein |
| AT3G27350 | 0.2984992 | 5.00E-05 | 0.000216 | NA |
| AT5G21150 | 0.298685376 | 5.00E-05 | 0.000216 | Argonaute family protein |
| AT5G65310 | 0.298752616 | 5.00E-05 | 0.000216 | homeobox protein 5 |
| AT4G02810 | 0.299242908 | 0.0001 | 0.000412 | Protein of unknown function (DUF3049) |
| AT5G60060 | 0.299333438 | 0.0012 | 0.003878 | Protein of unknown function (DUF295) |
| AT1G66500 | 0.299345871 | 5.00E-05 | 0.000216 | Pre-mRNA cleavage complex II |
| AT1G71015 | 0.299542484 | 5.00E-05 | 0.000216 | NA |
| AT3G57157 | 0.299553602 | 0.00145 | 0.004592 | other RNA |
| AT3G28930 | 0.300528168 | 5.00E-05 | 0.000216 | AIG2-like (avirulence induced gene) family protein |
| AT5G49640 | 0.300549029 | 5.00E-05 | 0.000216 | NA |
| AT5G08565 | 0.300705061 | 5.00E-05 | 0.000216 | Transcription initiation Spt4-like protein |
| AT3G24515 | 0.301228125 | 5.00E-05 | 0.000216 | ubiquitin-conjugating enzyme 37 |
| AT5G02950 | 0.30124594 | 5.00E-05 | 0.000216 | Tudor/PWWP/MBT superfamily protein |
| AT4G31840 | 0.301815606 | 0.0052 | 0.014014 | early nodulin-like protein 15 |
| AT2G15400 | 0.302114492 | 5.00E-05 | 0.000216 | DNA-directed RNA polymerase family protein |
| AT4G04610 | 0.302587833 | 5.00E-05 | 0.000216 | APS reductase 1 |
| AT3G28200 | 0.302826851 | 5.00E-05 | 0.000216 | Peroxidase superfamily protein |
| AT2G23620 | 0.303241974 | 5.00E-05 | 0.000216 | methyl esterase 1 |
| AT3G17609 | 0.303413325 | 5.00E-05 | 0.000216 | HY5-homolog |
| AT1G78000 | 0.30395244 | 5.00E-05 | 0.000216 | sulfate transporter 1;2 |
| AT5G57830 | 0.304285996 | 0.02855 | 0.05977 | Protein of unknown function, DUF593 |
| AT1G61580 | 0.304380874 | 5.00E-05 | 0.000216 | R-protein L3 B |
| AT4G14890 | 0.304410605 | 5.00E-05 | 0.000216 | 2Fe-2S ferredoxin-like superfamily protein |
| AT3G25290 | 0.304695202 | 5.00E-05 | 0.000216 | Auxin-responsive family protein |
| AT3G11240 | 0.305969003 | 5.00E-05 | 0.000216 | arginine-tRNA protein transferase 2 |
| AT4G25900 | 0.306133916 | 5.00E-05 | 0.000216 | Galactose mutarotase-like superfamily protein |
| AT1G64390 | 0.306269464 | 5.00E-05 | 0.000216 | glycosyl hydrolase 9C2 |
| AT2G27385 | 0.306535844 | 5.00E-05 | 0.000216 | Pollen Ole e 1 allergen and extensin family protein |
| AT1G59850 | 0.307205163 | 0.00245 | 0.007275 | ARM repeat superfamily protein |
| AT3G09270 | 0.30800611 | 5.00E-05 | 0.000216 | glutathione S-transferase TAU 8 |
| AT5G51190 | 0.308039556 | 5.00E-05 | 0.000216 | Integrase-type DNA-binding superfamily protein |
| AT5G17470 | 0.310066739 | 0.0331 | 0.067552 | EF hand calcium-binding protein family |
| AT5G16350 | 0.310163834 | 5.00E-05 | 0.000216 | O-acyltransferase (WSD1-like) family protein |
| AT5G14360 | 0.310573551 | 5.00E-05 | 0.000216 | Ubiquitin-like superfamily protein |
| AT3G28140 | 0.310874028 | 5.00E-05 | 0.000216 | RNA ligase/cyclic nucleotide phosphodiesterase family protein |
| AT5G26160 | 0.311005602 | 5.00E-05 | 0.000216 | NA |
| AT4G15160 | 0.311289472 | 5.00E-05 | 0.000216 | Bifunctional inhibitor/lipid-transfer protein/seed storage 2S albumin superfamily protein |
| AT5G05340 | 0.312341584 | 0.011 | 0.026625 | Peroxidase superfamily protein |
| AT1G28600 | 0.312928807 | 5.00E-05 | 0.000216 | GDSL-like Lipase/Acylhydrolase superfamily protein |
| AT2G33070 | 0.313468895 | 5.00E-05 | 0.000216 | nitrile specifier protein 2 |
| AT1G78850 | 0.314466336 | 5.00E-05 | 0.000216 | D-mannose binding lectin protein with Apple-like carbohydrate-binding domain |
| AT4G14548 | 0.315310272 | 5.00E-05 | 0.000216 | other RNA |
| AT1G76560 | 0.315371654 | 5.00E-05 | 0.000216 | CP12 domain-containing protein 3 |
| AT3G62750 | 0.315526738 | 5.00E-05 | 0.000216 | beta glucosidase 8 |
| AT4G22305 | 0.316371061 | 5.00E-05 | 0.000216 | alpha/beta-Hydrolases superfamily protein |
| AT4G07825 | 0.316376759 | 5.00E-05 | 0.000216 | NA |
| AT2G14170 | 0.316428478 | 5.00E-05 | 0.000216 | aldehyde dehydrogenase 6B2 |
| AT4G12800 | 0.316463245 | 5.00E-05 | 0.000216 | photosystem I subunit l |
| AT5G11580 | 0.318008254 | 5.00E-05 | 0.000216 | Regulator of chromosome condensation (RCC1) family protein |
| AT5G45940 | 0.31822312 | 0.0025 | 0.007403 | nudix hydrolase homolog 11 |
| AT4G13710 | 0.318276987 | 5.00E-05 | 0.000216 | Pectin lyase-like superfamily protein |
| AT1G65590 | 0.31912854 | 5.00E-05 | 0.000216 | beta-hexosaminidase 3 |
| AT5G22920 | 0.319499367 | 5.00E-05 | 0.000216 | CHY-type/CTCHY-type/RING-type Zinc finger protein |
| AT2G25980 | 0.319515805 | 5.00E-05 | 0.000216 | Mannose-binding lectin superfamily protein |
| AT5G26290 | 0.319901769 | 5.00E-05 | 0.000216 | TRAF-like family protein |
| AT5G22300 | 0.319930997 | 5.00E-05 | 0.000216 | nitrilase 4 |
| AT1G23340 | 0.320184246 | 5.00E-05 | 0.000216 | Protein of Unknown Function (DUF239) |
| AT2G36780 | 0.320482813 | 5.00E-05 | 0.000216 | UDP-Glycosyltransferase superfamily protein |
| AT1G24575 | 0.320644021 | 5.00E-05 | 0.000216 | NA |
| AT3G12145 | 0.320824489 | 5.00E-05 | 0.000216 | Leucine-rich repeat (LRR) family protein |
| AT5G58600 | 0.320922327 | 5.00E-05 | 0.000216 | Plant protein of unknown function (DUF828) |
| AT5G24150 | 0.321212292 | 5.00E-05 | 0.000216 | FAD/NAD(P)-binding oxidoreductase family protein |
| AT1G22230 | 0.321703492 | 0.0037 | 0.010428 | NA |
| AT1G11330 | 0.322269163 | 5.00E-05 | 0.000216 | S-locus lectin protein kinase family protein |
| AT3G56170 | 0.322804014 | 5.00E-05 | 0.000216 | Ca-2+ dependent nuclease |
| AT5G40950 | 0.323323042 | 5.00E-05 | 0.000216 | ribosomal protein large subunit 27 |
| AT5G23940 | 0.323357112 | 5.00E-05 | 0.000216 | HXXXD-type acyl-transferase family protein |
| AT5G58900 | 0.323567414 | 5.00E-05 | 0.000216 | Homeodomain-like transcriptional regulator |
| AT5G17900 | 0.323773568 | 5.00E-05 | 0.000216 | microfibrillar-associated protein-related |
| AT1G54010 | 0.324055434 | 5.00E-05 | 0.000216 | GDSL-like Lipase/Acylhydrolase superfamily protein |
| AT4G30410 | 0.325492028 | 5.00E-05 | 0.000216 | sequence-specific DNA binding transcription factors |
| AT5G16340 | 0.325993389 | 5.00E-05 | 0.000216 | AMP-dependent synthetase and ligase family protein |
| AT4G04760 | 0.326210571 | 0.0028 | 0.008172 | Major facilitator superfamily protein |
| AT3G51600 | 0.326344001 | 5.00E-05 | 0.000216 | lipid transfer protein 5 |
| AT1G48470 | 0.326765078 | 5.00E-05 | 0.000216 | glutamine synthetase 1;5 |
| AT2G17280 | 0.327419562 | 5.00E-05 | 0.000216 | Phosphoglycerate mutase family protein |
| AT3G53710 | 0.327883688 | 5.00E-05 | 0.000216 | ARF-GAP domain 6 |
| AT5G55790 | 0.328323216 | 5.00E-05 | 0.000216 | NA |
| AT3G06778 | 0.32859309 | 0.00305 | 0.008806 | Chaperone DnaJ-domain superfamily protein |
| AT2G01913 | 0.328841094 | 0.00395 | 0.011004 | NA |
| AT1G56260 | 0.329328324 | 5.00E-05 | 0.000216 | NA |
| AT5G37890 | 0.329657294 | 5.00E-05 | 0.000216 | Protein with RING/U-box and TRAF-like domains |
| AT5G42690 | 0.329908309 | 5.00E-05 | 0.000216 | Protein of unknown function, DUF547 |
| AT5G38200 | 0.329922522 | 5.00E-05 | 0.000216 | Class I glutamine amidotransferase-like superfamily protein |
| AT1G62045 | 0.329977893 | 0.00095 | 0.003145 | NA |
| AT1G66200 | 0.330941305 | 0.0002 | 0.000777 | glutamine synthase clone F11 |
| AT4G16840 | 0.332114464 | 0.00015 | 0.000596 | NA |
| AT5G66590 | 0.33283692 | 5.00E-05 | 0.000216 | CAP (Cysteine-rich secretory proteins, Antigen 5, and Pathogenesis-related 1 protein) superfamily protein |
| AT1G61840 | 0.333189144 | 5.00E-05 | 0.000216 | Cysteine/Histidine-rich C1 domain family protein |
| AT1G11300 | 0.333521945 | 5.00E-05 | 0.000216 | protein serine/threonine kinases;protein kinases;ATP binding;sugar binding;kinases;carbohydrate binding |
| AT2G20570 | 0.333690135 | 5.00E-05 | 0.000216 | GBF's pro-rich region-interacting factor 1 |
| AT1G62520 | 0.333979719 | 0.0083 | 0.020913 | NA |
| AT3G44260 | 0.33423388 | 5.00E-05 | 0.000216 | Polynucleotidyl transferase, ribonuclease H-like superfamily protein |
| AT1G25440 | 0.334311601 | 5.00E-05 | 0.000216 | B-box type zinc finger protein with CCT domain |
| AT1G74710 | 0.334683913 | 5.00E-05 | 0.000216 | ADC synthase superfamily protein |
| AT5G43620 | 0.334745118 | 0.005 | 0.013533 | Pre-mRNA cleavage complex II |
| AT5G60360 | 0.334922533 | 5.00E-05 | 0.000216 | aleurain-like protease |
| AT2G30520 | 0.33559809 | 5.00E-05 | 0.000216 | Phototropic-responsive NPH3 family protein |
| AT1G29280 | 0.335712847 | 5.00E-05 | 0.000216 | WRKY DNA-binding protein 65 |
| AT2G40010 | 0.336375553 | 5.00E-05 | 0.000216 | Ribosomal protein L10 family protein |
| AT5G48450 | 0.336632988 | 5.00E-05 | 0.000216 | SKU5 similar 3 |
| AT2G35345 | 0.336679873 | 5.00E-05 | 0.000216 | NA |
| AT3G50970 | 0.337301802 | 5.00E-05 | 0.000216 | dehydrin family protein |
| AT5G14920 | 0.337700769 | 5.00E-05 | 0.000216 | Gibberellin-regulated family protein |
| AT5G35732 | 0.337830284 | 5.00E-05 | 0.000216 | NA |
| AT4G37390 | 0.338904613 | 5.00E-05 | 0.000216 | Auxin-responsive GH3 family protein |
| AT5G07860 | 0.339010158 | 5.00E-05 | 0.000216 | HXXXD-type acyl-transferase family protein |
| AT4G13340 | 0.339114711 | 5.00E-05 | 0.000216 | Leucine-rich repeat (LRR) family protein |
| AT3G01950 | 0.339266567 | 5.00E-05 | 0.000216 | Protein of unknown function (DUF 3339) |
| AT4G23990 | 0.339937005 | 5.00E-05 | 0.000216 | cellulose synthase like G3 |
| AT1G15820 | 0.340176985 | 0.00085 | 0.00285 | light harvesting complex photosystem II subunit 6 |
| AT5G41910 | 0.340216583 | 5.00E-05 | 0.000216 | Mediator complex, subunit Med10 |
| AT1G50055 | 0.340369634 | 5.00E-05 | 0.000216 | TAS1B; other RNA |
| AT4G15610 | 0.340754552 | 0.03465 | 0.070169 | Uncharacterised protein family (UPF0497) |
| AT1G62085 | 0.340861154 | 5.00E-05 | 0.000216 | Mitochondrial transcription termination factor family protein |
| AT1G74210 | 0.341100179 | 5.00E-05 | 0.000216 | PLC-like phosphodiesterases superfamily protein |
| AT1G26150 | 0.341412759 | 5.00E-05 | 0.000216 | proline-rich extensin-like receptor kinase 10 |
| AT1G34270 | 0.341585246 | 5.00E-05 | 0.000216 | Exostosin family protein |
| AT3G46030 | 0.342241922 | 5.00E-05 | 0.000216 | Histone superfamily protein |
| AT3G29770 | 0.343033151 | 0.0005 | 0.001772 | methyl esterase 11 |
| AT3G02620 | 0.343357506 | 5.00E-05 | 0.000216 | Plant stearoyl-acyl-carrier-protein desaturase family protein |
| AT1G68238 | 0.343516087 | 5.00E-05 | 0.000216 | NA |
| AT1G63855 | 0.344098229 | 5.00E-05 | 0.000216 | Putative methyltransferase family protein |
| AT5G27895 | 0.344648805 | 5.00E-05 | 0.000216 | transposable element gene |
| AT3G14320 | 0.344754312 | 0.00075 | 0.002548 | Zinc finger, C3HC4 type (RING finger) family protein |
| AT3G57765 | 0.344929898 | 0.0349 | 0.070608 | U2.3; snRNA |
| AT4G00390 | 0.345293874 | 5.00E-05 | 0.000216 | DNA-binding storekeeper protein-related transcriptional regulator |
| AT5G15970 | 0.345519829 | 5.00E-05 | 0.000216 | stress-responsive protein (KIN2) / stress-induced protein (KIN2) / cold-responsive protein (COR6.6) / cold-regulated protein (COR6.6) |
| AT2G04160 | 0.345858688 | 5.00E-05 | 0.000216 | Subtilisin-like serine endopeptidase family protein |
| AT5G23200 | 0.346044416 | 5.00E-05 | 0.000216 | NA |
| AT5G66570 | 0.347198486 | 5.00E-05 | 0.000216 | PS II oxygen-evolving complex 1 |
| AT5G20420 | 0.347627835 | 5.00E-05 | 0.000216 | chromatin remodeling 42 |
| AT4G04745 | 0.347904058 | 0.0004 | 0.001452 | NA |
| AT1G66880 | 0.347935812 | 5.00E-05 | 0.000216 | Protein kinase superfamily protein |
| AT3G01070 | 0.348768689 | 0.0038 | 0.010654 | early nodulin-like protein 16 |
| AT2G15090 | 0.348781472 | 5.00E-05 | 0.000216 | 3-ketoacyl-CoA synthase 8 |
| AT3G14680 | 0.349005501 | 5.00E-05 | 0.000216 | cytochrome P450, family 72, subfamily A, polypeptide 14 |
| AT5G20480 | 0.349252785 | 5.00E-05 | 0.000216 | EF-TU receptor |
| AT3G54180 | 0.349919516 | 0.00015 | 0.000596 | cyclin-dependent kinase B1;1 |
| AT1G63850 | 0.350031397 | 5.00E-05 | 0.000216 | BTB/POZ domain-containing protein |
| AT2G18980 | 0.350368772 | 5.00E-05 | 0.000216 | Peroxidase superfamily protein |
| AT1G62380 | 0.350541402 | 5.00E-05 | 0.000216 | ACC oxidase 2 |
| AT4G23690 | 0.351454793 | 0.00175 | 0.005406 | Disease resistance-responsive (dirigent-like protein) family protein |
| AT4G01590 | 0.35211745 | 5.00E-05 | 0.000216 | NA |
| AT5G04310 | 0.352570947 | 5.00E-05 | 0.000216 | Pectin lyase-like superfamily protein |
| AT2G43480 | 0.352728048 | 0.00275 | 0.008049 | Peroxidase superfamily protein |
| AT4G22560 | 0.352795732 | 0.0334 | 0.06805 | NA |
| AT2G28410 | 0.353042625 | 5.00E-05 | 0.000216 | NA |
| AT4G02770 | 0.35354355 | 5.00E-05 | 0.000216 | photosystem I subunit D-1 |
| AT2G20362 | 0.353867459 | 0.0121 | 0.028893 | NA |
| AT1G60960 | 0.35395543 | 5.00E-05 | 0.000216 | iron regulated transporter 3 |
| AT2G13820 | 0.353969945 | 5.00E-05 | 0.000216 | Bifunctional inhibitor/lipid-transfer protein/seed storage 2S albumin superfamily protein |
| AT1G71890 | 0.355601568 | 5.00E-05 | 0.000216 | Major facilitator superfamily protein |
| AT1G65520 | 0.355994531 | 5.00E-05 | 0.000216 | delta(3), delta(2)-enoyl CoA isomerase 1 |
| AT1G15210 | 0.356455519 | 5.00E-05 | 0.000216 | pleiotropic drug resistance 7 |
| AT1G37130 | 0.35649202 | 5.00E-05 | 0.000216 | nitrate reductase 2 |
| AT5G53050 | 0.356559121 | 5.00E-05 | 0.000216 | alpha/beta-Hydrolases superfamily protein |
| AT4G15910 | 0.358136014 | 5.00E-05 | 0.000216 | drought-induced 21 |
| AT4G01670 | 0.358484038 | 5.00E-05 | 0.000216 | NA |
| AT1G73120 | 0.358985816 | 5.00E-05 | 0.000216 | NA |
| AT3G19450 | 0.359008195 | 5.00E-05 | 0.000216 | GroES-like zinc-binding alcohol dehydrogenase family protein |
| AT5G41830 | 0.359449779 | 0.0001 | 0.000412 | RNI-like superfamily protein |
| AT3G48140 | 0.359989602 | 5.00E-05 | 0.000216 | B12D protein |
| AT2G43140 | 0.360520431 | 5.00E-05 | 0.000216 | basic helix-loop-helix (bHLH) DNA-binding superfamily protein |
| AT3G62540 | 0.360678068 | 5.00E-05 | 0.000216 | Pentatricopeptide repeat (PPR) superfamily protein |
| AT4G17240 | 0.360733206 | 5.00E-05 | 0.000216 | NA |
| AT1G15720 | 0.360783512 | 5.00E-05 | 0.000216 | TRF-like 5 |
| AT4G25470 | 0.360813948 | 0.0298 | 0.061893 | C-repeat/DRE binding factor 2 |
| AT2G20875 | 0.360814388 | 0.0112 | 0.027044 | epidermal patterning factor 1 |
| AT5G47550 | 0.360833897 | 5.00E-05 | 0.000216 | Cystatin/monellin superfamily protein |
| AT1G18320 | 0.360958462 | 5.00E-05 | 0.000216 | Mitochondrial import inner membrane translocase subunit Tim17/Tim22/Tim23 family protein |
| AT4G35640 | 0.360958913 | 0.039 | 0.077567 | serine acetyltransferase 3;2 |
| AT3G61320 | 0.361261814 | 5.00E-05 | 0.000216 | Bestrophin-like protein |
| AT4G16190 | 0.361726892 | 5.00E-05 | 0.000216 | Papain family cysteine protease |
| AT4G20320 | 0.362141572 | 5.00E-05 | 0.000216 | CTP synthase family protein |
| AT1G47840 | 0.362172235 | 5.00E-05 | 0.000216 | hexokinase 3 |
| AT3G43800 | 0.362381768 | 5.00E-05 | 0.000216 | glutathione S-transferase tau 27 |
| AT3G57060 | 0.36242971 | 5.00E-05 | 0.000216 | binding |
| AT1G52450 | 0.36263453 | 5.00E-05 | 0.000216 | Ubiquitin carboxyl-terminal hydrolase-related protein |
| AT3G58940 | 0.362704645 | 5.00E-05 | 0.000216 | F-box/RNI-like superfamily protein |
| AT3G53730 | 0.363246678 | 5.00E-05 | 0.000216 | Histone superfamily protein |
| AT1G51965 | 0.363410278 | 5.00E-05 | 0.000216 | ABA Overly-Sensitive 5 |
| AT3G05990 | 0.364045865 | 5.00E-05 | 0.000216 | Leucine-rich repeat (LRR) family protein |
| AT1G72070 | 0.364461121 | 5.00E-05 | 0.000216 | Chaperone DnaJ-domain superfamily protein |
| AT5G42200 | 0.364718728 | 5.00E-05 | 0.000216 | RING/U-box superfamily protein |
| AT2G38860 | 0.364824633 | 5.00E-05 | 0.000216 | Class I glutamine amidotransferase-like superfamily protein |
| AT4G13510 | 0.364834195 | 5.00E-05 | 0.000216 | ammonium transporter 1;1 |
| AT3G14070 | 0.364913533 | 5.00E-05 | 0.000216 | cation exchanger 9 |
| AT1G63090 | 0.365198807 | 5.00E-05 | 0.000216 | phloem protein 2-A11 |
| AT3G09260 | 0.365232364 | 5.00E-05 | 0.000216 | Glycosyl hydrolase superfamily protein |
| AT5G05890 | 0.365398496 | 5.00E-05 | 0.000216 | UDP-Glycosyltransferase superfamily protein |
| AT3G15510 | 0.365499303 | 5.00E-05 | 0.000216 | NAC domain containing protein 2 |
| AT3G46940 | 0.366083708 | 5.00E-05 | 0.000216 | DUTP-PYROPHOSPHATASE-LIKE 1 |
| AT1G55920 | 0.366169052 | 5.00E-05 | 0.000216 | serine acetyltransferase 2;1 |
| AT4G27260 | 0.366913785 | 5.00E-05 | 0.000216 | Auxin-responsive GH3 family protein |
| AT4G12010 | 0.367023063 | 5.00E-05 | 0.000216 | Disease resistance protein (TIR-NBS-LRR class) family |
| AT2G25350 | 0.367912163 | 5.00E-05 | 0.000216 | Phox (PX) domain-containing protein |
| AT5G58750 | 0.368213306 | 0.0001 | 0.000412 | NAD(P)-binding Rossmann-fold superfamily protein |
| AT4G23500 | 0.368262418 | 5.00E-05 | 0.000216 | Pectin lyase-like superfamily protein |
| AT4G39780 | 0.368406829 | 5.00E-05 | 0.000216 | Integrase-type DNA-binding superfamily protein |
| AT2G22170 | 0.36858384 | 5.00E-05 | 0.000216 | Lipase/lipooxygenase, PLAT/LH2 family protein |
| AT4G22860 | 0.368982361 | 5.00E-05 | 0.000216 | Cell cycle regulated microtubule associated protein |
| AT3G15990 | 0.369208369 | 5.00E-05 | 0.000216 | sulfate transporter 3;4 |
| AT1G24240 | 0.369423653 | 5.00E-05 | 0.000216 | Ribosomal protein L19 family protein |
| AT2G30870 | 0.369726809 | 5.00E-05 | 0.000216 | glutathione S-transferase PHI 10 |
| AT1G77885 | 0.370203089 | 5.00E-05 | 0.000216 | NA |
| AT3G44120 | 0.370215554 | 0.0003 | 0.00112 | F-box and associated interaction domains-containing protein |
| AT5G49800 | 0.37048424 | 5.00E-05 | 0.000216 | Polyketide cyclase/dehydrase and lipid transport superfamily protein |
| AT3G13910 | 0.370607889 | 5.00E-05 | 0.000216 | Protein of unknown function (DUF3511) |
| AT1G62350 | 0.371348544 | 5.00E-05 | 0.000216 | Pentatricopeptide repeat (PPR) superfamily protein |
| AT1G08380 | 0.37202078 | 5.00E-05 | 0.000216 | photosystem I subunit O |
| AT5G66985 | 0.372150572 | 5.00E-05 | 0.000216 | NA |
| AT3G16430 | 0.372153267 | 5.00E-05 | 0.000216 | jacalin-related lectin 31 |
| AT4G30190 | 0.372389717 | 5.00E-05 | 0.000216 | H(+)-ATPase 2 |
| AT1G49510 | 0.372557977 | 5.00E-05 | 0.000216 | embryo defective 1273 |
| AT4G13070 | 0.372619145 | 5.00E-05 | 0.000216 | RNA-binding CRS1 / YhbY (CRM) domain protein |
| AT1G58160 | 0.372974689 | 0.0128 | 0.030352 | Mannose-binding lectin superfamily protein |
| AT4G11310 | 0.372980506 | 5.00E-05 | 0.000216 | Papain family cysteine protease |
| AT3G13520 | 0.373199922 | 5.00E-05 | 0.000216 | arabinogalactan protein 12 |
| AT1G34340 | 0.374099786 | 5.00E-05 | 0.000216 | alpha/beta-Hydrolases superfamily protein |
| AT1G47620 | 0.374748944 | 5.00E-05 | 0.000216 | cytochrome P450, family 96, subfamily A, polypeptide 8 |
| AT2G07808 | 0.375164936 | 0.0232 | 0.050116 | NA |
| AT1G63150 | 0.375236375 | 5.00E-05 | 0.000216 | Tetratricopeptide repeat (TPR)-like superfamily protein |
| AT5G02970 | 0.375537566 | 5.00E-05 | 0.000216 | alpha/beta-Hydrolases superfamily protein |
| AT5G66380 | 0.375554544 | 5.00E-05 | 0.000216 | folate transporter 1 |
| AT3G51300 | 0.375814335 | 0.00555 | 0.01481 | RHO-related protein from plants 1 |
| AT5G05760 | 0.375896986 | 5.00E-05 | 0.000216 | syntaxin of plants 31 |
| AT5G17350 | 0.376300296 | 0.017 | 0.03851 | NA |
| AT1G55370 | 0.37685863 | 5.00E-05 | 0.000216 | NDH-dependent cyclic electron flow 5 |
| AT3G61310 | 0.377030228 | 5.00E-05 | 0.000216 | AT hook motif DNA-binding family protein |
| AT3G20120 | 0.377193416 | 5.00E-05 | 0.000216 | cytochrome P450, family 705, subfamily A, polypeptide 21 |
| AT3G24450 | 0.377460856 | 0.01025 | 0.025055 | Heavy metal transport/detoxification superfamily protein |
| AT1G05300 | 0.378704666 | 0.0005 | 0.001772 | zinc transporter 5 precursor |
| AT2G01420 | 0.37880596 | 5.00E-05 | 0.000216 | Auxin efflux carrier family protein |
| AT1G03870 | 0.379132883 | 0.00975 | 0.024023 | FASCICLIN-like arabinoogalactan 9 |
| AT3G27850 | 0.379481918 | 5.00E-05 | 0.000216 | ribosomal protein L12-C |
| AT1G07160 | 0.380243502 | 0.0021 | 0.006356 | Protein phosphatase 2C family protein |
| AT1G54410 | 0.380394748 | 5.00E-05 | 0.000216 | dehydrin family protein |
| AT3G08940 | 0.380784659 | 0.0009 | 0.003 | light harvesting complex photosystem II |
| AT3G14610 | 0.381157856 | 5.00E-05 | 0.000216 | cytochrome P450, family 72, subfamily A, polypeptide 7 |
| AT1G64625 | 0.381356356 | 0.00255 | 0.007539 | Serine/threonine-protein kinase WNK (With No Lysine)-related |
| AT2G33320 | 0.381485636 | 5.00E-05 | 0.000216 | Calcium-dependent lipid-binding (CaLB domain) family protein |
| AT1G66330 | 0.381611826 | 5.00E-05 | 0.000216 | senescence-associated family protein |
| AT1G62305 | 0.381730168 | 5.00E-05 | 0.000216 | Core-2/I-branching beta-1,6-N-acetylglucosaminyltransferase family protein |
| AT2G17845 | 0.38181954 | 0.00085 | 0.00285 | NAD(P)-binding Rossmann-fold superfamily protein |
| AT2G26710 | 0.382287108 | 5.00E-05 | 0.000216 | Cytochrome P450 superfamily protein |
| AT2G30890 | 0.382847801 | 0.00035 | 0.001288 | Cytochrome b561/ferric reductase transmembrane protein family |
| AT4G01480 | 0.382954925 | 0.0143 | 0.033283 | pyrophosphorylase 5 |
| AT2G17480 | 0.38317781 | 5.00E-05 | 0.000216 | Seven transmembrane MLO family protein |
| AT1G80090 | 0.383526272 | 5.00E-05 | 0.000216 | Cystathionine beta-synthase (CBS) family protein |
| AT5G44490 | 0.384135845 | 5.00E-05 | 0.000216 | FBD, F-box, Skp2-like and Leucine Rich Repeat domains containing protein |
| AT4G03600 | 0.385351748 | 0.028 | 0.058741 | NA |
| AT2G05812 | 0.386240587 | 5.00E-05 | 0.000216 | NA |
| AT3G51420 | 0.386270577 | 5.00E-05 | 0.000216 | strictosidine synthase-like 4 |
| AT1G65040 | 0.386583098 | 5.00E-05 | 0.000216 | RING/U-box superfamily protein |
| AT2G41290 | 0.386945137 | 5.00E-05 | 0.000216 | strictosidine synthase-like 2 |
| AT3G26780 | 0.387454239 | 5.00E-05 | 0.000216 | Phosphoglycerate mutase family protein |
| AT1G09090 | 0.387893631 | 0.016 | 0.036595 | respiratory burst oxidase homolog B |
| AT5G25050 | 0.388041264 | 5.00E-05 | 0.000216 | Major facilitator superfamily protein |
| AT5G48980 | 0.388136274 | 0.0055 | 0.014692 | Galactose oxidase/kelch repeat superfamily protein |
| AT2G02850 | 0.388138363 | 5.00E-05 | 0.000216 | plantacyanin |
| AT3G50480 | 0.388784427 | 0.0074 | 0.018939 | homolog of RPW8 4 |
| AT1G51170 | 0.38904254 | 5.00E-05 | 0.000216 | Protein kinase superfamily protein |
| AT3G01680 | 0.389432175 | 5.00E-05 | 0.000216 | NA |
| AT1G12890 | 0.389891317 | 0.00105 | 0.003446 | Integrase-type DNA-binding superfamily protein |
| AT3G12110 | 0.389908336 | 5.00E-05 | 0.000216 | actin-11 |
| AT3G54890 | 0.390795729 | 5.00E-05 | 0.000216 | photosystem I light harvesting complex gene 1 |
| AT4G21770 | 0.391208963 | 5.00E-05 | 0.000216 | Pseudouridine synthase family protein |
| AT3G28180 | 0.391252687 | 5.00E-05 | 0.000216 | Cellulose-synthase-like C4 |
| AT3G43670 | 0.391258993 | 5.00E-05 | 0.000216 | Copper amine oxidase family protein |
| AT5G49550 | 0.392011321 | 5.00E-05 | 0.000216 | NA |
| AT4G11440 | 0.392277705 | 5.00E-05 | 0.000216 | Mitochondrial substrate carrier family protein |
| AT1G52710 | 0.392430002 | 5.00E-05 | 0.000216 | Rubredoxin-like superfamily protein |
| AT1G55670 | 0.392575329 | 5.00E-05 | 0.000216 | photosystem I subunit G |
| AT5G15030 | 0.392594538 | 5.00E-05 | 0.000216 | Paired amphipathic helix (PAH2) superfamily protein |
| AT5G48570 | 0.392647629 | 5.00E-05 | 0.000216 | FKBP-type peptidyl-prolyl cis-trans isomerase family protein |
| AT1G33520 | 0.393087533 | 5.00E-05 | 0.000216 | D111/G-patch domain-containing protein |
| AT1G34430 | 0.393481233 | 5.00E-05 | 0.000216 | 2-oxoacid dehydrogenases acyltransferase family protein |
| AT1G32690 | 0.393575345 | 0.01755 | 0.039567 | NA |
| AT5G19940 | 0.393773828 | 0.0002 | 0.000777 | Plastid-lipid associated protein PAP / fibrillin family protein |
| AT1G64080 | 0.394572987 | 5.00E-05 | 0.000216 | NA |
| AT1G66350 | 0.395323979 | 5.00E-05 | 0.000216 | RGA-like 1 |
| AT1G29590 | 0.395631813 | 0.00455 | 0.012443 | Eukaryotic initiation factor 4E protein |
| AT3G16460 | 0.39573119 | 5.00E-05 | 0.000216 | Mannose-binding lectin superfamily protein |
| AT1G15880 | 0.39578747 | 5.00E-05 | 0.000216 | golgi snare 11 |
| AT4G26740 | 0.396111578 | 5.00E-05 | 0.000216 | seed gene 1 |
| AT3G15630 | 0.396249434 | 0.01145 | 0.027577 | NA |
| AT1G53430 | 0.397127745 | 5.00E-05 | 0.000216 | Leucine-rich repeat transmembrane protein kinase |
| AT1G28400 | 0.397546413 | 5.00E-05 | 0.000216 | NA |
| AT3G24840 | 0.397939081 | 5.00E-05 | 0.000216 | Sec14p-like phosphatidylinositol transfer family protein |
| AT4G19440 | 0.398112479 | 5.00E-05 | 0.000216 | Tetratricopeptide repeat (TPR)-like superfamily protein |
| AT5G63760 | 0.398647649 | 5.00E-05 | 0.000216 | RING/U-box superfamily protein |
| AT1G47540 | 0.398860892 | 5.00E-05 | 0.000216 | Scorpion toxin-like knottin superfamily protein |
| AT5G41590 | 0.398863201 | 0.01515 | 0.034918 | Protein of unknown function (DUF567) |
| AT5G57240 | 0.398893968 | 5.00E-05 | 0.000216 | OSBP(oxysterol binding protein)-related protein 4C |
| AT5G58260 | 0.399150716 | 5.00E-05 | 0.000216 | oxidoreductases, acting on NADH or NADPH, quinone or similar compound as acceptor |
| AT5G42240 | 0.399294878 | 5.00E-05 | 0.000216 | serine carboxypeptidase-like 42 |
| AT3G02480 | 0.399782841 | 5.00E-05 | 0.000216 | Late embryogenesis abundant protein (LEA) family protein |
| AT1G23740 | 0.399965143 | 5.00E-05 | 0.000216 | Oxidoreductase, zinc-binding dehydrogenase family protein |
| AT5G02820 | 0.400016472 | 5.00E-05 | 0.000216 | Spo11/DNA topoisomerase VI, subunit A protein |
| AT2G03880 | 0.400088536 | 5.00E-05 | 0.000216 | Pentatricopeptide repeat (PPR) superfamily protein |
| AT4G24780 | 0.400356081 | 5.00E-05 | 0.000216 | Pectin lyase-like superfamily protein |
| AT1G32900 | 0.400883599 | 5.00E-05 | 0.000216 | UDP-Glycosyltransferase superfamily protein |
| AT1G07620 | 0.401025477 | 5.00E-05 | 0.000216 | GTP-binding protein Obg/CgtA |
| AT4G24580 | 0.401090633 | 5.00E-05 | 0.000216 | Rho GTPase activation protein (RhoGAP) with PH domain |
| AT5G56910 | 0.401499551 | 5.00E-05 | 0.000216 | Proteinase inhibitor I25, cystatin, conserved region |
| AT4G25530 | 0.401506597 | 5.00E-05 | 0.000216 | FLOWERING WAGENINGEN |
| AT3G50520 | 0.401627908 | 5.00E-05 | 0.000216 | Phosphoglycerate mutase family protein |
| AT1G12070 | 0.402367275 | 5.00E-05 | 0.000216 | Immunoglobulin E-set superfamily protein |
| AT5G02600 | 0.402791565 | 0.006 | 0.015829 | Heavy metal transport/detoxification superfamily protein |
| AT2G19590 | 0.402825224 | 5.00E-05 | 0.000216 | ACC oxidase 1 |
| AT3G25900 | 0.402860735 | 5.00E-05 | 0.000216 | Homocysteine S-methyltransferase family protein |
| AT4G13660 | 0.403133558 | 5.00E-05 | 0.000216 | pinoresinol reductase 2 |
| AT1G58440 | 0.403399067 | 5.00E-05 | 0.000216 | FAD/NAD(P)-binding oxidoreductase family protein |
| AT4G25300 | 0.403546249 | 0.0026 | 0.007668 | 2-oxoglutarate (2OG) and Fe(II)-dependent oxygenase superfamily protein |
| AT5G49960 | 0.403731564 | 5.00E-05 | 0.000216 | NA |
| AT3G49570 | 0.403952721 | 0.0004 | 0.001452 | response to low sulfur 3 |
| AT2G34420 | 0.404060034 | 5.00E-05 | 0.000216 | photosystem II light harvesting complex gene B1B2 |
| AT3G21800 | 0.404101131 | 5.00E-05 | 0.000216 | UDP-glucosyl transferase 71B8 |
| AT1G29160 | 0.404264326 | 0.0349 | 0.070608 | Dof-type zinc finger DNA-binding family protein |
| AT2G42220 | 0.40460228 | 0.0101 | 0.024735 | Rhodanese/Cell cycle control phosphatase superfamily protein |
| AT1G59700 | 0.404838353 | 5.00E-05 | 0.000216 | glutathione S-transferase TAU 16 |
| AT5G20410 | 0.404969844 | 5.00E-05 | 0.000216 | monogalactosyldiacylglycerol synthase 2 |
| AT5G48790 | 0.405501869 | 5.00E-05 | 0.000216 | Domain of unknown function (DUF1995) |
| AT2G39100 | 0.406154556 | 5.00E-05 | 0.000216 | RING/U-box superfamily protein |
| AT1G28580 | 0.406198595 | 5.00E-05 | 0.000216 | GDSL-like Lipase/Acylhydrolase superfamily protein |
| AT1G18570 | 0.4063413 | 0.0044 | 0.012091 | myb domain protein 51 |
| AT3G43210 | 0.40639988 | 5.00E-05 | 0.000216 | ATP binding microtubule motor family protein |
| AT3G04290 | 0.406449541 | 0.0053 | 0.014238 | Li-tolerant lipase 1 |
| AT4G13195 | 0.406450833 | 0.0091 | 0.022654 | CLAVATA3/ESR-RELATED 44 |
| AT4G02380 | 0.406667806 | 5.00E-05 | 0.000216 | senescence-associated gene 21 |
| AT3G61160 | 0.407599437 | 5.00E-05 | 0.000216 | Protein kinase superfamily protein |
| AT3G61470 | 0.407696865 | 5.00E-05 | 0.000216 | photosystem I light harvesting complex gene 2 |
| AT5G51640 | 0.407943765 | 5.00E-05 | 0.000216 | Plant protein of unknown function (DUF828) |
| AT2G40920 | 0.40795011 | 5.00E-05 | 0.000216 | F-box and associated interaction domains-containing protein |
| AT2G36470 | 0.408744378 | 5.00E-05 | 0.000216 | Plant protein of unknown function (DUF868) |
| AT5G60810 | 0.408882462 | 0.04995 | 0.095382 | root meristem growth factor 1 |
| AT2G37260 | 0.408925258 | 5.00E-05 | 0.000216 | WRKY family transcription factor family protein |
| AT5G03570 | 0.409559269 | 5.00E-05 | 0.000216 | iron regulated 2 |
| AT3G26300 | 0.409691828 | 5.00E-05 | 0.000216 | cytochrome P450, family 71, subfamily B, polypeptide 34 |
| AT5G60340 | 0.409863438 | 5.00E-05 | 0.000216 | P-loop containing nucleoside triphosphate hydrolases superfamily protein |
| AT1G43580 | 0.409912852 | 5.00E-05 | 0.000216 | Sphingomyelin synthetase family protein |
| AT2G28450 | 0.410108981 | 5.00E-05 | 0.000216 | zinc finger (CCCH-type) family protein |
| AT4G19550 | 0.410313736 | 5.00E-05 | 0.000216 | zinc ion binding;transcription regulators |
| AT3G18710 | 0.410492188 | 0.0034 | 0.009711 | plant U-box 29 |
| AT2G38310 | 0.410963832 | 5.00E-05 | 0.000216 | PYR1-like 4 |
| AT4G23710 | 0.411007943 | 5.00E-05 | 0.000216 | vacuolar ATP synthase subunit G2 |
| AT3G60440 | 0.411837738 | 5.00E-05 | 0.000216 | Phosphoglycerate mutase family protein |
| AT4G39510 | 0.411923728 | 5.00E-05 | 0.000216 | cytochrome P450, family 96, subfamily A, polypeptide 12 |
| AT4G38980 | 0.412093276 | 5.00E-05 | 0.000216 | NA |
| AT4G16745 | 0.412453508 | 5.00E-05 | 0.000216 | Exostosin family protein |
| AT2G45050 | 0.412552138 | 5.00E-05 | 0.000216 | GATA transcription factor 2 |
| AT3G05760 | 0.412628205 | 5.00E-05 | 0.000216 | C2H2 and C2HC zinc fingers superfamily protein |
| AT1G43650 | 0.412634022 | 0.00685 | 0.017737 | nodulin MtN21 /EamA-like transporter family protein |
| AT1G02920 | 0.413007303 | 5.00E-05 | 0.000216 | glutathione S-transferase 7 |
| AT5G42440 | 0.413100219 | 0.02205 | 0.047942 | Protein kinase superfamily protein |
| AT5G47440 | 0.413175021 | 0.00735 | 0.018837 | Plant protein of unknown function (DUF828) with plant pleckstrin homology-like region |
| AT1G12880 | 0.413490504 | 5.00E-05 | 0.000216 | nudix hydrolase homolog 12 |
| AT1G72890 | 0.413630376 | 0.01085 | 0.026292 | Disease resistance protein (TIR-NBS class) |
| AT1G61340 | 0.414116713 | 5.00E-05 | 0.000216 | F-box family protein |
| AT5G46530 | 0.414166572 | 0.00015 | 0.000596 | AWPM-19-like family protein |
| AT3G06120 | 0.414341871 | 5.00E-05 | 0.000216 | basic helix-loop-helix (bHLH) DNA-binding superfamily protein |
| AT3G46540 | 0.414702637 | 5.00E-05 | 0.000216 | ENTH/VHS family protein |
| AT5G17240 | 0.414740467 | 5.00E-05 | 0.000216 | SET domain group 40 |
| AT5G56980 | 0.415167353 | 5.00E-05 | 0.000216 | NA |
| AT3G60530 | 0.415305901 | 5.00E-05 | 0.000216 | GATA transcription factor 4 |
| AT4G30090 | 0.415358172 | 0.0032 | 0.009199 | embryo defective 1353 |
| AT4G17600 | 0.415577422 | 5.00E-05 | 0.000216 | Chlorophyll A-B binding family protein |
| AT4G13100 | 0.415584556 | 5.00E-05 | 0.000216 | RING/U-box superfamily protein |
| AT1G22340 | 0.415593839 | 0.01395 | 0.032571 | UDP-glucosyl transferase 85A7 |
| AT1G24090 | 0.415655894 | 5.00E-05 | 0.000216 | RNase H family protein |
| AT2G39050 | 0.416117559 | 0.00415 | 0.01148 | hydroxyproline-rich glycoprotein family protein |
| AT5G04230 | 0.416773227 | 5.00E-05 | 0.000216 | phenyl alanine ammonia-lyase 3 |
| AT2G21050 | 0.416795428 | 5.00E-05 | 0.000216 | like AUXIN RESISTANT 2 |
| AT3G11210 | 0.417349567 | 5.00E-05 | 0.000216 | SGNH hydrolase-type esterase superfamily protein |
| AT5G39240 | 0.417591416 | 0.00725 | 0.018614 | NA |
| AT2G32765 | 0.41770547 | 5.00E-05 | 0.000216 | small ubiquitinrelated modifier 5 |
| AT4G21865 | 0.418072718 | 0.0007 | 0.002394 | NA |
| AT1G26870 | 0.418358399 | 0.01445 | 0.033591 | NAC (No Apical Meristem) domain transcriptional regulator superfamily protein |
| AT5G12050 | 0.418591972 | 0.00145 | 0.004592 | NA |
| AT3G51290 | 0.41865309 | 5.00E-05 | 0.000216 | Protein of unknown function (DUF630) ;Protein of unknown function (DUF632) |
| AT5G49890 | 0.419022416 | 5.00E-05 | 0.000216 | chloride channel C |
| AT4G11080 | 0.419068663 | 5.00E-05 | 0.000216 | HMG (high mobility group) box protein |
| AT3G44300 | 0.419069498 | 0.00295 | 0.008554 | nitrilase 2 |
| AT1G62790 | 0.419071691 | 5.00E-05 | 0.000216 | Bifunctional inhibitor/lipid-transfer protein/seed storage 2S albumin superfamily protein |
| AT4G12060 | 0.419407327 | 5.00E-05 | 0.000216 | Double Clp-N motif protein |
| AT4G09160 | 0.419975529 | 5.00E-05 | 0.000216 | SEC14 cytosolic factor family protein / phosphoglyceride transfer family protein |
| AT5G58990 | 0.419978125 | 5.00E-05 | 0.000216 | NA |
| AT3G44450 | 0.420135839 | 0.0303 | 0.062778 | NA |
| AT5G07440 | 0.420147075 | 5.00E-05 | 0.000216 | glutamate dehydrogenase 2 |
| AT3G27340 | 0.420343732 | 5.00E-05 | 0.000216 | NA |
| AT3G63480 | 0.420685927 | 0.00095 | 0.003145 | ATP binding microtubule motor family protein |
| AT1G26780 | 0.420937001 | 0.0434 | 0.084999 | myb domain protein 117 |
| AT5G04660 | 0.421212003 | 0.0001 | 0.000412 | cytochrome P450, family 77, subfamily A, polypeptide 4 |
| AT3G21670 | 0.421525105 | 5.00E-05 | 0.000216 | Major facilitator superfamily protein |
| AT4G23030 | 0.422566345 | 5.00E-05 | 0.000216 | MATE efflux family protein |
| AT1G25230 | 0.422847983 | 5.00E-05 | 0.000216 | Calcineurin-like metallo-phosphoesterase superfamily protein |
| AT3G45638 | 0.42305452 | 5.00E-05 | 0.000216 | other RNA |
| AT4G10130 | 0.423142528 | 5.00E-05 | 0.000216 | DNAJ heat shock N-terminal domain-containing protein |
| AT4G05180 | 0.423422547 | 0.0007 | 0.002394 | photosystem II subunit Q-2 |
| AT1G80190 | 0.423515679 | 5.00E-05 | 0.000216 | partner of SLD five 1 |
| AT3G22680 | 0.423866446 | 5.00E-05 | 0.000216 | RNA-DIRECTED DNA METHYLATION 1 |
| AT2G15960 | 0.424229846 | 5.00E-05 | 0.000216 | NA |
| AT1G80830 | 0.424362955 | 5.00E-05 | 0.000216 | natural resistance-associated macrophage protein 1 |
| AT5G44160 | 0.424492741 | 0.00915 | 0.022758 | C2H2-like zinc finger protein |
| AT5G24530 | 0.424651951 | 5.00E-05 | 0.000216 | 2-oxoglutarate (2OG) and Fe(II)-dependent oxygenase superfamily protein |
| AT2G28200 | 0.424836937 | 5.00E-05 | 0.000216 | C2H2-type zinc finger family protein |
| AT3G22550 | 0.424909687 | 5.00E-05 | 0.000216 | Protein of unknown function (DUF581) |
| AT2G07170 | 0.425774596 | 5.00E-05 | 0.000216 | ARM repeat superfamily protein |
| AT3G15810 | 0.426244839 | 5.00E-05 | 0.000216 | Protein of unknown function (DUF567) |
| AT3G25597 | 0.426367545 | 0.00095 | 0.003145 | NA |
| AT2G34980 | 0.426499074 | 0.01575 | 0.036106 | phosphatidylinositolglycan synthase family protein |
| AT5G05250 | 0.426835956 | 5.00E-05 | 0.000216 | NA |
| AT5G14110 | 0.426949909 | 0.0207 | 0.045529 | Protein of unknown function (DUF 3339) |
| AT4G20325 | 0.427549959 | 5.00E-05 | 0.000216 | NA |
| AT1G16360 | 0.427665096 | 0.00025 | 0.000951 | LEM3 (ligand-effect modulator 3) family protein / CDC50 family protein |
| AT1G63670 | 0.427875668 | 5.00E-05 | 0.000216 | Protein of unknown function (DUF3741) |
| AT1G01520 | 0.42789969 | 5.00E-05 | 0.000216 | Homeodomain-like superfamily protein |
| AT2G42320 | 0.428333815 | 5.00E-05 | 0.000216 | nucleolar protein gar2-related |
| AT2G36570 | 0.428503267 | 5.00E-05 | 0.000216 | Leucine-rich repeat protein kinase family protein |
| AT5G10140 | 0.42865583 | 5.00E-05 | 0.000216 | K-box region and MADS-box transcription factor family protein |
| AT1G22360 | 0.428985952 | 5.00E-05 | 0.000216 | UDP-glucosyl transferase 85A2 |
| AT4G38660 | 0.429140423 | 5.00E-05 | 0.000216 | Pathogenesis-related thaumatin superfamily protein |
| AT3G48380 | 0.429323845 | 5.00E-05 | 0.000216 | Peptidase C78, ubiquitin fold modifier-specific peptidase 1/ 2 |
| AT1G65030 | 0.429628579 | 5.00E-05 | 0.000216 | Transducin/WD40 repeat-like superfamily protein |
| AT3G23810 | 0.429934585 | 5.00E-05 | 0.000216 | S-adenosyl-l-homocysteine (SAH) hydrolase 2 |
| AT2G44180 | 0.429952786 | 5.00E-05 | 0.000216 | methionine aminopeptidase 2A |
| AT2G04400 | 0.430121668 | 5.00E-05 | 0.000216 | Aldolase-type TIM barrel family protein |
| AT1G06475 | 0.43044081 | 0.01685 | 0.038211 | NA |
| AT5G47230 | 0.431067844 | 5.00E-05 | 0.000216 | ethylene responsive element binding factor 5 |
| AT3G12150 | 0.431128366 | 5.00E-05 | 0.000216 | NA |
| AT2G16720 | 0.431372308 | 0.0015 | 0.004727 | myb domain protein 7 |
| AT1G60680 | 0.431597988 | 0.01085 | 0.026292 | NAD(P)-linked oxidoreductase superfamily protein |
| AT5G44110 | 0.431703359 | 5.00E-05 | 0.000216 | P-loop containing nucleoside triphosphate hydrolases superfamily protein |
| AT4G27670 | 0.43183205 | 5.00E-05 | 0.000216 | heat shock protein 21 |
| AT1G72020 | 0.431928201 | 5.00E-05 | 0.000216 | NA |
| AT1G48660 | 0.432742272 | 5.00E-05 | 0.000216 | Auxin-responsive GH3 family protein |
| AT3G54910 | 0.432759443 | 5.00E-05 | 0.000216 | RNI-like superfamily protein |
| AT5G22130 | 0.43317436 | 5.00E-05 | 0.000216 | mannosyltransferase family protein |
| AT5G63990 | 0.434130249 | 5.00E-05 | 0.000216 | Inositol monophosphatase family protein |
| AT1G62150 | 0.434184596 | 5.00E-05 | 0.000216 | Mitochondrial transcription termination factor family protein |
| AT1G20020 | 0.434341426 | 0.0009 | 0.003 | ferredoxin-NADP(+)-oxidoreductase 2 |
| AT1G23890 | 0.434346689 | 5.00E-05 | 0.000216 | NHL domain-containing protein |
| AT1G48480 | 0.434409105 | 5.00E-05 | 0.000216 | receptor-like kinase 1 |
| AT5G17990 | 0.434470057 | 5.00E-05 | 0.000216 | tryptophan biosynthesis 1 |
| AT2G38720 | 0.434834469 | 5.00E-05 | 0.000216 | microtubule-associated protein 65-5 |
| AT2G26180 | 0.434965308 | 0.00835 | 0.021017 | IQ-domain 6 |
| AT3G13310 | 0.435031743 | 5.00E-05 | 0.000216 | Chaperone DnaJ-domain superfamily protein |
| AT4G30440 | 0.435132862 | 5.00E-05 | 0.000216 | UDP-D-glucuronate 4-epimerase 1 |
| AT1G01355 | 0.435231046 | 0.04605 | 0.089164 | Putative endonuclease or glycosyl hydrolase |
| AT3G29310 | 0.435456939 | 5.00E-05 | 0.000216 | calmodulin-binding protein-related |
| AT2G01990 | 0.435456973 | 0.0146 | 0.033884 | NA |
| AT2G01660 | 0.435976944 | 0.01375 | 0.032179 | plasmodesmata-located protein 6 |
| AT4G34160 | 0.436080757 | 5.00E-05 | 0.000216 | CYCLIN D3;1 |
| AT4G24026 | 0.436540912 | 0.0345 | 0.069924 | NA |
| AT1G28110 | 0.437173794 | 5.00E-05 | 0.000216 | serine carboxypeptidase-like 45 |
| AT5G09800 | 0.437249602 | 5.00E-05 | 0.000216 | ARM repeat superfamily protein |
| AT1G62640 | 0.437474314 | 5.00E-05 | 0.000216 | 3-ketoacyl-acyl carrier protein synthase III |
| AT3G11720 | 0.437689861 | 5.00E-05 | 0.000216 | Polyketide cyclase/dehydrase and lipid transport superfamily protein |
| AT3G44890 | 0.437807516 | 5.00E-05 | 0.000216 | ribosomal protein L9 |
| AT3G26612 | 0.438131796 | 0.00045 | 0.001612 | other RNA |
| AT5G35660 | 0.438179923 | 5.00E-05 | 0.000216 | Glycine-rich protein family |
| AT4G35160 | 0.438429028 | 5.00E-05 | 0.000216 | O-methyltransferase family protein |
| AT4G16141 | 0.438467265 | 5.00E-05 | 0.000216 | GATA type zinc finger transcription factor family protein |
| AT5G23510 | 0.438600528 | 5.00E-05 | 0.000216 | NA |
| AT2G01505 | 0.438686148 | 0.0001 | 0.000412 | CLAVATA3/ESR-RELATED 16 |
| AT1G64640 | 0.438787479 | 5.00E-05 | 0.000216 | early nodulin-like protein 8 |
| AT5G11000 | 0.438829814 | 5.00E-05 | 0.000216 | Plant protein of unknown function (DUF868) |
| AT1G07440 | 0.43890839 | 5.00E-05 | 0.000216 | NAD(P)-binding Rossmann-fold superfamily protein |
| AT2G47130 | 0.439259424 | 5.00E-05 | 0.000216 | NAD(P)-binding Rossmann-fold superfamily protein |
| AT5G17840 | 0.440009164 | 5.00E-05 | 0.000216 | DnaJ/Hsp40 cysteine-rich domain superfamily protein |
| AT4G24570 | 0.440472869 | 5.00E-05 | 0.000216 | dicarboxylate carrier 2 |
| AT1G31320 | 0.440542462 | 5.00E-05 | 0.000216 | LOB domain-containing protein 4 |
| AT1G47960 | 0.441248147 | 5.00E-05 | 0.000216 | cell wall / vacuolar inhibitor of fructosidase 1 |
| AT1G14060 | 0.44180536 | 5.00E-05 | 0.000216 | GCK domain-containing protein |
| AT5G28040 | 0.441843326 | 5.00E-05 | 0.000216 | DNA-binding storekeeper protein-related transcriptional regulator |
| AT1G22440 | 0.442099088 | 5.00E-05 | 0.000216 | Zinc-binding alcohol dehydrogenase family protein |
| AT4G01840 | 0.442163041 | 5.00E-05 | 0.000216 | Ca2+ activated outward rectifying K+ channel 5 |
| AT5G38005 | 0.442175185 | 0.00015 | 0.000596 | other RNA |
| AT5G50300 | 0.442438025 | 5.00E-05 | 0.000216 | Xanthine/uracil permease family protein |
| AT5G03345 | 0.442751384 | 5.00E-05 | 0.000216 | NA |
| AT5G35738 | 0.442969507 | 0.02985 | 0.061944 | NA |
| AT4G20030 | 0.443403728 | 5.00E-05 | 0.000216 | RNA-binding (RRM/RBD/RNP motifs) family protein |
| AT3G62150 | 0.443417306 | 5.00E-05 | 0.000216 | P-glycoprotein 21 |
| AT4G36660 | 0.443482455 | 5.00E-05 | 0.000216 | Protein of unknown function (DUF1195) |
| AT4G39210 | 0.443541836 | 5.00E-05 | 0.000216 | Glucose-1-phosphate adenylyltransferase family protein |
| AT1G14870 | 0.443570783 | 5.00E-05 | 0.000216 | PLANT CADMIUM RESISTANCE 2 |
| AT1G34160 | 0.443571026 | 5.00E-05 | 0.000216 | Tetratricopeptide repeat (TPR)-like superfamily protein |
| AT5G59750 | 0.443899571 | 5.00E-05 | 0.000216 | DHBP synthase RibB-like alpha/beta domain;GTP cyclohydrolase II |
| AT3G16100 | 0.444347017 | 5.00E-05 | 0.000216 | RAB GTPase homolog G3C |
| AT3G16420 | 0.444640247 | 5.00E-05 | 0.000216 | PYK10-binding protein 1 |
| AT4G31820 | 0.445265463 | 5.00E-05 | 0.000216 | Phototropic-responsive NPH3 family protein |
| AT5G18470 | 0.4454147 | 5.00E-05 | 0.000216 | Curculin-like (mannose-binding) lectin family protein |
| AT5G44190 | 0.445708268 | 5.00E-05 | 0.000216 | GOLDEN2-like 2 |
| AT3G51410 | 0.446205363 | 5.00E-05 | 0.000216 | Arabidopsis protein of unknown function (DUF241) |
| AT2G42190 | 0.446624425 | 5.00E-05 | 0.000216 | NA |
| AT1G74090 | 0.447134081 | 5.00E-05 | 0.000216 | desulfo-glucosinolate sulfotransferase 18 |
| AT5G11230 | 0.447203319 | 5.00E-05 | 0.000216 | Nucleotide-sugar transporter family protein |
| AT1G12410 | 0.447445669 | 5.00E-05 | 0.000216 | CLP protease proteolytic subunit 2 |
| AT5G65020 | 0.447589965 | 5.00E-05 | 0.000216 | annexin 2 |
| AT4G14030 | 0.447714548 | 5.00E-05 | 0.000216 | selenium-binding protein 1 |
| AT5G49290 | 0.447872364 | 5.00E-05 | 0.000216 | receptor like protein 56 |
| AT4G03420 | 0.447886949 | 5.00E-05 | 0.000216 | Protein of unknown function (DUF789) |
| AT2G24570 | 0.449238484 | 5.00E-05 | 0.000216 | WRKY DNA-binding protein 17 |
| AT3G05150 | 0.449615734 | 0.0002 | 0.000777 | Major facilitator superfamily protein |
| AT3G14350 | 0.449751605 | 5.00E-05 | 0.000216 | STRUBBELIG-receptor family 7 |
| AT1G35420 | 0.450222176 | 5.00E-05 | 0.000216 | alpha/beta-Hydrolases superfamily protein |
| AT4G38550 | 0.450564605 | 0.01545 | 0.035518 | Arabidopsis phospholipase-like protein (PEARLI 4) family |
| AT1G61310 | 0.450663686 | 5.00E-05 | 0.000216 | LRR and NB-ARC domains-containing disease resistance protein |
| AT2G35060 | 0.45078793 | 5.00E-05 | 0.000216 | K+ uptake permease 11 |
| AT1G72430 | 0.450854371 | 5.00E-05 | 0.000216 | SAUR-like auxin-responsive protein family |
| AT2G40330 | 0.450980298 | 5.00E-05 | 0.000216 | PYR1-like 6 |
| AT5G28646 | 0.45107768 | 5.00E-05 | 0.000216 | TPX2 (targeting protein for Xklp2) protein family |
| AT5G56940 | 0.451410222 | 5.00E-05 | 0.000216 | Ribosomal protein S16 family protein |
| AT2G47520 | 0.451683146 | 5.00E-05 | 0.000216 | Integrase-type DNA-binding superfamily protein |
| AT2G31160 | 0.451850874 | 5.00E-05 | 0.000216 | Protein of unknown function (DUF640) |
| AT4G31760 | 0.45186193 | 5.00E-05 | 0.000216 | Peroxidase superfamily protein |
| AT3G05910 | 0.451911238 | 5.00E-05 | 0.000216 | Pectinacetylesterase family protein |
| AT3G52740 | 0.451954235 | 5.00E-05 | 0.000216 | NA |
| AT5G50665 | 0.451989467 | 0.0003 | 0.00112 | NA |
| AT4G39920 | 0.452085424 | 5.00E-05 | 0.000216 | C-CAP/cofactor C-like domain-containing protein |
| AT1G71870 | 0.452516257 | 5.00E-05 | 0.000216 | MATE efflux family protein |
| AT5G16380 | 0.452729337 | 5.00E-05 | 0.000216 | Protein of unknown function, DUF538 |
| AT5G39530 | 0.453074328 | 5.00E-05 | 0.000216 | Protein of unknown function (DUF1997) |
| AT5G06250 | 0.453247195 | 5.00E-05 | 0.000216 | AP2/B3-like transcriptional factor family protein |
| AT5G41650 | 0.453343369 | 5.00E-05 | 0.000216 | Lactoylglutathione lyase / glyoxalase I family protein |
| AT4G22530 | 0.45438173 | 0.0003 | 0.00112 | S-adenosyl-L-methionine-dependent methyltransferases superfamily protein |
| AT1G04680 | 0.454460883 | 5.00E-05 | 0.000216 | Pectin lyase-like superfamily protein |
| AT4G20340 | 0.454770891 | 5.00E-05 | 0.000216 | Transcription factor TFIIE, alpha subunit |
| AT2G16400 | 0.45487805 | 5.00E-05 | 0.000216 | BEL1-like homeodomain 7 |
| AT5G02790 | 0.455179087 | 0.00025 | 0.000951 | Glutathione S-transferase family protein |
| AT1G59510 | 0.455687065 | 5.00E-05 | 0.000216 | Carbohydrate-binding protein |
| AT2G35740 | 0.455803037 | 0.0002 | 0.000777 | nositol transporter 3 |
| AT2G45400 | 0.455863185 | 5.00E-05 | 0.000216 | NAD(P)-binding Rossmann-fold superfamily protein |
| AT4G27150 | 0.456029057 | 5.00E-05 | 0.000216 | seed storage albumin 2 |
| AT1G02390 | 0.456051614 | 5.00E-05 | 0.000216 | glycerol-3-phosphate acyltransferase 2 |
| AT4G00750 | 0.45616911 | 0.01585 | 0.036311 | S-adenosyl-L-methionine-dependent methyltransferases superfamily protein |
| AT1G23052 | 0.456933084 | 5.00E-05 | 0.000216 | NA |
| AT3G15590 | 0.456959661 | 5.00E-05 | 0.000216 | Tetratricopeptide repeat (TPR)-like superfamily protein |
| AT1G13210 | 0.457606445 | 5.00E-05 | 0.000216 | autoinhibited Ca2+/ATPase II |
| AT3G29330 | 0.457804504 | 0.00495 | 0.013408 | NA |
| AT4G20780 | 0.457850759 | 0.0014 | 0.004449 | calmodulin like 42 |
| AT1G74470 | 0.457891987 | 5.00E-05 | 0.000216 | Pyridine nucleotide-disulphide oxidoreductase family protein |
| AT2G05220 | 0.458048114 | 5.00E-05 | 0.000216 | Ribosomal S17 family protein |
| AT5G20400 | 0.458424911 | 5.00E-05 | 0.000216 | 2-oxoglutarate (2OG) and Fe(II)-dependent oxygenase superfamily protein |
| AT2G47860 | 0.45850771 | 0.0011 | 0.003586 | Phototropic-responsive NPH3 family protein |
| AT3G43580 | 0.458573059 | 5.00E-05 | 0.000216 | Beta-galactosidase related protein |
| AT3G57040 | 0.459246783 | 5.00E-05 | 0.000216 | response regulator 9 |
| AT5G64480 | 0.459639853 | 5.00E-05 | 0.000216 | NA |
| AT5G27930 | 0.459881174 | 5.00E-05 | 0.000216 | Protein phosphatase 2C family protein |
| AT5G16200 | 0.459911702 | 0.018 | 0.040415 | 50S ribosomal protein-related |
| AT1G08930 | 0.460577528 | 5.00E-05 | 0.000216 | Major facilitator superfamily protein |
| AT2G14820 | 0.460640728 | 5.00E-05 | 0.000216 | Phototropic-responsive NPH3 family protein |
| AT5G17790 | 0.460885881 | 5.00E-05 | 0.000216 | zinc finger (Ran-binding) family protein |
| AT3G20395 | 0.460932597 | 5.00E-05 | 0.000216 | RING/U-box superfamily protein |
| AT5G59560 | 0.461033959 | 5.00E-05 | 0.000216 | sensitivity to red light reduced protein (SRR1) |
| AT1G12550 | 0.4613343 | 0.00295 | 0.008554 | D-isomer specific 2-hydroxyacid dehydrogenase family protein |
| AT1G74660 | 0.461335119 | 0.00055 | 0.001925 | mini zinc finger 1 |
| AT4G19830 | 0.461344211 | 5.00E-05 | 0.000216 | FKBP-like peptidyl-prolyl cis-trans isomerase family protein |
| AT3G17280 | 0.461704014 | 0.0191 | 0.042499 | F-box and associated interaction domains-containing protein |
| AT4G13920 | 0.461991024 | 5.00E-05 | 0.000216 | receptor like protein 50 |
| AT1G25275 | 0.462337978 | 5.00E-05 | 0.000216 | NA |
| AT2G26355 | 0.462341606 | 5.00E-05 | 0.000216 | other RNA |
| AT3G47570 | 0.462904518 | 5.00E-05 | 0.000216 | Leucine-rich repeat protein kinase family protein |
| AT3G11040 | 0.463373665 | 0.00045 | 0.001612 | Glycosyl hydrolase family 85 |
| AT1G16730 | 0.46368615 | 5.00E-05 | 0.000216 | unknown protein 6 |
| AT2G05580 | 0.463994032 | 5.00E-05 | 0.000216 | Glycine-rich protein family |
| AT5G45650 | 0.464272958 | 5.00E-05 | 0.000216 | subtilase family protein |
| AT1G02930 | 0.464355095 | 5.00E-05 | 0.000216 | glutathione S-transferase 6 |
| AT3G06360 | 0.46458287 | 0.0133 | 0.031324 | arabinogalactan protein 27 |
| AT1G21910 | 0.464775912 | 0.01875 | 0.041851 | Integrase-type DNA-binding superfamily protein |
| AT3G11280 | 0.465061251 | 0.01995 | 0.044102 | Duplicated homeodomain-like superfamily protein |
| AT5G07460 | 0.465259868 | 5.00E-05 | 0.000216 | peptidemethionine sulfoxide reductase 2 |
| AT5G37740 | 0.465446941 | 5.00E-05 | 0.000216 | Calcium-dependent lipid-binding (CaLB domain) family protein |
| AT5G06865 | 0.466264868 | 5.00E-05 | 0.000216 | other RNA |
| AT4G33070 | 0.466325691 | 5.00E-05 | 0.000216 | Thiamine pyrophosphate dependent pyruvate decarboxylase family protein |
| AT3G55800 | 0.466426609 | 5.00E-05 | 0.000216 | sedoheptulose-bisphosphatase |
| AT4G16060 | 0.466551444 | 5.00E-05 | 0.000216 | NA |
| AT4G36130 | 0.466633256 | 5.00E-05 | 0.000216 | Ribosomal protein L2 family |
| AT1G14345 | 0.466887612 | 0.0001 | 0.000412 | NAD(P)-linked oxidoreductase superfamily protein |
| AT3G55840 | 0.466950466 | 0.0001 | 0.000412 | Hs1pro-1 protein |
| AT1G12370 | 0.466959706 | 0.0192 | 0.042682 | photolyase 1 |
| AT1G69460 | 0.467272018 | 5.00E-05 | 0.000216 | emp24/gp25L/p24 family/GOLD family protein |
| AT3G25440 | 0.467400346 | 5.00E-05 | 0.000216 | RNA-binding CRS1 / YhbY (CRM) domain protein |
| AT5G59770 | 0.467645637 | 5.00E-05 | 0.000216 | Protein-tyrosine phosphatase-like, PTPLA |
| AT3G05570 | 0.468205739 | 5.00E-05 | 0.000216 | NA |
| AT2G03440 | 0.4682718 | 5.00E-05 | 0.000216 | nodulin-related protein 1 |
| AT1G61360 | 0.468322319 | 5.00E-05 | 0.000216 | S-locus lectin protein kinase family protein |
| AT3G44320 | 0.46885298 | 5.00E-05 | 0.000216 | nitrilase 3 |
| AT3G14150 | 0.469163169 | 5.00E-05 | 0.000216 | Aldolase-type TIM barrel family protein |
| AT1G35710 | 0.469331576 | 5.00E-05 | 0.000216 | Protein kinase family protein with leucine-rich repeat domain |
| AT4G20350 | 0.469339131 | 5.00E-05 | 0.000216 | oxidoreductases |
| AT2G17260 | 0.469802792 | 0.0001 | 0.000412 | glutamate receptor 2 |
| AT5G28770 | 0.470787269 | 5.00E-05 | 0.000216 | bZIP transcription factor family protein |
| AT1G01380 | 0.471793734 | 5.00E-05 | 0.000216 | Homeodomain-like superfamily protein |
| AT4G14965 | 0.471924599 | 5.00E-05 | 0.000216 | membrane-associated progesterone binding protein 4 |
| AT4G24040 | 0.472084075 | 5.00E-05 | 0.000216 | trehalase 1 |
| AT1G19450 | 0.472750861 | 5.00E-05 | 0.000216 | Major facilitator superfamily protein |
| AT4G08950 | 0.473135805 | 5.00E-05 | 0.000216 | Phosphate-responsive 1 family protein |
| AT2G29300 | 0.473471743 | 0.00015 | 0.000596 | NAD(P)-binding Rossmann-fold superfamily protein |
| AT2G29670 | 0.473672241 | 5.00E-05 | 0.000216 | Tetratricopeptide repeat (TPR)-like superfamily protein |
| AT2G16580 | 0.473700447 | 5.00E-05 | 0.000216 | SAUR-like auxin-responsive protein family |
| AT1G64950 | 0.474394483 | 5.00E-05 | 0.000216 | cytochrome P450, family 89, subfamily A, polypeptide 5 |
| AT3G27906 | 0.474470137 | 5.00E-05 | 0.000216 | NA |
| AT4G13930 | 0.474827776 | 5.00E-05 | 0.000216 | serine hydroxymethyltransferase 4 |
| AT1G61170 | 0.474954531 | 0.0021 | 0.006356 | NA |
| AT5G09660 | 0.476354343 | 5.00E-05 | 0.000216 | peroxisomal NAD-malate dehydrogenase 2 |
| AT1G32560 | 0.476635495 | 5.00E-05 | 0.000216 | Late embryogenesis abundant protein, group 1 protein |
| AT2G39415 | 0.477068378 | 5.00E-05 | 0.000216 | F-box family protein |
| AT1G59870 | 0.477234452 | 5.00E-05 | 0.000216 | ABC-2 and Plant PDR ABC-type transporter family protein |
| AT2G40170 | 0.477626049 | 5.00E-05 | 0.000216 | Stress induced protein |
| AT4G32980 | 0.477704515 | 0.0002 | 0.000777 | homeobox gene 1 |
| AT1G75450 | 0.477725005 | 5.00E-05 | 0.000216 | cytokinin oxidase 5 |
| AT5G44720 | 0.477870124 | 5.00E-05 | 0.000216 | Molybdenum cofactor sulfurase family protein |
| AT4G29030 | 0.478285837 | 5.00E-05 | 0.000216 | Putative membrane lipoprotein |
| AT5G13730 | 0.478362636 | 5.00E-05 | 0.000216 | sigma factor 4 |
| AT1G64720 | 0.478483912 | 5.00E-05 | 0.000216 | Polyketide cyclase/dehydrase and lipid transport superfamily protein |
| AT5G13630 | 0.478989294 | 5.00E-05 | 0.000216 | magnesium-chelatase subunit chlH, chloroplast, putative / Mg-protoporphyrin IX chelatase, putative (CHLH) |
| AT3G46440 | 0.479213133 | 5.00E-05 | 0.000216 | UDP-XYL synthase 5 |
| AT3G47980 | 0.479458338 | 5.00E-05 | 0.000216 | Integral membrane HPP family protein |
| AT1G75720 | 0.479545251 | 0.02115 | 0.046375 | Plant protein of unknown function (DUF827) |
| AT4G24380 | 0.479623215 | 5.00E-05 | 0.000216 | NA |
| AT2G34020 | 0.479897894 | 5.00E-05 | 0.000216 | Calcium-binding EF-hand family protein |
| AT5G26230 | 0.479974633 | 0.02835 | 0.059403 | NA |
| AT5G01881 | 0.480215394 | 0.00165 | 0.005138 | NA |
| AT4G24240 | 0.480327338 | 5.00E-05 | 0.000216 | WRKY DNA-binding protein 7 |
| AT3G48115 | 0.480405612 | 5.00E-05 | 0.000216 | other RNA |
| AT1G65540 | 0.480639501 | 5.00E-05 | 0.000216 | LETM1-like protein |
| AT5G40410 | 0.480731081 | 5.00E-05 | 0.000216 | Tetratricopeptide repeat (TPR)-like superfamily protein |
| AT1G29195 | 0.48100346 | 5.00E-05 | 0.000216 | NA |
| AT2G25520 | 0.4813213 | 5.00E-05 | 0.000216 | Drug/metabolite transporter superfamily protein |
| AT3G61820 | 0.481395131 | 5.00E-05 | 0.000216 | Eukaryotic aspartyl protease family protein |
| AT1G10650 | 0.481583647 | 5.00E-05 | 0.000216 | SBP (S-ribonuclease binding protein) family protein |
| AT5G16220 | 0.482493379 | 5.00E-05 | 0.000216 | Octicosapeptide/Phox/Bem1p family protein |
| AT1G17560 | 0.482632531 | 5.00E-05 | 0.000216 | Ribosomal protein L14p/L23e family protein |
| AT3G43600 | 0.482918011 | 5.00E-05 | 0.000216 | aldehyde oxidase 2 |
| AT5G23405 | 0.483040927 | 5.00E-05 | 0.000216 | HMG-box (high mobility group) DNA-binding family protein |
| AT2G27970 | 0.48322711 | 5.00E-05 | 0.000216 | CDK-subunit 2 |
| AT4G11600 | 0.483311335 | 5.00E-05 | 0.000216 | glutathione peroxidase 6 |
| AT1G73500 | 0.483494428 | 5.00E-05 | 0.000216 | MAP kinase kinase 9 |
| AT4G24480 | 0.483948543 | 5.00E-05 | 0.000216 | Protein kinase superfamily protein |
| AT5G51460 | 0.484090347 | 5.00E-05 | 0.000216 | Haloacid dehalogenase-like hydrolase (HAD) superfamily protein |
| AT1G60989 | 0.484124025 | 0.0015 | 0.004727 | SCR-like 7 |
| AT3G13040 | 0.484296423 | 5.00E-05 | 0.000216 | myb-like HTH transcriptional regulator family protein |
| AT3G18280 | 0.484541153 | 5.00E-05 | 0.000216 | Bifunctional inhibitor/lipid-transfer protein/seed storage 2S albumin superfamily protein |
| AT2G43510 | 0.484895262 | 5.00E-05 | 0.000216 | trypsin inhibitor protein 1 |
| AT4G28706 | 0.484910205 | 5.00E-05 | 0.000216 | pfkB-like carbohydrate kinase family protein |
| AT4G12910 | 0.484932453 | 5.00E-05 | 0.000216 | serine carboxypeptidase-like 20 |
| AT1G69520 | 0.48566705 | 0.0001 | 0.000412 | S-adenosyl-L-methionine-dependent methyltransferases superfamily protein |
| AT5G43850 | 0.48569875 | 5.00E-05 | 0.000216 | RmlC-like cupins superfamily protein |
| AT5G41860 | 0.485818792 | 0.033 | 0.067397 | NA |
| AT4G27870 | 0.486364191 | 5.00E-05 | 0.000216 | Vacuolar iron transporter (VIT) family protein |
| AT5G22690 | 0.486669349 | 5.00E-05 | 0.000216 | Disease resistance protein (TIR-NBS-LRR class) family |
| AT3G49910 | 0.486737387 | 5.00E-05 | 0.000216 | Translation protein SH3-like family protein |
| AT1G26560 | 0.486787471 | 5.00E-05 | 0.000216 | beta glucosidase 40 |
| AT2G47780 | 0.487080322 | 5.00E-05 | 0.000216 | Rubber elongation factor protein (REF) |
| AT5G44210 | 0.48719785 | 5.00E-05 | 0.000216 | erf domain protein 9 |
| AT1G64500 | 0.487234509 | 0.0002 | 0.000777 | Glutaredoxin family protein |
| AT4G03210 | 0.487762748 | 5.00E-05 | 0.000216 | xyloglucan endotransglucosylase/hydrolase 9 |
| AT1G68710 | 0.488032951 | 5.00E-05 | 0.000216 | ATPase E1-E2 type family protein / haloacid dehalogenase-like hydrolase family protein |
| AT5G09440 | 0.488093613 | 5.00E-05 | 0.000216 | EXORDIUM like 4 |
| AT3G26430 | 0.48923613 | 0.0073 | 0.018734 | GDSL-like Lipase/Acylhydrolase superfamily protein |
| AT5G14700 | 0.489343964 | 0.00735 | 0.018837 | NAD(P)-binding Rossmann-fold superfamily protein |
| AT2G02960 | 0.489380403 | 5.00E-05 | 0.000216 | RING/FYVE/PHD zinc finger superfamily protein |
| AT4G10490 | 0.489399773 | 0.00025 | 0.000951 | 2-oxoglutarate (2OG) and Fe(II)-dependent oxygenase superfamily protein |
| AT5G05365 | 0.489545689 | 0.019 | 0.042309 | Heavy metal transport/detoxification superfamily protein |
| AT3G14172 | 0.490180809 | 5.00E-05 | 0.000216 | NA |
| AT1G21310 | 0.490708154 | 5.00E-05 | 0.000216 | extensin 3 |
| AT4G36052 | 0.490843457 | 0.00055 | 0.001925 | other RNA |
| AT5G24610 | 0.490914801 | 5.00E-05 | 0.000216 | NA |
| AT5G60250 | 0.491574161 | 5.00E-05 | 0.000216 | zinc finger (C3HC4-type RING finger) family protein |
| AT5G62520 | 0.491624313 | 5.00E-05 | 0.000216 | similar to RCD one 5 |
| AT2G24610 | 0.49175799 | 5.00E-05 | 0.000216 | cyclic nucleotide-gated channel 14 |
| AT2G40380 | 0.491851517 | 5.00E-05 | 0.000216 | prenylated RAB acceptor 1.B2 |
| AT5G01840 | 0.492253383 | 0.02465 | 0.052733 | ovate family protein 1 |
| AT1G26800 | 0.49238646 | 5.00E-05 | 0.000216 | RING/U-box superfamily protein |
| AT5G60460 | 0.492422556 | 5.00E-05 | 0.000216 | Preprotein translocase Sec, Sec61-beta subunit protein |
| AT1G77120 | 0.492458374 | 5.00E-05 | 0.000216 | alcohol dehydrogenase 1 |
| AT5G48060 | 0.492592848 | 5.00E-05 | 0.000216 | C2 calcium/lipid-binding plant phosphoribosyltransferase family protein |
| AT2G26040 | 0.493144113 | 5.00E-05 | 0.000216 | PYR1-like 2 |
| AT1G67740 | 0.49326043 | 5.00E-05 | 0.000216 | photosystem II BY |
| AT1G74790 | 0.493562059 | 5.00E-05 | 0.000216 | catalytics |
| AT5G46730 | 0.493767605 | 0.0342 | 0.069423 | glycine-rich protein |
| AT1G17870 | 0.493971305 | 5.00E-05 | 0.000216 | ethylene-dependent gravitropism-deficient and yellow-green-like 3 |
| AT1G05950 | 0.494652362 | 0.00165 | 0.005138 | NA |
| AT2G20260 | 0.494675248 | 0.0021 | 0.006356 | photosystem I subunit E-2 |
| AT1G26290 | 0.494879673 | 0.0065 | 0.016954 | NA |
| AT5G60890 | 0.495300105 | 5.00E-05 | 0.000216 | myb domain protein 34 |
| AT1G03890 | 0.495388197 | 0.0003 | 0.00112 | RmlC-like cupins superfamily protein |
| AT1G25560 | 0.495403175 | 0.00035 | 0.001288 | AP2/B3 transcription factor family protein |
| AT1G43770 | 0.495603686 | 5.00E-05 | 0.000216 | RING/FYVE/PHD zinc finger superfamily protein |
| AT4G12880 | 0.495771807 | 5.00E-05 | 0.000216 | early nodulin-like protein 19 |
| AT3G60660 | 0.495801236 | 0.0002 | 0.000777 | NA |
| AT2G44160 | 0.496207282 | 5.00E-05 | 0.000216 | methylenetetrahydrofolate reductase 2 |
| AT2G02400 | 0.49635876 | 5.00E-05 | 0.000216 | NAD(P)-binding Rossmann-fold superfamily protein |
| AT5G25190 | 0.496376588 | 5.00E-05 | 0.000216 | Integrase-type DNA-binding superfamily protein |
| AT3G63200 | 0.496472168 | 5.00E-05 | 0.000216 | PATATIN-like protein 9 |
| AT4G13040 | 0.496580388 | 5.00E-05 | 0.000216 | Integrase-type DNA-binding superfamily protein |
| AT2G37610 | 0.497651858 | 0.04405 | 0.086054 | NA |
| AT4G21740 | 0.497708925 | 0.0002 | 0.000777 | NA |
| AT1G66250 | 0.497829146 | 5.00E-05 | 0.000216 | O-Glycosyl hydrolases family 17 protein |
| AT3G55820 | 0.497859947 | 0.01 | 0.024543 | Fasciclin-like arabinogalactan family protein |
| AT5G12020 | 0.49834545 | 5.00E-05 | 0.000216 | 17.6 kDa class II heat shock protein |
| AT4G12040 | 0.498546309 | 5.00E-05 | 0.000216 | A20/AN1-like zinc finger family protein |
| AT3G46230 | 0.498665794 | 5.00E-05 | 0.000216 | heat shock protein 17.4 |
| AT1G12805 | 0.49875317 | 5.00E-05 | 0.000216 | nucleotide binding |
| AT1G60610 | 0.498976626 | 5.00E-05 | 0.000216 | SBP (S-ribonuclease binding protein) family protein |
| AT3G22850 | 0.499228384 | 5.00E-05 | 0.000216 | Aluminium induced protein with YGL and LRDR motifs |
| AT1G80180 | 0.499584565 | 5.00E-05 | 0.000216 | NA |
| AT1G62050 | 0.4996217 | 5.00E-05 | 0.000216 | Ankyrin repeat family protein |
| AT3G43960 | 0.499833511 | 5.00E-05 | 0.000216 | Cysteine proteinases superfamily protein |

**Supplemental Table S10**. The genes whose expression is up-regulated in *abi5-1* seeds.

| **Gene_ID** | **Fold change (*abi5-1*/Col-0)** | **P-value** | **FDR** | **Gene description** |
| --- | --- | --- | --- | --- |
| AT3G18770 | 2.000201 | 0.00005 | 0.000216 | Autophagy-related protein 13 |
| AT1G72540 | 2.0015 | 0.0063 | 0.016503 | Protein kinase superfamily protein |
| AT3G05640 | 2.001902 | 0.00005 | 0.000216 | Protein phosphatase 2C family protein |
| AT1G55152 | 2.002024 | 0.00005 | 0.000216 | NA |
| AT3G02170 | 2.002144 | 0.00005 | 0.000216 | longifolia2 |
| AT2G46070 | 2.008218 | 0.00005 | 0.000216 | mitogen-activated protein kinase 12 |
| AT3G14470 | 2.009563 | 0.00005 | 0.000216 | NB-ARC domain-containing disease resistance protein |
| AT1G14200 | 2.010817 | 0.00005 | 0.000216 | RING/U-box superfamily protein |
| AT2G18570 | 2.013033 | 0.00005 | 0.000216 | UDP-Glycosyltransferase superfamily protein |
| AT2G38820 | 2.015828 | 0.00005 | 0.000216 | Protein of unknown function (DUF506) |
| AT5G02070 | 2.020181 | 0.00005 | 0.000216 | Protein kinase family protein |
| AT3G17225 | 2.022686 | 0.0013 | 0.004171 | Plant invertase/pectin methylesterase inhibitor superfamily protein |
| AT3G62890 | 2.02335 | 0.00005 | 0.000216 | Pentatricopeptide repeat (PPR) superfamily protein |
| AT4G19420 | 2.024163 | 0.00005 | 0.000216 | Pectinacetylesterase family protein |
| AT5G40382 | 2.026046 | 0.0061 | 0.016061 | Cytochrome c oxidase subunit Vc family protein |
| AT3G02610 | 2.028608 | 0.00005 | 0.000216 | Plant stearoyl-acyl-carrier-protein desaturase family protein |
| AT1G75490 | 2.029161 | 0.00005 | 0.000216 | Integrase-type DNA-binding superfamily protein |
| AT1G15380 | 2.032784 | 0.04915 | 0.094054 | Lactoylglutathione lyase / glyoxalase I family protein |
| AT3G59940 | 2.036742 | 0.00005 | 0.000216 | Galactose oxidase/kelch repeat superfamily protein |
| AT3G62920 | 2.037504 | 0.00005 | 0.000216 | NA |
| AT5G58690 | 2.038497 | 0.0001 | 0.000412 | phosphatidylinositol-speciwc phospholipase C5 |
| AT4G10845 | 2.038732 | 0.0027 | 0.007914 | NA |
| AT2G26570 | 2.040621 | 0.00005 | 0.000216 | Plant protein of unknown function (DUF827) |
| AT2G15880 | 2.042676 | 0.00005 | 0.000216 | Leucine-rich repeat (LRR) family protein |
| AT5G01300 | 2.043602 | 0.00005 | 0.000216 | PEBP (phosphatidylethanolamine-binding protein) family protein |
| AT1G10050 | 2.043914 | 0.00005 | 0.000216 | glycosyl hydrolase family 10 protein / carbohydrate-binding domain-containing protein |
| AT4G30850 | 2.044094 | 0.00005 | 0.000216 | heptahelical transmembrane protein2 |
| AT5G64510 | 2.047883 | 0.00005 | 0.000216 | NA |
| AT3G10130 | 2.048205 | 0.00005 | 0.000216 | SOUL heme-binding family protein |
| AT2G17300 | 2.049498 | 0.02475 | 0.052914 | NA |
| AT5G14960 | 2.05497 | 0.00005 | 0.000216 | DP-E2F-like 2 |
| AT1G69440 | 2.056567 | 0.00005 | 0.000216 | Argonaute family protein |
| AT2G07000 | 2.056826 | 0.00005 | 0.000216 | NA |
| AT5G49500 | 2.058179 | 0.00005 | 0.000216 | Signal recognition particle, SRP54 subunit protein |
| AT3G57020 | 2.060668 | 0.00005 | 0.000216 | Calcium-dependent phosphotriesterase superfamily protein |
| AT5G54095 | 2.060985 | 0.00005 | 0.000216 | NA |
| AT1G29560 | 2.061051 | 0.00005 | 0.000216 | Zinc finger C-x8-C-x5-C-x3-H type family protein |
| AT1G03440 | 2.061863 | 0.00005 | 0.000216 | Leucine-rich repeat (LRR) family protein |
| AT5G38590 | 2.063496 | 0.00005 | 0.000216 | F-box/RNI-like/FBD-like domains-containing protein |
| AT3G01470 | 2.064718 | 0.00005 | 0.000216 | homeobox 1 |
| AT2G06005 | 2.065209 | 0.00005 | 0.000216 | FRIGIDA interacting protein 1 |
| AT2G21430 | 2.067346 | 0.00005 | 0.000216 | Papain family cysteine protease |
| AT1G32870 | 2.068589 | 0.00005 | 0.000216 | NAC domain protein 13 |
| AT5G25610 | 2.06865 | 0.00005 | 0.000216 | BURP domain-containing protein |
| AT3G59490 | 2.06977 | 0.00005 | 0.000216 | NA |
| AT5G19250 | 2.070619 | 0.00535 | 0.014339 | Glycoprotein membrane precursor GPI-anchored |
| AT1G13420 | 2.070958 | 0.013 | 0.030728 | sulfotransferase 4B |
| AT5G61190 | 2.071807 | 0.00005 | 0.000216 | putative endonuclease or glycosyl hydrolase with C2H2-type zinc finger domain |
| AT5G27920 | 2.07564 | 0.00005 | 0.000216 | F-box family protein |
| AT3G55610 | 2.076228 | 0.00005 | 0.000216 | delta 1-pyrroline-5-carboxylate synthase 2 |
| AT1G13140 | 2.079243 | 0.0001 | 0.000412 | cytochrome P450, family 86, subfamily C, polypeptide 3 |
| AT2G24180 | 2.080002 | 0.00005 | 0.000216 | cytochrome p450 71b6 |
| AT1G01225 | 2.0801 | 0.0026 | 0.007668 | NC domain-containing protein-related |
| AT3G01100 | 2.081076 | 0.00005 | 0.000216 | hypothetical protein 1 |
| AT4G12120 | 2.082016 | 0.00005 | 0.000216 | Sec1/munc18-like (SM) proteins superfamily |
| AT2G34440 | 2.085565 | 0.04405 | 0.086054 | AGAMOUS-like 29 |
| AT3G12203 | 2.08946 | 0.00055 | 0.001925 | serine carboxypeptidase-like 17 |
| AT5G44670 | 2.092522 | 0.00005 | 0.000216 | Domain of unknown function (DUF23) |
| AT5G57050 | 2.092819 | 0.00005 | 0.000216 | Protein phosphatase 2C family protein |
| AT4G34280 | 2.095064 | 0.00005 | 0.000216 | transducin family protein / WD-40 repeat family protein |
| AT1G09080 | 2.098134 | 0.0041 | 0.011381 | Heat shock protein 70 (Hsp 70) family protein |
| AT4G28720 | 2.099195 | 0.0063 | 0.016503 | Flavin-binding monooxygenase family protein |
| AT3G60490 | 2.100209 | 0.00005 | 0.000216 | Integrase-type DNA-binding superfamily protein |
| AT1G17830 | 2.100666 | 0.00005 | 0.000216 | Protein of unknown function (DUF789) |
| AT4G30630 | 2.10387 | 0.00005 | 0.000216 | NA |
| AT2G38780 | 2.103904 | 0.00005 | 0.000216 | NA |
| AT3G56960 | 2.104405 | 0.00005 | 0.000216 | phosphatidyl inositol monophosphate 5 kinase 4 |
| AT1G43675 | 2.106124 | 0.00005 | 0.000216 | transposable element gene |
| AT1G52570 | 2.107047 | 0.00005 | 0.000216 | phospholipase D alpha 2 |
| AT1G64142 | 2.107181 | 0.00005 | 0.000216 | conserved peptide upstream open reading frame 23 |
| AT5G55750 | 2.107973 | 0.0002 | 0.000777 | hydroxyproline-rich glycoprotein family protein |
| AT5G15870 | 2.108764 | 0.00005 | 0.000216 | glycosyl hydrolase family 81 protein |
| AT3G21010 | 2.109984 | 0.00005 | 0.000216 | transposable element gene |
| AT3G26510 | 2.111141 | 0.00005 | 0.000216 | Octicosapeptide/Phox/Bem1p family protein |
| AT1G67920 | 2.113325 | 0.00015 | 0.000596 | NA |
| AT5G09980 | 2.113519 | 0.00005 | 0.000216 | elicitor peptide 4 precursor |
| AT2G18700 | 2.113934 | 0.00005 | 0.000216 | trehalose phosphatase/synthase 11 |
| AT4G29230 | 2.113953 | 0.00005 | 0.000216 | NAC domain containing protein 75 |
| AT5G04500 | 2.119599 | 0.00005 | 0.000216 | glycosyltransferase family protein 47 |
| AT5G46640 | 2.124655 | 0.00005 | 0.000216 | AT hook motif DNA-binding family protein |
| AT2G41300 | 2.125745 | 0.00005 | 0.000216 | strictosidine synthase-like 1 |
| AT2G36220 | 2.125805 | 0.00005 | 0.000216 | NA |
| AT1G36060 | 2.127387 | 0.00005 | 0.000216 | Integrase-type DNA-binding superfamily protein |
| AT5G49690 | 2.127493 | 0.00005 | 0.000216 | UDP-Glycosyltransferase superfamily protein |
| AT1G80540 | 2.129649 | 0.00005 | 0.000216 | NA |
| AT3G46600 | 2.130573 | 0.00005 | 0.000216 | GRAS family transcription factor |
| AT3G14460 | 2.130642 | 0.00005 | 0.000216 | LRR and NB-ARC domains-containing disease resistance protein |
| AT5G48230 | 2.131299 | 0.00005 | 0.000216 | acetoacetyl-CoA thiolase 2 |
| AT5G65550 | 2.135341 | 0.00005 | 0.000216 | UDP-Glycosyltransferase superfamily protein |
| AT1G53180 | 2.138027 | 0.00005 | 0.000216 | NA |
| AT2G15580 | 2.138206 | 0.00005 | 0.000216 | RING/U-box superfamily protein |
| AT5G10300 | 2.141143 | 0.00005 | 0.000216 | methyl esterase 5 |
| AT1G64270 | 2.141312 | 0.00005 | 0.000216 | transposable element gene |
| AT1G71990 | 2.141377 | 0.00005 | 0.000216 | fucosyltransferase 13 |
| AT2G02230 | 2.145303 | 0.00005 | 0.000216 | phloem protein 2-B1 |
| AT1G80570 | 2.145922 | 0.00005 | 0.000216 | RNI-like superfamily protein |
| AT1G23550 | 2.148163 | 0.00005 | 0.000216 | similar to RCD one 2 |
| AT2G14520 | 2.149062 | 0.00005 | 0.000216 | CBS domain-containing protein with a domain of unknown function (DUF21) |
| AT5G64060 | 2.149625 | 0.00005 | 0.000216 | NAC domain containing protein 103 |
| AT5G67520 | 2.153716 | 0.00005 | 0.000216 | adenosine-5'-phosphosulfate (APS) kinase 4 |
| AT1G33980 | 2.154077 | 0.00005 | 0.000216 | Smg-4/UPF3 family protein |
| AT1G52920 | 2.155489 | 0.00005 | 0.000216 | G protein coupled receptor |
| AT1G30860 | 2.157293 | 0.00005 | 0.000216 | RING/U-box superfamily protein |
| AT5G02080 | 2.162084 | 0.00005 | 0.000216 | DNA / pantothenate metabolism flavoprotein |
| AT3G28750 | 2.168402 | 0.0018 | 0.00555 | NA |
| AT1G47560 | 2.169465 | 0.00005 | 0.000216 | exocyst complex component sec3B |
| AT4G33940 | 2.172119 | 0.00005 | 0.000216 | RING/U-box superfamily protein |
| AT4G33260 | 2.174479 | 0.00055 | 0.001925 | Transducin family protein / WD-40 repeat family protein |
| AT2G38905 | 2.177128 | 0.00005 | 0.000216 | Low temperature and salt responsive protein family |
| AT1G61490 | 2.179449 | 0.00005 | 0.000216 | S-locus lectin protein kinase family protein |
| AT4G11840 | 2.180393 | 0.00005 | 0.000216 | phospholipase D gamma 3 |
| AT1G22430 | 2.182544 | 0.00005 | 0.000216 | GroES-like zinc-binding dehydrogenase family protein |
| AT2G41475 | 2.183545 | 0.00005 | 0.000216 | Embryo-specific protein 3, (ATS3) |
| AT3G49900 | 2.186793 | 0.00375 | 0.010538 | Phototropic-responsive NPH3 family protein |
| AT2G01400 | 2.18879 | 0.00005 | 0.000216 | NA |
| AT4G26590 | 2.190787 | 0.00005 | 0.000216 | oligopeptide transporter 5 |
| AT3G27150 | 2.191437 | 0.00265 | 0.007791 | Galactose oxidase/kelch repeat superfamily protein |
| AT5G03240 | 2.19563 | 0.00005 | 0.000216 | polyubiquitin 3 |
| AT1G64310 | 2.195878 | 0.00035 | 0.001288 | Tetratricopeptide repeat (TPR)-like superfamily protein |
| AT1G04570 | 2.19604 | 0.00005 | 0.000216 | Major facilitator superfamily protein |
| AT4G23410 | 2.197453 | 0.02135 | 0.046712 | tetraspanin5 |
| AT2G30140 | 2.199078 | 0.00005 | 0.000216 | UDP-Glycosyltransferase superfamily protein |
| AT1G19530 | 2.202851 | 0.00005 | 0.000216 | NA |
| AT2G34360 | 2.203321 | 0.0019 | 0.005818 | MATE efflux family protein |
| AT4G36010 | 2.203695 | 0.00005 | 0.000216 | Pathogenesis-related thaumatin superfamily protein |
| AT4G16900 | 2.203847 | 0.00005 | 0.000216 | Disease resistance protein (TIR-NBS-LRR class) family |
| AT1G71330 | 2.204822 | 0.00005 | 0.000216 | non-intrinsic ABC protein 5 |
| AT2G07695 | 2.205288 | 0.01545 | 0.035518 | Cytochrome C oxidase subunit II-like, transmembrane domain |
| AT1G77410 | 2.211804 | 0.00005 | 0.000216 | beta-galactosidase 16 |
| AT2G36640 | 2.216232 | 0.00005 | 0.000216 | embryonic cell protein 63 |
| AT2G33520 | 2.21728 | 0.00005 | 0.000216 | NA |
| AT5G13220 | 2.217667 | 0.00005 | 0.000216 | jasmonate-zim-domain protein 10 |
| AT5G67600 | 2.218022 | 0.00005 | 0.000216 | NA |
| AT5G42460 | 2.21914 | 0.00005 | 0.000216 | F-box and associated interaction domains-containing protein |
| AT3G11340 | 2.221543 | 0.00005 | 0.000216 | UDP-Glycosyltransferase superfamily protein |
| AT5G24155 | 2.222953 | 0.0317 | 0.065234 | FAD/NAD(P)-binding oxidoreductase family protein |
| AT4G10930 | 2.224269 | 0.00005 | 0.000216 | NA |
| AT3G18610 | 2.224822 | 0.00005 | 0.000216 | nucleolin like 2 |
| AT3G60670 | 2.225936 | 0.03155 | 0.064973 | PLATZ transcription factor family protein |
| AT5G54000 | 2.232318 | 0.00165 | 0.005138 | 2-oxoglutarate (2OG) and Fe(II)-dependent oxygenase superfamily protein |
| AT5G59590 | 2.238143 | 0.00005 | 0.000216 | UDP-glucosyl transferase 76E2 |
| AT2G46530 | 2.238335 | 0.00005 | 0.000216 | auxin response factor 11 |
| AT3G18145 | 2.238952 | 0.00005 | 0.000216 | NA |
| AT5G39610 | 2.238978 | 0.00005 | 0.000216 | NAC domain containing protein 6 |
| AT3G11080 | 2.239067 | 0.00005 | 0.000216 | receptor like protein 35 |
| AT1G71140 | 2.242646 | 0.00005 | 0.000216 | MATE efflux family protein |
| AT3G51470 | 2.243742 | 0.0001 | 0.000412 | Protein phosphatase 2C family protein |
| AT2G02370 | 2.243827 | 0.00005 | 0.000216 | SNARE associated Golgi protein family |
| AT5G08460 | 2.249313 | 0.0008 | 0.0027 | GDSL-like Lipase/Acylhydrolase superfamily protein |
| AT5G37072 | 2.252198 | 0.0379 | 0.075645 | NA |
| AT1G78955 | 2.254927 | 0.00005 | 0.000216 | camelliol C synthase 1 |
| AT4G33490 | 2.256005 | 0.03065 | 0.063357 | Eukaryotic aspartyl protease family protein |
| AT5G40570 | 2.261477 | 0.01785 | 0.040126 | Surfeit locus protein 2 (SURF2) |
| AT1G74170 | 2.261818 | 0.00005 | 0.000216 | receptor like protein 13 |
| AT5G06370 | 2.263401 | 0.00005 | 0.000216 | NC domain-containing protein-related |
| AT3G05140 | 2.263736 | 0.00005 | 0.000216 | ROP binding protein kinases 2 |
| AT5G66110 | 2.26665 | 0.00005 | 0.000216 | Heavy metal transport/detoxification superfamily protein |
| AT2G28560 | 2.271197 | 0.00045 | 0.001612 | DNA repair (Rad51) family protein |
| AT4G34810 | 2.271732 | 0.0002 | 0.000777 | SAUR-like auxin-responsive protein family |
| AT1G28260 | 2.272933 | 0.00005 | 0.000216 | Telomerase activating protein Est1 |
| AT3G18980 | 2.275477 | 0.00005 | 0.000216 | EIN2 targeting protein1 |
| AT4G24015 | 2.279562 | 0.0049 | 0.013287 | RING/U-box superfamily protein |
| AT3G52160 | 2.27985 | 0.00055 | 0.001925 | 3-ketoacyl-CoA synthase 15 |
| AT2G31585 | 2.284382 | 0.00005 | 0.000216 | other RNA |
| AT3G07420 | 2.285573 | 0.00005 | 0.000216 | asparaginyl-tRNA synthetase 2 |
| AT1G65000 | 2.291521 | 0.00005 | 0.000216 | NA |
| AT5G10946 | 2.291829 | 0.00005 | 0.000216 | NA |
| AT3G45880 | 2.2926 | 0.00005 | 0.000216 | 2-oxoglutarate (2OG) and Fe(II)-dependent oxygenase superfamily protein |
| AT2G17036 | 2.293158 | 0.00005 | 0.000216 | F-box family protein with a domain of unknown function (DUF295) |
| AT3G47780 | 2.298139 | 0.00005 | 0.000216 | ABC2 homolog 6 |
| AT1G33612 | 2.298718 | 0.00005 | 0.000216 | Leucine-rich repeat (LRR) family protein |
| AT3G12730 | 2.308723 | 0.01035 | 0.02526 | Homeodomain-like superfamily protein |
| AT1G19650 | 2.310685 | 0.00005 | 0.000216 | Sec14p-like phosphatidylinositol transfer family protein |
| AT2G18600 | 2.311649 | 0.00005 | 0.000216 | Ubiquitin-conjugating enzyme family protein |
| AT4G21070 | 2.312163 | 0.00005 | 0.000216 | breast cancer susceptibility1 |
| AT4G10390 | 2.314872 | 0.00005 | 0.000216 | Protein kinase superfamily protein |
| AT2G22080 | 2.318271 | 0.00005 | 0.000216 | NA |
| AT4G21120 | 2.318281 | 0.00005 | 0.000216 | amino acid transporter 1 |
| AT1G48405 | 2.322252 | 0.01275 | 0.030267 | Kinase interacting (KIP1-like) family protein |
| AT2G15590 | 2.322642 | 0.02625 | 0.055597 | Protein of unknown function (DUF1685) |
| AT1G34260 | 2.333827 | 0.00005 | 0.000216 | FORMS APLOID AND BINUCLEATE CELLS 1A |
| AT5G05300 | 2.337846 | 0.022 | 0.047876 | NA |
| AT2G28490 | 2.338027 | 0.00005 | 0.000216 | RmlC-like cupins superfamily protein |
| AT4G16690 | 2.3388 | 0.00005 | 0.000216 | methyl esterase 16 |
| AT5G06690 | 2.344307 | 0.00005 | 0.000216 | WCRKC thioredoxin 1 |
| AT1G63380 | 2.346907 | 0.0088 | 0.02199 | NAD(P)-binding Rossmann-fold superfamily protein |
| AT3G25495 | 2.348745 | 0.00005 | 0.000216 | NA |
| AT3G17520 | 2.35224 | 0.00005 | 0.000216 | Late embryogenesis abundant protein (LEA) family protein |
| AT1G07645 | 2.353144 | 0.00005 | 0.000216 | dessication-induced 1VOC superfamily protein |
| AT5G06790 | 2.356217 | 0.02495 | 0.053261 | NA |
| AT1G12600 | 2.363663 | 0.00005 | 0.000216 | UDP-N-acetylglucosamine (UAA) transporter family |
| AT2G16850 | 2.364733 | 0.00005 | 0.000216 | plasma membrane intrinsic protein 2;8 |
| AT1G67830 | 2.367954 | 0.00005 | 0.000216 | alpha-fucosidase 1 |
| AT2G05310 | 2.370173 | 0.00005 | 0.000216 | NA |
| AT1G30590 | 2.371037 | 0.00005 | 0.000216 | RNA polymerase I specific transcription initiation factor RRN3 protein |
| AT3G55630 | 2.375314 | 0.0008 | 0.0027 | DHFS-FPGS homolog D |
| AT3G07350 | 2.377182 | 0.0006 | 0.002077 | Protein of unknown function (DUF506) |
| AT3G56080 | 2.38068 | 0.00005 | 0.000216 | S-adenosyl-L-methionine-dependent methyltransferases superfamily protein |
| AT3G53230 | 2.383197 | 0.00005 | 0.000216 | ATPase, AAA-type, CDC48 protein |
| AT3G08660 | 2.385483 | 0.003 | 0.008677 | Phototropic-responsive NPH3 family protein |
| AT1G76590 | 2.390681 | 0.00005 | 0.000216 | PLATZ transcription factor family protein |
| AT2G28105 | 2.401672 | 0.0124 | 0.029535 | NA |
| AT2G35660 | 2.413876 | 0.0003 | 0.00112 | FAD/NAD(P)-binding oxidoreductase family protein |
| AT2G24030 | 2.414005 | 0.001 | 0.003297 | zinc ion binding;nucleic acid binding |
| AT1G09460 | 2.418278 | 0.00005 | 0.000216 | Carbohydrate-binding X8 domain superfamily protein |
| AT4G37490 | 2.420529 | 0.00005 | 0.000216 | CYCLIN B1;1 |
| AT5G22460 | 2.423569 | 0.00005 | 0.000216 | alpha/beta-Hydrolases superfamily protein |
| AT5G18450 | 2.424843 | 0.00005 | 0.000216 | Integrase-type DNA-binding superfamily protein |
| AT3G23560 | 2.425504 | 0.00005 | 0.000216 | MATE efflux family protein |
| AT1G52690 | 2.429306 | 0.00005 | 0.000216 | Late embryogenesis abundant protein (LEA) family protein |
| AT5G45690 | 2.433826 | 0.00005 | 0.000216 | Protein of unknown function (DUF1264) |
| AT3G14550 | 2.434629 | 0.00005 | 0.000216 | geranylgeranyl pyrophosphate synthase 3 |
| AT2G12190 | 2.43584 | 0.00005 | 0.000216 | Cytochrome P450 superfamily protein |
| AT1G33050 | 2.436316 | 0.00005 | 0.000216 | NA |
| AT3G16980 | 2.437979 | 0.00005 | 0.000216 | RNA polymerases M/15 Kd subunit |
| AT5G64080 | 2.443346 | 0.00005 | 0.000216 | Bifunctional inhibitor/lipid-transfer protein/seed storage 2S albumin superfamily protein |
| AT4G36830 | 2.445878 | 0.0154 | 0.035437 | GNS1/SUR4 membrane protein family |
| AT2G21820 | 2.455957 | 0.00005 | 0.000216 | NA |
| AT2G26640 | 2.4571 | 0.01205 | 0.028794 | 3-ketoacyl-CoA synthase 11 |
| AT4G02280 | 2.459047 | 0.00005 | 0.000216 | sucrose synthase 3 |
| AT1G54870 | 2.462878 | 0.00005 | 0.000216 | NAD(P)-binding Rossmann-fold superfamily protein |
| AT1G27565 | 2.465193 | 0.0201 | 0.044393 | NA |
| AT1G05820 | 2.472333 | 0.00385 | 0.010775 | SIGNAL PEPTIDE PEPTIDASE-LIKE 5 |
| AT2G46660 | 2.472495 | 0.00005 | 0.000216 | cytochrome P450, family 78, subfamily A, polypeptide 6 |
| AT2G42980 | 2.473967 | 0.00005 | 0.000216 | Eukaryotic aspartyl protease family protein |
| AT1G04880 | 2.479707 | 0.0058 | 0.015378 | HMG (high mobility group) box protein with ARID/BRIGHT DNA-binding domain |
| AT4G39070 | 2.48177 | 0.00005 | 0.000216 | B-box zinc finger family protein |
| AT1G50280 | 2.487822 | 0.0022 | 0.006618 | Phototropic-responsive NPH3 family protein |
| AT3G23870 | 2.490683 | 0.00005 | 0.000216 | Protein of unknown function (DUF803) |
| AT5G07990 | 2.492261 | 0.00005 | 0.000216 | Cytochrome P450 superfamily protein |
| AT5G22540 | 2.494864 | 0.00175 | 0.005406 | Plant protein of unknown function (DUF247) |
| AT5G62470 | 2.496364 | 0.00005 | 0.000216 | myb domain protein 96 |
| AT3G54940 | 2.5003 | 0.00005 | 0.000216 | Papain family cysteine protease |
| AT1G61370 | 2.501954 | 0.00005 | 0.000216 | S-locus lectin protein kinase family protein |
| AT5G53200 | 2.503118 | 0.00005 | 0.000216 | Homeodomain-like superfamily protein |
| AT3G06630 | 2.5033 | 0.00005 | 0.000216 | protein kinase family protein |
| AT5G46110 | 2.504287 | 0.00005 | 0.000216 | Glucose-6-phosphate/phosphate translocator-related |
| AT3G52460 | 2.506205 | 0.00695 | 0.017957 | hydroxyproline-rich glycoprotein family protein |
| AT5G21105 | 2.508476 | 0.00005 | 0.000216 | Plant L-ascorbate oxidase |
| AT5G40390 | 2.510083 | 0.00005 | 0.000216 | Raffinose synthase family protein |
| AT3G19920 | 2.511675 | 0.00005 | 0.000216 | NA |
| AT1G03580 | 2.514952 | 0.00015 | 0.000596 | NA |
| AT3G50980 | 2.518324 | 0.00005 | 0.000216 | dehydrin xero 1 |
| AT2G38340 | 2.522243 | 0.00005 | 0.000216 | Integrase-type DNA-binding superfamily protein |
| AT3G52110 | 2.523239 | 0.0115 | 0.027674 | NA |
| AT3G61010 | 2.523457 | 0.00005 | 0.000216 | Ferritin/ribonucleotide reductase-like family protein |
| AT5G24240 | 2.525132 | 0.00005 | 0.000216 | Phosphatidylinositol 3- and 4-kinase ;Ubiquitin family protein |
| AT1G26945 | 2.528977 | 0.00005 | 0.000216 | basic helix-loop-helix (bHLH) DNA-binding superfamily protein |
| AT5G62130 | 2.534 | 0.00005 | 0.000216 | Per1-like family protein |
| AT5G04340 | 2.54078 | 0.00005 | 0.000216 | zinc finger of Arabidopsis thaliana 6 |
| AT1G08980 | 2.541254 | 0.00005 | 0.000216 | amidase 1 |
| AT2G40340 | 2.550071 | 0.00005 | 0.000216 | Integrase-type DNA-binding superfamily protein |
| AT5G17150 | 2.555475 | 0.00365 | 0.010317 | Cystatin/monellin superfamily protein |
| AT2G43580 | 2.559719 | 0.00005 | 0.000216 | Chitinase family protein |
| AT5G23570 | 2.559743 | 0.00005 | 0.000216 | XS domain-containing protein / XS zinc finger domain-containing protein-related |
| AT1G75170 | 2.560473 | 0.00055 | 0.001925 | Sec14p-like phosphatidylinositol transfer family protein |
| AT2G29460 | 2.571332 | 0.00005 | 0.000216 | glutathione S-transferase tau 4 |
| AT4G37760 | 2.572232 | 0.00005 | 0.000216 | squalene epoxidase 3 |
| AT1G08170 | 2.573115 | 0.00005 | 0.000216 | Histone superfamily protein |
| AT2G32830 | 2.578487 | 0.00005 | 0.000216 | phosphate transporter 1;5 |
| AT5G13880 | 2.579786 | 0.0299 | 0.062025 | NA |
| AT1G30550 | 2.580773 | 0.00005 | 0.000216 | S-adenosyl-L-methionine-dependent methyltransferases superfamily protein |
| AT2G38920 | 2.580787 | 0.00005 | 0.000216 | SPX (SYG1/Pho81/XPR1) domain-containing protein / zinc finger (C3HC4-type RING finger) protein-related |
| AT5G19221 | 2.582584 | 0.00025 | 0.000951 | other RNA |
| AT5G55490 | 2.582821 | 0.00005 | 0.000216 | gamete expressed protein 1 |
| AT3G04230 | 2.58975 | 0.00005 | 0.000216 | Ribosomal protein S5 domain 2-like superfamily protein |
| AT5G26340 | 2.589982 | 0.00005 | 0.000216 | Major facilitator superfamily protein |
| AT1G49320 | 2.602644 | 0.00395 | 0.011004 | unknown seed protein like 1 |
| AT1G12320 | 2.613964 | 0.00005 | 0.000216 | Protein of unknown function (DUF1442) |
| AT1G70440 | 2.624184 | 0.011 | 0.026625 | similar to RCD one 3 |
| AT4G29200 | 2.637371 | 0.00005 | 0.000216 | Beta-galactosidase related protein |
| AT2G41280 | 2.643316 | 0.00005 | 0.000216 | late embryogenesis abundant protein (M10) / LEA protein M10 |
| AT5G60270 | 2.650372 | 0.00005 | 0.000216 | Concanavalin A-like lectin protein kinase family protein |
| AT1G69270 | 2.651212 | 0.00005 | 0.000216 | receptor-like protein kinase 1 |
| AT4G10940 | 2.657643 | 0.00005 | 0.000216 | RING/U-box protein |
| AT5G23930 | 2.658704 | 0.00005 | 0.000216 | Mitochondrial transcription termination factor family protein |
| AT5G61590 | 2.665646 | 0.00005 | 0.000216 | Integrase-type DNA-binding superfamily protein |
| AT4G36900 | 2.665981 | 0.00005 | 0.000216 | related to AP2 10 |
| AT1G60970 | 2.670012 | 0.00005 | 0.000216 | SNARE-like superfamily protein |
| AT3G47300 | 2.67117 | 0.00005 | 0.000216 | SELT-like protein precursor |
| AT4G10265 | 2.679602 | 0.00005 | 0.000216 | Wound-responsive family protein |
| AT5G10000 | 2.681057 | 0.02245 | 0.048699 | ferredoxin 4 |
| AT4G03010 | 2.681574 | 0.0132 | 0.03111 | RNI-like superfamily protein |
| AT3G27440 | 2.68484 | 0.0047 | 0.012814 | uridine kinase-like 5 |
| AT5G15950 | 2.685128 | 0.00005 | 0.000216 | Adenosylmethionine decarboxylase family protein |
| AT4G21326 | 2.685517 | 0.00005 | 0.000216 | subtilase 3.12 |
| AT1G46768 | 2.686802 | 0.00005 | 0.000216 | related to AP2 1 |
| AT3G03150 | 2.694502 | 0.00005 | 0.000216 | NA |
| AT1G64820 | 2.701755 | 0.00005 | 0.000216 | MATE efflux family protein |
| AT1G78390 | 2.702463 | 0.00005 | 0.000216 | nine-cis-epoxycarotenoid dioxygenase 9 |
| AT3G02930 | 2.705613 | 0.00005 | 0.000216 | Plant protein of unknown function (DUF827) |
| AT5G40920 | 2.7275 | 0.00005 | 0.000216 | NA |
| AT1G09230 | 2.738794 | 0.00005 | 0.000216 | RNA-binding (RRM/RBD/RNP motifs) family protein |
| AT2G26450 | 2.743007 | 0.00005 | 0.000216 | Plant invertase/pectin methylesterase inhibitor superfamily |
| AT5G05590 | 2.750376 | 0.00005 | 0.000216 | phosphoribosylanthranilate isomerase 2 |
| AT4G16670 | 2.752244 | 0.00005 | 0.000216 | Plant protein of unknown function (DUF828) with plant pleckstrin homology-like region |
| AT3G60980 | 2.755684 | 0.00005 | 0.000216 | Tetratricopeptide repeat (TPR)-like superfamily protein |
| AT4G20260 | 2.758235 | 0.0001 | 0.000412 | plasma-membrane associated cation-binding protein 1 |
| AT1G11125 | 2.759585 | 0.00005 | 0.000216 | NA |
| AT1G71000 | 2.77489 | 0.00005 | 0.000216 | Chaperone DnaJ-domain superfamily protein |
| AT2G46572 | 2.784088 | 0.00105 | 0.003446 | other RNA |
| AT5G15780 | 2.785221 | 0.00005 | 0.000216 | Pollen Ole e 1 allergen and extensin family protein |
| AT1G53560 | 2.785247 | 0.00005 | 0.000216 | Ribosomal protein L18ae family |
| AT1G01580 | 2.791049 | 0.00005 | 0.000216 | ferric reduction oxidase 2 |
| AT1G73190 | 2.79836 | 0.00005 | 0.000216 | Aquaporin-like superfamily protein |
| AT2G47180 | 2.799938 | 0.00005 | 0.000216 | galactinol synthase 1 |
| AT1G68552 | 2.807755 | 0.0002 | 0.000777 | conserved peptide upstream open reading frame 53 |
| AT1G77490 | 2.813532 | 0.00005 | 0.000216 | thylakoidal ascorbate peroxidase |
| AT3G12915 | 2.818704 | 0.00005 | 0.000216 | Ribosomal protein S5/Elongation factor G/III/V family protein |
| AT5G41010 | 2.821795 | 0.00005 | 0.000216 | DNA directed RNA polymerase, 7 kDa subunit |
| AT2G41640 | 2.831649 | 0.00005 | 0.000216 | Glycosyltransferase family 61 protein |
| AT1G22550 | 2.832987 | 0.00235 | 0.007007 | Major facilitator superfamily protein |
| AT5G37000 | 2.845162 | 0.00005 | 0.000216 | Exostosin family protein |
| AT5G10190 | 2.848513 | 0.00005 | 0.000216 | Major facilitator superfamily protein |
| AT5G58575 | 2.856113 | 0.00005 | 0.000216 | NA |
| AT4G35730 | 2.859836 | 0.00005 | 0.000216 | Regulator of Vps4 activity in the MVB pathway protein |
| AT3G12040 | 2.86355 | 0.00005 | 0.000216 | DNA-3-methyladenine glycosylase (MAG) |
| AT5G50770 | 2.866743 | 0.00005 | 0.000216 | hydroxysteroid dehydrogenase 6 |
| AT5G03795 | 2.872522 | 0.00005 | 0.000216 | Exostosin family protein |
| AT2G13960 | 2.875916 | 0.00415 | 0.01148 | Homeodomain-like superfamily protein |
| AT2G29340 | 2.876473 | 0.00005 | 0.000216 | NAD-dependent epimerase/dehydratase family protein |
| AT1G70080 | 2.878447 | 0.0008 | 0.0027 | Terpenoid cyclases/Protein prenyltransferases superfamily protein |
| AT1G21680 | 2.880982 | 0.00005 | 0.000216 | DPP6 N-terminal domain-like protein |
| AT5G18360 | 2.882908 | 0.00005 | 0.000216 | Disease resistance protein (TIR-NBS-LRR class) family |
| AT1G64900 | 2.894873 | 0.00005 | 0.000216 | cytochrome P450, family 89, subfamily A, polypeptide 2 |
| AT3G01850 | 2.899206 | 0.00005 | 0.000216 | Aldolase-type TIM barrel family protein |
| AT1G49310 | 2.904137 | 0.0457 | 0.088714 | NA |
| AT1G11260 | 2.909812 | 0.00005 | 0.000216 | sugar transporter 1 |
| AT5G49760 | 2.912781 | 0.00005 | 0.000216 | Leucine-rich repeat protein kinase family protein |
| AT2G42560 | 2.918527 | 0.00005 | 0.000216 | late embryogenesis abundant domain-containing protein / LEA domain-containing protein |
| AT1G53680 | 2.936016 | 0.00075 | 0.002548 | glutathione S-transferase TAU 28 |
| AT4G14840 | 2.937972 | 0.00005 | 0.000216 | NA |
| AT5G55370 | 2.948819 | 0.00005 | 0.000216 | MBOAT (membrane bound O-acyl transferase) family protein |
| AT3G18485 | 2.986329 | 0.00365 | 0.010317 | iaa-leucine resistant 2 |
| AT5G11110 | 2.988009 | 0.00005 | 0.000216 | sucrose phosphate synthase 2F |
| AT5G25560 | 2.991035 | 0.00005 | 0.000216 | CHY-type/CTCHY-type/RING-type Zinc finger protein |
| AT5G11412 | 2.994677 | 0.00005 | 0.000216 | RNA-binding (RRM/RBD/RNP motifs) family protein |
| AT5G64750 | 3.000989 | 0.00005 | 0.000216 | Integrase-type DNA-binding superfamily protein |
| AT4G11910 | 3.007427 | 0.00005 | 0.000216 | NA |
| AT5G13200 | 3.024922 | 0.00005 | 0.000216 | GRAM domain family protein |
| AT5G27220 | 3.028276 | 0.00005 | 0.000216 | Frigida-like protein |
| AT1G49980 | 3.029336 | 0.00005 | 0.000216 | DNA/RNA polymerases superfamily protein |
| AT4G35190 | 3.036039 | 0.0009 | 0.003 | Putative lysine decarboxylase family protein |
| AT4G22350 | 3.03796 | 0.00005 | 0.000216 | Ubiquitin C-terminal hydrolases superfamily protein |
| AT4G28800 | 3.038455 | 0.00005 | 0.000216 | basic helix-loop-helix (bHLH) DNA-binding superfamily protein |
| AT1G48130 | 3.055653 | 0.00005 | 0.000216 | 1-cysteine peroxiredoxin 1 |
| AT2G39920 | 3.059675 | 0.00005 | 0.000216 | HAD superfamily, subfamily IIIB acid phosphatase |
| AT4G34890 | 3.064253 | 0.00005 | 0.000216 | xanthine dehydrogenase 1 |
| AT1G21140 | 3.065699 | 0.00005 | 0.000216 | Vacuolar iron transporter (VIT) family protein |
| AT1G29680 | 3.069294 | 0.00005 | 0.000216 | Protein of unknown function (DUF1264) |
| AT2G07731 | 3.075758 | 0.00005 | 0.000216 | NA |
| AT5G14930 | 3.082732 | 0.00005 | 0.000216 | senescence-associated gene 101 |
| AT5G31909 | 3.08417 | 0.03365 | 0.068511 | NA |
| AT1G34580 | 3.08948 | 0.00005 | 0.000216 | Major facilitator superfamily protein |
| AT1G05510 | 3.090386 | 0.00005 | 0.000216 | Protein of unknown function (DUF1264) |
| AT2G15970 | 3.090427 | 0.00005 | 0.000216 | cold regulated 413 plasma membrane 1 |
| AT2G31570 | 3.094504 | 0.00005 | 0.000216 | glutathione peroxidase 2 |
| AT1G80110 | 3.098214 | 0.00005 | 0.000216 | phloem protein 2-B11 |
| AT1G70840 | 3.112548 | 0.00005 | 0.000216 | MLP-like protein 31 |
| AT5G45150 | 3.117409 | 0.00005 | 0.000216 | RNAse THREE-like protein 3 |
| AT5G09950 | 3.127865 | 0.00005 | 0.000216 | Tetratricopeptide repeat (TPR)-like superfamily protein |
| AT4G30470 | 3.128853 | 0.00005 | 0.000216 | NAD(P)-binding Rossmann-fold superfamily protein |
| AT1G60590 | 3.130856 | 0.00275 | 0.008049 | Pectin lyase-like superfamily protein |
| AT1G52855 | 3.154813 | 0.00005 | 0.000216 | NA |
| AT1G16850 | 3.15492 | 0.00005 | 0.000216 | NA |
| AT4G23180 | 3.157346 | 0.0013 | 0.004171 | cysteine-rich RLK (RECEPTOR-like protein kinase) 10 |
| AT5G42840 | 3.159317 | 0.00005 | 0.000216 | Cysteine/Histidine-rich C1 domain family protein |
| AT4G33666 | 3.178618 | 0.00075 | 0.002548 | NA |
| AT2G45910 | 3.18096 | 0.00005 | 0.000216 | U-box domain-containing protein kinase family protein |
| AT5G18180 | 3.191384 | 0.00005 | 0.000216 | H/ACA ribonucleoprotein complex, subunit Gar1/Naf1 protein |
| AT1G08630 | 3.195534 | 0.00005 | 0.000216 | threonine aldolase 1 |
| AT3G21020 | 3.215783 | 0.00005 | 0.000216 | transposable element gene |
| AT5G07060 | 3.21993 | 0.0023 | 0.006876 | CCCH-type zinc fingerfamily protein with RNA-binding domain |
| AT3G51890 | 3.23891 | 0.00005 | 0.000216 | Clathrin light chain protein |
| AT3G19290 | 3.24357 | 0.00005 | 0.000216 | ABRE binding factor 4 |
| AT5G44040 | 3.259458 | 0.00005 | 0.000216 | NA |
| AT4G18660 | 3.269227 | 0.00005 | 0.000216 | NA |
| AT3G27630 | 3.275835 | 0.0107 | 0.025972 | NA |
| AT2G45360 | 3.291343 | 0.0069 | 0.017847 | Protein of unknown function (DUF1442) |
| AT1G76180 | 3.306771 | 0.00005 | 0.000216 | Dehydrin family protein |
| AT1G78020 | 3.31456 | 0.00175 | 0.005406 | Protein of unknown function (DUF581) |
| AT4G08770 | 3.316828 | 0.00005 | 0.000216 | Peroxidase superfamily protein |
| AT2G19900 | 3.329895 | 0.00005 | 0.000216 | NADP-malic enzyme 1 |
| AT3G45650 | 3.330702 | 0.00005 | 0.000216 | nitrate excretion transporter1 |
| AT1G34460 | 3.341776 | 0.00165 | 0.005138 | CYCLIN B1;5 |
| AT5G33300 | 3.348307 | 0.00005 | 0.000216 | chromosome-associated kinesin-related |
| AT5G30500 | 3.365435 | 0.00395 | 0.011004 | Nucleotide-diphospho-sugar transferases superfamily protein |
| AT2G24310 | 3.366682 | 0.00005 | 0.000216 | NA |
| AT3G23450 | 3.367121 | 0.00005 | 0.000216 | NA |
| AT3G10150 | 3.380804 | 0.0035 | 0.009954 | purple acid phosphatase 16 |
| AT5G66400 | 3.383237 | 0.00005 | 0.000216 | Dehydrin family protein |
| AT3G63280 | 3.391312 | 0.00005 | 0.000216 | NIMA-related kinase 4 |
| AT2G21780 | 3.397312 | 0.00005 | 0.000216 | NA |
| AT5G62800 | 3.397822 | 0.00005 | 0.000216 | Protein with RING/U-box and TRAF-like domains |
| AT5G52300 | 3.398483 | 0.00005 | 0.000216 | CAP160 protein |
| AT5G62820 | 3.407473 | 0.0015 | 0.004727 | Uncharacterised protein family (UPF0497) |
| AT2G27010 | 3.412256 | 0.00005 | 0.000216 | cytochrome P450, family 705, subfamily A, polypeptide 9 |
| AT2G02930 | 3.416859 | 0.00005 | 0.000216 | glutathione S-transferase F3 |
| AT4G18150 | 3.429841 | 0.00065 | 0.002236 | Kinase-related protein of unknown function (DUF1296) |
| AT3G10010 | 3.433666 | 0.00005 | 0.000216 | demeter-like 2 |
| AT5G52310 | 3.469041 | 0.00005 | 0.000216 | low-temperature-responsive protein 78 (LTI78) / desiccation-responsive protein 29A (RD29A) |
| AT4G19990 | 3.484487 | 0.00005 | 0.000216 | FAR1-related sequence 1 |
| AT5G58670 | 3.492479 | 0.00045 | 0.001612 | phospholipase C1 |
| AT1G77950 | 3.498761 | 0.00005 | 0.000216 | AGAMOUS-like 67 |
| AT5G17700 | 3.509696 | 0.00005 | 0.000216 | MATE efflux family protein |
| AT2G20720 | 3.511655 | 0.00005 | 0.000216 | Pentatricopeptide repeat (PPR) superfamily protein |
| AT5G24280 | 3.513788 | 0.00005 | 0.000216 | gamma-irradiation and mitomycin c induced 1 |
| AT4G28040 | 3.524083 | 0.00155 | 0.004866 | nodulin MtN21 /EamA-like transporter family protein |
| AT4G15490 | 3.526172 | 0.00005 | 0.000216 | UDP-Glycosyltransferase superfamily protein |
| AT4G34480 | 3.527672 | 0.00005 | 0.000216 | O-Glycosyl hydrolases family 17 protein |
| AT5G51210 | 3.530905 | 0.00005 | 0.000216 | oleosin3 |
| AT1G16550 | 3.532654 | 0.00005 | 0.000216 | NA |
| AT2G30250 | 3.53848 | 0.00005 | 0.000216 | WRKY DNA-binding protein 25 |
| AT5G45310 | 3.549073 | 0.00005 | 0.000216 | NA |
| AT3G61410 | 3.569385 | 0.01425 | 0.033194 | NA |
| AT3G25670 | 3.592228 | 0.00095 | 0.003145 | Leucine-rich repeat (LRR) family protein |
| AT4G06701 | 3.592263 | 0.00005 | 0.000216 | other RNA |
| AT1G54290 | 3.610744 | 0.00005 | 0.000216 | Translation initiation factor SUI1 family protein |
| AT5G58350 | 3.612429 | 0.00005 | 0.000216 | with no lysine (K) kinase 4 |
| AT4G21323 | 3.622654 | 0.00005 | 0.000216 | Subtilase family protein |
| AT5G44360 | 3.629122 | 0.00005 | 0.000216 | FAD-binding Berberine family protein |
| AT3G03776 | 3.636425 | 0.00035 | 0.001288 | hydroxyproline-rich glycoprotein family protein |
| AT3G09910 | 3.638177 | 0.00005 | 0.000216 | RAB GTPase homolog C2B |
| AT1G54860 | 3.651647 | 0.00005 | 0.000216 | Glycoprotein membrane precursor GPI-anchored |
| AT1G73620 | 3.658377 | 0.00005 | 0.000216 | Pathogenesis-related thaumatin superfamily protein |
| AT5G45840 | 3.680934 | 0.00005 | 0.000216 | Leucine-rich repeat protein kinase family protein |
| AT3G44720 | 3.707948 | 0.00005 | 0.000216 | arogenate dehydratase 4 |
| AT1G36920 | 3.709475 | 0.0171 | 0.038711 | NA |
| AT5G41660 | 3.744386 | 0.0018 | 0.00555 | NA |
| AT4G36220 | 3.753816 | 0.00005 | 0.000216 | ferulic acid 5-hydroxylase 1 |
| AT2G40880 | 3.778641 | 0.00005 | 0.000216 | cystatin A |
| AT2G47770 | 3.800723 | 0.00005 | 0.000216 | TSPO(outer membrane tryptophan-rich sensory protein)-related |
| AT4G13560 | 3.802514 | 0.0444 | 0.086619 | Late embryogenesis abundant protein (LEA) family protein |
| AT2G45920 | 3.806127 | 0.00005 | 0.000216 | U-box domain-containing protein |
| AT1G67800 | 3.810218 | 0.00005 | 0.000216 | Copine (Calcium-dependent phospholipid-binding protein) family |
| AT5G46880 | 3.872582 | 0.00005 | 0.000216 | homeobox-7 |
| AT3G18300 | 3.873879 | 0.00805 | 0.020376 | NA |
| AT2G25964 | 3.88303 | 0.00005 | 0.000216 | NA |
| AT3G22240 | 3.935029 | 0.00955 | 0.023619 | NA |
| AT1G26530 | 3.938452 | 0.00005 | 0.000216 | PIN domain-like family protein |
| AT1G26960 | 3.940482 | 0.01415 | 0.03297 | homeobox protein 23 |
| AT5G16460 | 3.940853 | 0.00005 | 0.000216 | Putative adipose-regulatory protein (Seipin) |
| AT5G27420 | 3.965062 | 0.00005 | 0.000216 | carbon/nitrogen insensitive 1 |
| AT4G36700 | 3.988652 | 0.00005 | 0.000216 | RmlC-like cupins superfamily protein |
| AT5G38280 | 4.030297 | 0.00005 | 0.000216 | PR5-like receptor kinase |
| AT3G03640 | 4.069248 | 0.00005 | 0.000216 | beta glucosidase 25 |
| AT5G65590 | 4.069724 | 0.00005 | 0.000216 | Dof-type zinc finger DNA-binding family protein |
| AT3G17220 | 4.072486 | 0.0337 | 0.068588 | pectin methylesterase inhibitor 2 |
| AT3G61970 | 4.084675 | 0.00005 | 0.000216 | AP2/B3-like transcriptional factor family protein |
| AT4G34332 | 4.092703 | 0.0205 | 0.045171 | other RNA |
| AT3G13400 | 4.102822 | 0.00005 | 0.000216 | SKU5 similar 13 |
| AT2G35690 | 4.11487 | 0.00005 | 0.000216 | acyl-CoA oxidase 5 |
| AT5G16990 | 4.115848 | 0.00005 | 0.000216 | Zinc-binding dehydrogenase family protein |
| AT3G48580 | 4.128335 | 0.00005 | 0.000216 | xyloglucan endotransglucosylase/hydrolase 11 |
| AT3G10120 | 4.141969 | 0.01635 | 0.037261 | NA |
| AT1G05340 | 4.142405 | 0.00005 | 0.000216 | NA |
| AT2G19400 | 4.145567 | 0.00005 | 0.000216 | AGC (cAMP-dependent, cGMP-dependent and protein kinase C) kinase family protein |
| AT5G56540 | 4.145897 | 0.0324 | 0.06645 | arabinogalactan protein 14 |
| AT1G79360 | 4.148197 | 0.00005 | 0.000216 | organic cation/carnitine transporter 2 |
| AT3G26165 | 4.175664 | 0.00005 | 0.000216 | NA |
| AT2G39800 | 4.194564 | 0.00005 | 0.000216 | delta1-pyrroline-5-carboxylate synthase 1 |
| AT4G36150 | 4.291016 | 0.00005 | 0.000216 | Disease resistance protein (TIR-NBS-LRR class) family |
| AT3G45440 | 4.342951 | 0.00005 | 0.000216 | Concanavalin A-like lectin protein kinase family protein |
| AT4G18330 | 4.352003 | 0.0002 | 0.000777 | Translation elongation factor EF1A/initiation factor IF2gamma family protein |
| AT2G23330 | 4.369123 | 0.00005 | 0.000216 | transposable element gene |
| AT1G52880 | 4.405707 | 0.007 | 0.018073 | NAC (No Apical Meristem) domain transcriptional regulator superfamily protein |
| AT5G14870 | 4.406017 | 0.00005 | 0.000216 | cyclic nucleotide-gated channel 18 |
| AT1G77450 | 4.409938 | 0.00005 | 0.000216 | NAC domain containing protein 32 |
| AT3G48390 | 4.420517 | 0.00005 | 0.000216 | MA3 domain-containing protein |
| AT1G66360 | 4.464419 | 0.00005 | 0.000216 | Calcium-dependent lipid-binding (CaLB domain) family protein |
| AT1G65970 | 4.488971 | 0.00005 | 0.000216 | thioredoxin-dependent peroxidase 2 |
| AT5G65280 | 4.520534 | 0.00005 | 0.000216 | GCR2-like 1 |
| AT3G48230 | 4.548594 | 0.00045 | 0.001612 | Protein of Unknown Function (DUF239) |
| AT2G03520 | 4.556479 | 0.00005 | 0.000216 | ureide permease 4 |
| AT5G19100 | 4.595625 | 0.00005 | 0.000216 | Eukaryotic aspartyl protease family protein |
| AT4G15236 | 4.608414 | 0.00005 | 0.000216 | ABC-2 and Plant PDR ABC-type transporter family protein |
| AT5G18130 | 4.64041 | 0.00005 | 0.000216 | NA |
| AT3G21730 | 4.657374 | 0.00005 | 0.000216 | Dihydroneopterin aldolase |
| AT2G35736 | 4.729969 | 0.00005 | 0.000216 | NA |
| AT3G08885 | 4.773648 | 0.02845 | 0.059583 | NA |
| AT3G26290 | 4.804649 | 0.00005 | 0.000216 | cytochrome P450, family 71, subfamily B, polypeptide 26 |
| AT3G05260 | 4.815652 | 0.00005 | 0.000216 | NAD(P)-binding Rossmann-fold superfamily protein |
| AT3G53040 | 4.894527 | 0.00005 | 0.000216 | late embryogenesis abundant protein, putative / LEA protein, putative |
| AT1G09950 | 4.939041 | 0.0002 | 0.000777 | RESPONSE TO ABA AND SALT 1 |
| AT3G26840 | 4.949834 | 0.00005 | 0.000216 | Esterase/lipase/thioesterase family protein |
| AT2G05290 | 4.98055 | 0.0272 | 0.05731 | transposable element gene |
| AT1G57790 | 5.125672 | 0.00005 | 0.000216 | F-box family protein |
| AT2G29950 | 5.126974 | 0.0003 | 0.00112 | ELF4-like 1 |
| AT4G26466 | 5.128475 | 0.01385 | 0.032386 | lorelei |
| AT1G09180 | 5.290735 | 0.00005 | 0.000216 | secretion-associated RAS super family 1 |
| AT3G49830 | 5.352605 | 0.018 | 0.040415 | P-loop containing nucleoside triphosphate hydrolases superfamily protein |
| AT5G55780 | 5.355455 | 0.00025 | 0.000951 | Cysteine/Histidine-rich C1 domain family protein |
| AT5G28626 | 5.384163 | 0.00005 | 0.000216 | transposable element gene |
| AT5G11410 | 5.42763 | 0.00005 | 0.000216 | Protein kinase superfamily protein |
| AT2G43010 | 5.457873 | 0.00005 | 0.000216 | phytochrome interacting factor 4 |
| AT4G31730 | 5.489565 | 0.00005 | 0.000216 | glutamine dumper 1 |
| AT4G38080 | 5.519925 | 0.00005 | 0.000216 | hydroxyproline-rich glycoprotein family protein |
| AT5G50335 | 5.581978 | 0.00025 | 0.000951 | NA |
| AT4G26220 | 5.647233 | 0.00005 | 0.000216 | S-adenosyl-L-methionine-dependent methyltransferases superfamily protein |
| AT2G19910 | 5.70529 | 0.00005 | 0.000216 | RNA-dependent RNA polymerase family protein |
| AT1G20120 | 5.748395 | 0.00125 | 0.004021 | GDSL-like Lipase/Acylhydrolase superfamily protein |
| AT5G13210 | 5.790571 | 0.00005 | 0.000216 | Uncharacterised conserved protein UCP015417, vWA |
| AT2G01275 | 5.799753 | 0.01215 | 0.029 | RING/FYVE/PHD zinc finger superfamily protein |
| AT5G57480 | 5.886248 | 0.00005 | 0.000216 | P-loop containing nucleoside triphosphate hydrolases superfamily protein |
| AT4G01026 | 5.924207 | 0.00005 | 0.000216 | PYR1-like 7 |
| AT3G51895 | 5.925299 | 0.00005 | 0.000216 | sulfate transporter 3;1 |
| AT5G07190 | 5.929417 | 0.00005 | 0.000216 | seed gene 3 |
| AT3G07260 | 5.98639 | 0.00005 | 0.000216 | SMAD/FHA domain-containing protein |
| AT2G46680 | 5.995016 | 0.00005 | 0.000216 | homeobox 7 |
| AT4G13575 | 6.031935 | 0.00005 | 0.000216 | NA |
| AT1G20190 | 6.092771 | 0.0081 | 0.020481 | expansin 11 |
| AT1G30040 | 6.144833 | 0.00005 | 0.000216 | gibberellin 2-oxidase |
| AT5G57810 | 6.178044 | 0.00005 | 0.000216 | tetraspanin15 |
| AT2G06002 | 6.288009 | 0.00005 | 0.000216 | other RNA |
| AT1G25310 | 6.304838 | 0.00005 | 0.000216 | basic helix-loop-helix (bHLH) DNA-binding family protein |
| AT5G07380 | 6.357617 | 0.00005 | 0.000216 | NA |
| AT5G17260 | 6.397978 | 0.00035 | 0.001288 | NAC domain containing protein 86 |
| AT1G65130 | 6.487968 | 0.00005 | 0.000216 | Ubiquitin carboxyl-terminal hydrolase-related protein |
| AT4G18690 | 6.546774 | 0.00005 | 0.000216 | NA |
| AT1G77530 | 6.766594 | 0.00005 | 0.000216 | O-methyltransferase family protein |
| AT2G04110 | 6.783348 | 0.0023 | 0.006876 | NA |
| AT1G65110 | 6.81162 | 0.00005 | 0.000216 | Ubiquitin carboxyl-terminal hydrolase-related protein |
| AT2G41470 | 6.914907 | 0.00005 | 0.000216 | NA |
| AT5G16960 | 6.958929 | 0.00005 | 0.000216 | Zinc-binding dehydrogenase family protein |
| AT4G25850 | 7.011217 | 0.00005 | 0.000216 | OSBP(oxysterol binding protein)-related protein 4B |
| AT1G06100 | 7.175056 | 0.01005 | 0.024633 | Fatty acid desaturase family protein |
| AT1G20490 | 7.312125 | 0.00005 | 0.000216 | AMP-dependent synthetase and ligase family protein |
| AT5G64410 | 7.312779 | 0.00005 | 0.000216 | oligopeptide transporter 4 |
| AT3G03240 | 7.338623 | 0.0021 | 0.006356 | alpha/beta-Hydrolases superfamily protein |
| AT5G45440 | 7.344595 | 0.00005 | 0.000216 | P-loop containing nucleoside triphosphate hydrolases superfamily protein |
| AT2G14210 | 7.491954 | 0.00005 | 0.000216 | AGAMOUS-like 44 |
| AT3G23050 | 7.704823 | 0.00005 | 0.000216 | indole-3-acetic acid 7 |
| AT3G47540 | 7.787005 | 0.0001 | 0.000412 | Chitinase family protein |
| AT1G35720 | 7.808748 | 0.00005 | 0.000216 | annexin 1 |
| AT3G58865 | 7.880043 | 0.00005 | 0.000216 | transposable element gene |
| AT1G13340 | 8.625888 | 0.00005 | 0.000216 | Regulator of Vps4 activity in the MVB pathway protein |
| AT5G45830 | 8.757892 | 0.00005 | 0.000216 | delay of germination 1 |
| AT1G77960 | 8.869236 | 0.01365 | 0.032015 | NA |
| AT1G31200 | 9.12058 | 0.0071 | 0.018281 | phloem protein 2-A9 |
| AT3G43270 | 9.448611 | 0.00005 | 0.000216 | Plant invertase/pectin methylesterase inhibitor superfamily |
| AT5G14500 | 9.458753 | 0.00005 | 0.000216 | aldose 1-epimerase family protein |
| AT5G55020 | 9.585607 | 0.0001 | 0.000412 | myb domain protein 120 |
| AT1G11370 | 9.628049 | 0.0479 | 0.092055 | Pectin lyase-like superfamily protein |
| AT4G15450 | 9.729873 | 0.00005 | 0.000216 | Senescence/dehydration-associated protein-related |
| AT5G57550 | 10.09329 | 0.00005 | 0.000216 | xyloglucan endotransglucosylase/hydrolase 25 |
| AT5G40010 | 10.21666 | 0.00005 | 0.000216 | AAA-ATPase 1 |
| AT5G03820 | 10.26989 | 0.0267 | 0.056403 | GDSL-like Lipase/Acylhydrolase family protein |
| AT2G19850 | 10.9701 | 0.0002 | 0.000777 | NA |
| AT2G40960 | 11.00681 | 0.00005 | 0.000216 | Single-stranded nucleic acid binding R3H protein |
| AT2G35810 | 11.12598 | 0.00005 | 0.000216 | NA |
| AT4G01525 | 11.30378 | 0.00005 | 0.000216 | transposable element gene |
| AT4G33270 | 11.48078 | 0.0042 | 0.011609 | Transducin family protein / WD-40 repeat family protein |
| AT1G22980 | 12.48329 | 0.00005 | 0.000216 | NA |
| AT1G22380 | 13.32121 | 0.00395 | 0.011004 | UDP-glucosyl transferase 85A3 |
| AT3G45000 | 13.46545 | 0.00005 | 0.000216 | SNF7 family protein |
| AT3G16410 | 13.79279 | 0.00005 | 0.000216 | nitrile specifier protein 4 |
| AT5G40320 | 14.25581 | 0.0029 | 0.008425 | Cysteine/Histidine-rich C1 domain family protein |
| AT1G80555 | 14.44591 | 0.00005 | 0.000216 | Isocitrate/isopropylmalate dehydrogenase family protein |
| AT5G51390 | 14.86954 | 0.02915 | 0.060802 | NA |
| AT1G51538 | 15.16424 | 0.00265 | 0.007791 | Aminotransferase-like, plant mobile domain family protein |
| AT2G05914 | 15.27581 | 0.00005 | 0.000216 | NA |
| AT2G39110 | 17.18948 | 0.00005 | 0.000216 | Protein kinase superfamily protein |
| AT4G11911 | 17.28704 | 0.0026 | 0.007668 | NA |
| AT1G63390 | 17.35334 | 0.00265 | 0.007791 | FAD/NAD(P)-binding oxidoreductase family protein |
| AT1G61440 | 17.91196 | 0.00005 | 0.000216 | S-locus lectin protein kinase family protein |
| AT5G66840 | 18.74974 | 0.0026 | 0.007668 | SAP domain-containing protein |
| AT4G08430 | 18.94967 | 0.0001 | 0.000412 | Ulp1 protease family protein |
| AT5G34871 | 19.22797 | 0.00005 | 0.000216 | other RNA |
| AT4G37620 | 21.15011 | 0.044 | 0.085996 | transposable element gene |
| AT1G64100 | 21.16553 | 0.0108 | 0.0262 | pentatricopeptide (PPR) repeat-containing protein |
| AT2G05642 | 21.47773 | 0.0026 | 0.007668 | Nucleic acid-binding, OB-fold-like protein |
| AT1G69480 | 22.40019 | 0.00005 | 0.000216 | EXS (ERD1/XPR1/SYG1) family protein |
| AT5G65090 | 22.64519 | 0.0371 | 0.074248 | DNAse I-like superfamily protein |
| AT3G44716 | 25.06356 | 0.00005 | 0.000216 | NA |
| AT3G56890 | 26.09942 | 0.01835 | 0.041125 | F-box associated ubiquitination effector family protein |
| AT2G05915 | 29.83835 | 0.00005 | 0.000216 | NA |
| AT4G10260 | 32.83636 | 0.00005 | 0.000216 | pfkB-like carbohydrate kinase family protein |
| AT1G23120 | 33.04398 | 0.0001 | 0.000412 | Polyketide cyclase/dehydrase and lipid transport superfamily protein |
| AT3G06437 | 35.00741 | 0.0001 | 0.000412 | NA |
| AT5G17100 | 42.59178 | 0.0026 | 0.007668 | Cystatin/monellin superfamily protein |
| AT3G24255 | 43.28839 | 0.00005 | 0.000216 | RNA-directed DNA polymerase (reverse transcriptase)-related family protein |
| AT1G50290 | 50.3468 | 0.00005 | 0.000216 | NA |
| AT1G65200 | 55.23284 | 0.00005 | 0.000216 | Ubiquitin carboxyl-terminal hydrolase-related protein |
| AT4G24420 | 93.44175 | 0.0001 | 0.000412 | RNA-binding (RRM/RBD/RNP motifs) family protein |
| AT4G08093 | 114.5708 | 0.0026 | 0.007668 | NA |
| AT3G48300 | 134.5101 | 0.0254 | 0.054052 | cytochrome P450, family 71, subfamily A, polypeptide 23 |
| AT1G12340 | 147.0754 | 0.044 | 0.085996 | Cornichon family protein |
| AT2G15555 | 550.6062 | 0.00005 | 0.000216 | other RNA |
| AT3G60955 | 640.2665 | 0.00385 | 0.010775 | NA |
| AT5G33251 | 687.9695 | 0.044 | 0.085996 | NA |
| AT1G61510 | 903.631 | 0.00005 | 0.000216 | transposable element gene |
| AT4G06752 | 909.878 | 0.00005 | 0.000216 | transposable element gene |
| AT2G11280 | 991.431 | 0.00005 | 0.000216 | NA |
| AT4G10990 | 997.353 | 0.00005 | 0.000216 | transposable element gene |
| AT2G16490 | 1061.1 | 0.00005 | 0.000216 | XH domain-containing protein |
| AT5G47260 | 1236.25 | 0.00005 | 0.000216 | ATP binding;GTP binding;nucleotide binding;nucleoside-triphosphatases |
| AT4G14670 | 1307.71 | 0.00005 | 0.000216 | casein lytic proteinase B2 |
| AT4G22450 | 1577.17 | 0.00005 | 0.000216 | transposable element gene |
| AT3G43950 | 1670.36 | 0.00005 | 0.000216 | Protein kinase superfamily protein |
| AT1G60020 | 1844.21 | 0.00005 | 0.000216 | transposable element gene |
| AT2G01360 | 2031.89 | 0.00005 | 0.000216 | pentatricopeptide (PPR) repeat-containing protein |
| AT2G05995 | 2114.63 | 0.00005 | 0.000216 | other RNA |
| AT4G10240 | 2556.52 | 0.00005 | 0.000216 | B-box zinc finger family protein |
| AT1G71920 | 2577.29 | 0.00005 | 0.000216 | HISTIDINE BIOSYNTHESIS 6B |
| AT4G10980 | 2976.55 | 0.00005 | 0.000216 | transposable element gene |
| AT5G26642 | 3334.25 | 0.00005 | 0.000216 | transposable element gene |
| AT5G15360 | 3418.9 | 0.00005 | 0.000216 | NA |
| AT1G63320 | 3482.49 | 0.00005 | 0.000216 | Pentatricopeptide repeat (PPR) superfamily protein |
| AT4G36791 | 4916.74 | 0.00025 | 0.000951 | NA |
| AT3G44660 | 5117.74 | 0.00005 | 0.000216 | histone deacetylase 10 |
| AT5G36240 | 5783.17 | 0.00005 | 0.000216 | zinc knuckle (CCHC-type) family protein |
| AT5G66970 | 5871.65 | 0.00005 | 0.000216 | P-loop containing nucleoside triphosphate hydrolases superfamily protein |
| AT2G24692 | 8554.67 | 0.00005 | 0.000216 | NA |
| AT5G15420 | 8992.48 | 0.00005 | 0.000216 | NA |
| AT3G44490 | 9227.11 | 0.00005 | 0.000216 | histone deacetylase 17 |
| AT4G19239 | 119860 | 0.00005 | 0.000216 | NA |

**Supplemental Table S11**. Expression change of *EM1* and *EM6* in *abi5-1* seeds.

| **Gene_ID** | **Fold change (*abi5-1*/Col-0)** | **P-value** | **FDR** | **Gene description** |
| --- | --- | --- | --- | --- |
|
| AT3G51810 | 0.169401073 | 5.00E-05 | 0.000216 | EM1, Stress induced protein |
| AT2G40170 | 0.477626049 | 5.00E-05 | 0.000216 | EM6, Stress induced protein |
